# Global Existence and Nonlinear Stability of Finite-Energy Solutions of the Compressible Euler–Riesz Equations with Large Initial Data of Spherical Symmetry

**DOI:** 10.1007/s00220-026-05659-5

**Published:** 2026-08-28

**Authors:** José A. Carrillo, Samuel R. Charles, Gui-Qiang G. Chen, Difan Yuan

**Affiliations:** 1 Mathematical Institute, University of Oxford, Oxford, OX2 6GG UK; 2 Academy of Mathematics and Systems Science, Chinese Academy of Sciences, Beijing, 100190 China; 3 School of Mathematical Sciences, Beijing Normal University, Beijing, 100875 China

## Abstract

The compressible Euler–Riesz equations are fundamental with wide applications in astrophysics, plasma physics, and mathematical biology. In this paper, we are concerned with the global existence and nonlinear stability of finite-energy solutions of the multidimensional Euler–Riesz equations with large initial data of spherical symmetry. We consider both attractive and repulsive interactions for a wide range of $$ {Riesz}$$ and $$ {logarithmic}$$ potentials for dimensions larger than or equal to two. This is achieved by the inviscid limit of the solutions of the corresponding Cauchy problem for the Navier–Stokes–Riesz equations. The strong convergence of the vanishing viscosity solutions is achieved through delicate uniform estimates in $$L^p$$. It is observed that, even if the attractive potential is super-Coulomb, no concentration is formed near the origin in the inviscid limit. Moreover, we prove that the nonlinear stability of global finite-energy solutions for the Euler–Riesz equations is $$ {unconditional}$$ under a spherically symmetric perturbation around the steady solutions. Unlike the Coulomb case where the potential can be represented locally, the singularity and regularity of the nonlocal radial Riesz potential near the origin require careful analysis, which is a crucial step. We also establish uniform energy estimates of global solutions for not only the Riesz potential but also the limiting end-point case: the logarithmic potential. Finally, unlike the Coulomb case, a Grönwall type estimate is required to overcome the difficulty of the appearance of boundary terms in the sub-Coulomb case and the singularity of the super-Coulomb potential. Furthermore, we prove the nonlinear stability of global finite-energy solutions for the compressible Euler–Riesz equations around steady states by employing concentration compactness arguments. Steady states properties are obtained by variational arguments connecting to recent advances in aggregation-diffusion equations.

## Introduction

We are concerned with the global existence and nonlinear stability of spherically symmetric solutions of the multi-dimensional (M-D) compressible Euler–Riesz equations (CEREs) with large initial data. The equations are of the form:

1.1 $$\begin{aligned} {\left\{ \begin{array}{ll} \displaystyle \partial _t \rho +\nabla \cdot {\mathcal {M}}=0, \\ \displaystyle \partial _t {\mathcal {M}}+ \nabla \cdot \Big (\frac{{\mathcal {M}}\otimes {\mathcal {M}}}{\rho }\Big ) +\nabla p + \kappa \rho \nabla \mathcal {W}_\alpha = \boldsymbol{0}, \end{array}\right. } \end{aligned}$$

for t>0, $\boldsymbol{x} \in \mathbb {R}^n$, and n≥2, where $\rho : [0,\infty ) \times \mathbb {R}^n \rightarrow \mathbb {R}_+$ is the density, ${\mathcal {M}}: [0,\infty ) \times \mathbb {R}^n \rightarrow \mathbb {R}^n$ is the momentum, and $p: [0,\infty ) \times \mathbb {R}^n \rightarrow \mathbb {R}$ is the pressure that is related to the density via a power law:

$$ p = p(\rho ) = a_0 \rho ^\gamma $$

for the adiabatic exponent γ>1. By scaling, we may choose $a_0 = \frac{(\gamma -1)^2}{4 \gamma } > 0$. $\mathcal {W}_\alpha : \mathbb {R}^n \rightarrow \mathbb {R}$ is the Riesz potential for α>0 and the logarithmic potential for α=0. In this paper, we are particularly interested in a specific region $\alpha \in (-1,n)$ so that $\mathcal {W}_\alpha$ is given by $\mathcal {W}_\alpha =\Phi _\alpha *\rho$ and

1.2 $$\begin{aligned} \nabla \mathcal {W}_\alpha (\boldsymbol{x}) = {\left\{ \begin{array}{ll} \displaystyle (\nabla \Phi _\alpha * \rho ) (\boldsymbol{x}) &  \text {for } \alpha \in (-1,n-1), \\ \displaystyle \int _{\mathbb {R}^n} \nabla \Phi _\alpha (\boldsymbol{x} - \boldsymbol{y}) (\rho (\boldsymbol{y}) - \rho (\boldsymbol{x})) {\, \mathrm d}\boldsymbol{y} \quad &  \text {for } \alpha \in [n-1,n), \end{array}\right. } \end{aligned}$$

where $\Phi _\alpha : \mathbb {R}^n \rightarrow \mathbb {R}$ is a Riesz kernel for $\alpha \in (0,n)$ and a logarithmic kernel for α=0; that is, the kernel given by

$$ \Phi _\alpha (\boldsymbol{x}) = {\left\{ \begin{array}{ll} -\frac{|\boldsymbol{x}|^{-\alpha }}{\alpha } & \quad \text {for } \alpha \ne 0, \\ \log |\boldsymbol{x}| & \quad \text {for } \alpha = 0. \end{array}\right. } $$

Here, the logarithmic kernel can be obtained as a limit of the Riesz kernels, as $\alpha \rightarrow 0$, in the sense that

1.3 $$\begin{aligned} \lim _{\alpha \rightarrow 0} \big (\Phi _\alpha (\boldsymbol{x})+ \frac{1}{\alpha }\big ) = \lim _{\alpha \rightarrow 0} \frac{1-|\boldsymbol{x}|^{-\alpha }}{\alpha } = \log |\boldsymbol{x}|. \end{aligned}$$

Whenever α appears, we use the convention that α=0 represents the logarithmic case. The range $$\alpha \in (-1,n)$$ includes the important Coulomb case α=n-2. κ>0 represents the attractive potential case, and κ<0 represents the repulsive potential case. By scaling without loss of generality, we can choose $$\kappa \in \{-1,1\}$$. When α<n, the kernel $$\Phi _\alpha $$ is integrable near 0 and hence can be analyzed via the mean-field theory and the potential theory. However, for $$\alpha \ge n$$, the kernel $$\Phi _\alpha $$ is not integrable near the origin so that the potential theory is not amenable, in which case the kernel is called $$ {hypersingular}$$ and behaves more like a short-range interaction problem; see [57].

Riesz gases arise in many different areas of physics: To list a few, plasma physics, star or galaxy dynamics, solid state physics, ferrofluids, elasticity, and random matrices; see [13]. The jellium and uniform electron gas have also been studied for the Riesz interaction; see [35, 65]. We refer to the recent survey [66] with many open problems. There are also applications in mathematical biology such as swarming models; see [15, 16].

In the case of the super-Coulomb Riesz potential, for $\alpha \in (n-2,n)$, $\Phi _\alpha$ is known to be the kernel of the fractional Laplacian operator:

$$ (-\Delta )^\frac{n-\alpha }{2} \mathcal {W}_\alpha = - c_{n,\alpha } \rho $$

in the sense of distributions, where

$$ c_{n,\alpha } = {\left\{ \begin{array}{ll} \frac{2^{n-\alpha } \pi ^\frac{n}{2} \Gamma \left( \frac{n-\alpha }{2} \right) }{\alpha \Gamma \left( \frac{\alpha }{2} \right) } & \quad \text {for } \alpha > \max \{0, n-2\}, \\ 2\pi & \quad \text {for } \alpha = 0 \text { and } n = 2; \end{array}\right. } $$

see [44] for the details of the calculation. The fractional Laplacian is a non-local operator and can be defined by Fourier multipliers or in the real space through a singular integral. Serfaty [82] first proved that the Euler equations of form (1.1) in the pressure-less case and repulsive regime, with Coulomb or super-Coulomb Riesz potential, can be derived via the mean-field limits of particle systems. Then Nguyen-Rosenzweig-Serfaty [78] treated the whole range $\alpha \in [0,n)$ by developing a modulated-energy approach; see also Serfaty [83].

For α=0, $\Phi _0(\boldsymbol{x}) = \log |\boldsymbol{x}|$. This interaction potential is called the logarithmic potential. This case is ubiquitous, with many applications in statistical and quantum mechanics, random matrix theory, self-avoiding random walks, random tilings, and the proofs of functional inequalities; see [3, 66] for more details.

For α=n-2 and n≥3, the interaction potential is called the Coulomb potential. The Coulomb gas can be used as a toy model to study classical matters without the effects of quantum mechanics. To give an example, Gamow’s *liquid drop model* is a simplified model used to study atomic nuclei, electrons, and atoms; see [83]. In the case when one phase is dominant, this can be reduced to a system of particles interacting via a Coulomb potential; see [1]. Since the 1970 s, the Coulomb gas has been regarded as an incredibly important area of study in the field of statistical mechanics; this can be seen in [67] and the references cited therein. The Coulomb potential has also offered many incredibly important applications in the density functional theory; see [34, 35, 65, 69]. In particular, the *indirect Coulomb energy* and its bounds have been obtained via the Lieb-Oxford inequality and the *n*-marginal optimal transport with Coulomb costs. When n=2, the Coulomb case coincides with the logarithmic case; this is also known as *log gas, 2-D one-component plasma, 2-D jellium, or Dyson gas* in physics. The 2-D one-component plasma is seen as a natural toy model for plasmas in 2-D statistical physics, the fractional quantum Hall effect, and random matrices.

We consider the Cauchy problem for (1.1) with the initial data $\rho _0: \mathbb {R}^n \rightarrow \mathbb {R}$ and $\mathcal {M}_0: \mathbb {R}^n \rightarrow \mathbb {R}^n$ that satisfy1.4Since the global solutions of CEREs can contain vacuum states $\{ (t,\boldsymbol{x}) : \, \rho (t,\boldsymbol{x}) = 0 \}$ where the fluid velocity is ill-defined, we use the momentum ${\mathcal {M}}(t,\boldsymbol{x})$, which will be shown to be well-defined globally, instead of the fluid velocity $u(t,\boldsymbol{x})$. Under these assumptions, the energy of CEREs is given by

$$\begin{aligned} \mathcal {E}(\rho ,\mathcal {M})(t): =\int _{\mathbb {R}^n}\Big (\rho (t, \boldsymbol{x}) e(\rho (t, \boldsymbol{x})) + \frac{1}{2} \Big |\frac{\mathcal {M}}{\sqrt{\rho }}\Big |^2(t, \boldsymbol{x}) + \frac{\kappa }{2}\rho (\boldsymbol{x})(\Phi _\alpha *\rho )(t, \boldsymbol{x})\Big )\,\ {\, \mathrm d}\boldsymbol{x}, \end{aligned}$$

where $e(\rho ) = \frac{a_0}{\gamma - 1}\rho ^{\gamma - 1}$ is the internal energy. Much work has been done on this in the Coulomb case or Newtonian potential (α=n-2), known as the compressible Euler-Poisson equations (CEPEs). In the case of plasma (κ=-1, the repulsive case), in a series of work by Guo [48], Guo-Pausader [53], Guo-Ionescu-Pausader [52], and Ionescu-Pausader [60], it was proved that there exist global smooth irrotational solutions close to some constant background with small amplitude. Meanwhile, for the gravitational gaseous star problem (κ=1, the attractive case), in Goldreich-Weber [47], a family of smooth compact polytropic collapsing star solutions of the 3-D CEPEs was found in the critical case of $\gamma = \frac{4}{3}$. It was then proved that such solutions are nonlinearly stable under a small radially symmetric perturbation in Hadzić-Jang [55]. It was also proved by Hadzić-Jang [56] in the case of both plasma and gaseous stars ($\kappa =\pm 1$) that there exists a family of expanding global-in-time solutions in Lagrangian coordinates to the 3-D CEPEs in the case that $\gamma = 1 + \frac{1}{k}$ for $k\in \mathbb {N}$.

However, since CEPEs are strongly hyperbolic and nonlinear, there is a breakdown of smooth solutions in finite time for large initial data, in general. The challenging behaviors we might encounter are the formation of shock waves causing discontinuities and the occurrence of the cavitation and concentration of the density, which are a major area of interest, still not fully understood. In particular, concentration of the density at the origin for spherically symmetric solutions exhibiting gravitational collapse can occur for $\gamma \in [1, \frac{4}{3})$ in the gaseous star case (*cf*. [49–51]). As such, we have to focus on the weak solutions of CEPEs. Indeed, it was proved by Chen-He-Wang-Yuan [26] that, for γ>1 in the repulsive case (κ=-1) and $\gamma > \frac{2n}{n + 2}$ with some conditions on the initial mass $M < M_\textrm{c}$ for $\gamma \in (\frac{2n}{n + 2}, \frac{2n - 2}{n})$ in the attractive case (κ=1), given the initial data of finite energy, there exist global finite-energy solutions of CEPEs for n≥3, which implies that no concentration for the density is formed at the origin.

The difficulty of studying the general case of CEREs is that the Riesz potential can no longer be represented by using a local formula, due to the non-locality of the potential. This provides us with multiple issues when trying to generalize from the Coulomb case to the general Riesz case. As such, the existence results for CEREs were only recently proved, starting with that of Choi-Jeong [31], where they proved that CEREs are locally well-posed away from the vacuum for the attractive and repulsive cases for $\alpha \in (n-2,n)$ and showed the finite-time blowup of classical solutions. Danchin-Ducomet [37] proved that, under the assumption that the initial density is small enough and the initial velocity is close to some vector field $v_0$ such that the spectrum of $Dv_0$ is positive and bounded away from zero, there exists a global classical solution for $\alpha \in [n-2,n-1]$. Choi-Jung [32] proved that, in the pressureless case, under the conditions that the initial data are small enough in some regular enough Sobolev space, there exists a unique global classical solution for $\alpha \in (n-2,n)$. Choi-Jung-Lee [33] extended the pressureless case to isentropic flows with pressure and proved that, under the condition that the initial density is small, the initial velocity is close enough to some reference velocity $v_0$ in some regular enough Sobolev space and, for a range of γ, there exists a unique global-in-time solution. We also refer to [8, 21, 59] for the critical value α=n-1, which enjoys similar physical phenomena with the Vlasov-Riesz system.

It is of fundamental physical interest to understand whether the solutions of CEREs are stable close to steady states of the equations. We observe that, for $\alpha \in (0,n)$, no steady states exist in the repulsive case κ=-1, since both the pressure and the nonlocal Riesz interaction are repulsive. Therefore, the study of steady states is confined to the attractive case κ=1. For a fixed α, such a steady state is usually given by a minimizer of the energy functional:

1.5 $$\begin{aligned} \mathcal {G}(\varrho ) =\int _{\mathbb {R}^n} \big (\varrho (\boldsymbol{x}) e(\varrho (\boldsymbol{x})) +\frac{1}{2}\varrho (\boldsymbol{x})(\Phi _\alpha *\varrho )(\boldsymbol{x})\big )\, {\, \mathrm d}\boldsymbol{x}, \end{aligned}$$

when it exists. Much work has been done towards this in the Coulomb case, that is, CEPEs. Firstly, Rein [80] proved that, under the condition that the minimizer is unique and a solution exists to the 3-D CEPEs with a condition on the pressure (which equates to $\gamma > \frac{2n - 2}{n}$ in the isentropic case) and is *close* to the minimizer at time t=0 in some sense, then the solution remains *close* to the unique minimizer for all time. This is known as a nonlinear stability result; in this case, we refer to the unique minimizer as nonlinearly stable. In Luo-Smoller [75, 76], they obtained the nonlinear stability results for the 3-D rotating Euler-Poisson equations and proved the existence of the solutions and minimizers. Recently, Lin-Zeng [70] established a turning point principle, which provides sharp linear stability and instability criteria for the non-rotating gaseous stars. Based on the linear stability criterion, Lin-Wang-Zhu [71] obtained nonlinear stability results for CEPEs with a general pressure law. We also refer to [41, 61, 62] for the nonlinear instability of the polytropic stars (n=3,α=n-2) for $\gamma \in [\frac{6}{5},\frac{4}{3}]$. It is well-known that the steady states of CEREs relate to the steady states of the aggregation-diffusion equation with a Riesz potential, which can be seen by the fact that the corresponding energy functional is identical. Carrillo-Hittmeir-Volzone-Yao [18] proved that stationary states of a large class of aggregation-diffusion equations must be radial. As a consequence, they proved that there exists a unique radially symmetric stationary state, that is the unique radial minimizer, to the Keller-Segel equations, a special case of the aggregation-diffusion equations, where the non-local potential is given by the Coulomb potential. We refer to [19] and the references therein for further properties of the aggregation-diffusion equations.

The occurrence of cavitation and the formation of shock waves can lead to difficulties with the global existence of problem (1.1)–(1.4), as well as a lack of regularity of the solutions. Hence, in order to prove the global existence of finite-energy solutions with spherical symmetry, we use a vanishing physical viscosity method through the approximate PDEs, called the compressible Navier–Stokes-Riesz equations (CNSREs), of the form:

1.6 $$\begin{aligned} {\left\{ \begin{array}{ll} \displaystyle \partial _t \rho +\nabla \cdot {\mathcal {M}}=0, \\ \displaystyle \partial _t {\mathcal {M}}+ \nabla \cdot \Big (\frac{{\mathcal {M}}\otimes {\mathcal {M}}}{\rho }\Big ) +\nabla p + \kappa \rho \nabla \mathcal {W}_\alpha = \varepsilon \nabla \cdot \Big (\mu (\rho )D \big (\frac{{\mathcal {M}}}{\rho } \big ) \Big ) + \varepsilon \nabla \Big ( \lambda (\rho ) \nabla \cdot \big (\frac{{\mathcal {M}}}{\rho } \big ) \Big ), \end{array}\right. } \end{aligned}$$

where $D\big ( \frac{{\mathcal {M}}}{\rho } \big ) = \frac{1}{2} \big ( \nabla (\frac{{\mathcal {M}}}{\rho }) + \big (\nabla ( \frac{{\mathcal {M}}}{\rho }) \big )^\intercal \big )$ is the stress tensor, ε>0 can be seen as the inverse of the Reynolds number, and $\mu (\rho )$ and $\lambda (\rho )$ are the shear and bulk viscosity coefficients respectively that satisfyHere the viscosity coefficients satisfy the BD relation: $\lambda (\rho ) = \rho \mu '(\rho ) - \mu (\rho )$, needed to derive a BD-type entropy estimate to gain information on the derivatives of the density; see Bresch-Desjardins [9] and Bresch-Desjardins-Lin [10]. We will choose $(\mu (\rho ),\lambda (\rho )) = (\rho ,0)$, which satisfies the required properties, for simplicity. Formally, one can see that, as $\varepsilon \rightarrow 0$, solutions of (1.6) converge to solutions of (1.1). However, the rigorous proof of this has remained open; see Chen-Feldman [25] and Dafermos [36] for the details. Significant progress had been made towards this in the Coulomb case α=n-2.

Since system (1.1) is an M-D system of hyperbolic balance laws, not much is understood about such equations. Such equations usually do not always admit classical solutions due to the formation of discontinuities and singularities. A more classical theory in the area was established by Glimm [46], which provides the global-in-time existence of entropy solutions of bounded variation for the 1-D general hyperbolic systems of conservation laws in the case of initial conditions close to some constant function. In Bianchini-Bressan [6], the existence of entropy solutions of bounded variation was established for the 1-D general hyperbolic systems of conservation laws via the artificial vanishing viscosity method. DiPerna [39] first proved the existence of entropy solutions for the isentropic Euler equations for polytropic gases with $\gamma =1+\frac{2}{2k+1}$, with integer k≥2, by developing the method of compensated compactness (*cf*. Murat [77] and Tartar [84]). Chen [24] and Ding-Chen-Luo [38] established the global existence of entropy solutions in $L^\infty$ for the general case $\gamma \in (1,\frac{5}{3}]$ by developing new techniques through careful entropy analysis that involves fractional derivatives, the Hilbert transform, and the compensated compactness argument. Then Lions-Perthame-Tadmor [74] proved the global existence result for entropy solutions in $L^\infty$ for $\gamma \in [3,\infty )$. The gap $\gamma \in (\frac{5}{3},3)$ between the ranges was then bridged by Lions-Perthame-Souganidis [73]. The existence result for entropy solutions in $L^\infty$ was then proved by Huang-Wang [58] for γ=1.

The idea of using the vanishing viscosity method to study solutions of hyperbolic problems goes back decades. Gilbarg [45] proved that, for the viscous approximation for 1-D heat-conducting fluids, there exists a unique shock layer that converges to a shock wave of the inviscid equations in the inviscid limit. Hoff-Liu [54] proved that the solutions of the 1-D Navier–Stokes equations for compressible isentropic flow converge to an entropy-satisfying shock-wave solution in the inviscid limit. Chen-Perepelitsa [27] first completed the proof of the inviscid limit rigorously of the solutions of the 1-D Navier–Stokes equations to the solutions of the isentropic Euler equations with general large initial data and relative finite-energy for all the range of γ>1. This is achieved by the compensated compactness framework in $L^p$, studied first by LeFloch-Westdickenberg [64] for $\gamma \in (1,\frac{5}{3})$ and later further developed by Chen-Perepelitsa [27] to handle all the adiabatic exponent γ>1 with a simplified proof. Such a compensated compactness framework has been further studied in [28–30, 81] to prove spherically symmetric solutions of the M-D isentropic Euler equations.

Our aim is to establish the existence of global finite-energy solutions of (1.1)–(1.4) for large spherically symmetric initial data. To this end, we consider the solutions of form:

1.7 $$\begin{aligned} \rho (t,\boldsymbol{x}) = \rho (t,r), \quad {\mathcal {M}}(t,\boldsymbol{x}) = m(t,r) \frac{\boldsymbol{x}}{r} \qquad \,\, \text { for} \quad r = |\boldsymbol{x}|, \end{aligned}$$

with initial conditions:

1.8 $$\begin{aligned} (\rho , {\mathcal {M}})|_{t=0} = (\rho _0, {\mathcal {M}}_0)(\boldsymbol{x}), \end{aligned}$$

where $\rho _0 \in L^1_+(\mathbb {R}^n)$ and $\frac{{\mathcal {M}}_0}{\sqrt{\rho _0}} \in L^2(\mathbb {R}^n)$. Under these assumptions, (1.1) can be written in the form:

$$\begin{aligned} {\left\{ \begin{array}{ll} \displaystyle \rho _t + m_r + \frac{n-1}{r}m = 0, \\ \displaystyle m_t + \Big (\frac{m^2}{\rho } + p(\rho )\Big )_r + \frac{n-1}{r}\frac{m^2}{\rho } + \kappa \rho \,(\mathcal {W}_\alpha )_r = 0. \end{array}\right. } \end{aligned}$$

Similarly, in the case of CNSREs, (1.6) becomes

1.9 $$\begin{aligned} {\left\{ \begin{array}{ll} \displaystyle \rho _t + m_r + \frac{n-1}{r}m = 0, \\ \displaystyle m_t + \Big (\frac{m^2}{\rho } + p(\rho )\Big )_r + \frac{n-1}{r}\frac{m^2}{\rho } + \kappa \rho \,(\mathcal {W}_\alpha )_r = \varepsilon \Big ( (\mu + \lambda ) \big ( (\frac{m}{\rho })_r + \frac{n-1}{r}\frac{m}{\rho } \big ) \Big )_r- \varepsilon \frac{n-1}{r}\mu _r\frac{m}{\rho }. \end{array}\right. } \end{aligned}$$

Using the form of spherically symmetric solutions allows us to reduce our equations from a system of n+1 equations for n+1 variables to a system of 2 equations with 2 variables. However, this system is still tricky to work with. For the Coulomb case (α=n-2), considerable work has been done toward the global existence of the compressible Navier–Stokes-Poisson equations (CNSPEs). It was proved by Duan-Li [42] that, for spherically symmetric compactly supported arbitrarily large initial data, there exist global weak spherically symmetric solutions of CNSPEs with a stress-free boundary condition and $\gamma \in (\frac{6}{5},\frac{4}{3}]$. An important result in Chen-He-Wang-Yuan [26] is the global existence of spherically symmetric solutions of CEPEs (*i*.*e*.,  the Coulomb case α=n-2 for n≥3). Different from [26], in this paper, we prove the global existence and nonlinear stability of finite-energy solutions for CEREs with a general class of Riesz potentials and the logarithmic potential for dimension n≥2.

One of the main difficulties we have to overcome has been mentioned above as the non-locality of the potential for CEREs. The potential for CEPEs with the Coulomb potential α=n-2 can be represented locally as an exact formula given by

$$ (\Phi _{n-2} * \rho )_r(t,r) = \frac{\omega _n}{r^{n-1}} \int ^r_0 \rho (t,\eta ) \eta ^{n-1} {\, \mathrm d}\eta , $$

where *r* is the radial coordinate and $\omega _n$ is the surface area of the *n*-D unit ball. This is fundamental in proving the uniform energy estimates, the BD-type entropy, and the higher integrability in [26]. Such a formula is derived from the fact that the Coulomb kernel satisfies the following Poisson equation:

$$ \Delta (\Phi _{n-2} * \rho ) = \omega _n \rho , $$

which is a local equation. However, for general α, we no longer have this type of structure, hence such a local formula is not possible. Even in the case $\alpha \in (n-2,n)$, the potential solves a fractional Poisson-type equation, which is a non-local equation and so cannot be employed to derive a local estimate. However, to overcome this problem, we use a radial representation for the Riesz potential to obtain the estimates on the potential (see Lemma 3.1), which is incredibly important in obtaining the estimates on the approximate solutions. This is particularly so in the case of higher integrability estimates, due to the non-locality of the potential, where the local estimates are required.

Moreover, when analyzing the convergence of the potential energies as solutions transit from the free boundary problem to the Cauchy problem of global weak solutions, the absence of the local representation formula for the potential terms necessitates an alternative approach to that of [26]. Specifically, to understand the behavior near the origin, we adopt a method distinct from the previous work. Although the earlier studies provided the local convergence of the density away from the origin in the limit, we establish a stronger result by proving the global convergence of the density in this paper; see Lemma 4.7. The key point stems from the combination of the local convergence and the mass conservation in the limit, which ensures that there is neither a blow-up of mass at the origin nor a loss of mass at infinity. These enable us to derive the global convergence result.

Other important technical novelties of our work concern the BD-type entropy estimates. When the Riesz potential is sub-Coulombic or Coulomb, *i.e.,* $\alpha \le n - 2$, we want to obtain a BD-type entropy estimate. However, due to the nonlocality of the potential, we discover that, when the integration by parts is applied, we obtain a nonzero boundary term only in the case that α<n-2, as observed in (3.46); see also Remark 3.10. In the repulsive case κ=-1, the boundary term has a favorable sign and so can be ignored. However, in the attractive case κ=1, the boundary term cannot be controlled. Thus, we use a Grönwall-type inequality to bypass the use of the integration by parts method that gives us the problematic boundary term, which allows us to obtain a BD-type entropy estimate; see Lemma 3.9. In the case of the super-Coulombic Riesz potentials when α>n-2, the potential is no longer twice differentiable, so it is difficult to apply the method of integration by parts of [26] to obtain the BD entropy estimate. In this paper, we are able to overcome such a problem by applying the same Grönwall-type inequality as before to obtain the estimate for not only the attractive case but also the repulsive case; see Lemma 3.11.

Finally, we deal with the potential for the case that $\alpha \in (-1,0]$, in particular the logarithmic potential α=0. This logarithmic case appears in many applications in the physical and mathematical biology literature. However, it has been very problematic when trying to resolve the well-posedness of not only CEREs in general, but also CEPEs in the 2-D case. Unlike the Riesz potential, there is no longer decay at infinity and, in the logarithmic case, the potential has no definitive sign. In the case $\alpha \in (0,n)$, we can employ the Hardy-Littlewood-Sobolev (HLS) inequality to derive the $L^p$ estimate for $\Phi _\alpha * \rho$. The parallel in the logarithmic case α=0 is that of the logarithmic Hardy-Littlewood-Sobolev (logHLS) inequality, where we only obtain a one-sided bound and which no longer gives us an $L^p$ bound. In the case $\alpha \in (-1,0)$, we have the reverse HLS inequality that gives a lower bound estimate for the convolution in terms of an $L^q$ bound for q∈(0,1). Such difficulties have made the study of CEREs indubitably difficult. However, by studying the behavior of the logarithmic potential around the origin and at the far-field separately, we are able to locally control the $L^\infty$ norm of $\Phi _0 * \rho$, allowing us to overcome the previous difficulties that arose by the use of the logHLS inequality only. It is also shown that the energy estimates for the logarithmic potential can, at least formally, be seen to be a limit of the energy estimates for the solutions of the general Riesz potentials as $\alpha \rightarrow 0$, showing the connection between the Riesz and logarithmic potentials; see Remark 3.5.

We note that the previous results were focused on proving the conditional stability for radial perturbations of stationary solutions that rely on the pre-assumed global existence of weak solutions. One of our novelties here is that we can employ the first part of this paper to show that the assumption of the global existence of finite-energy solutions close to such stationary solutions is non-empty for the whole family of CEREs. The nonlinear stability of stationary solutions for CEPEs has been analyzed in [71, 75, 76, 80]. Notice further that our results for CEREs are *unconditional* now, by using the global existence results obtained in this paper (see also [26]). Moreover, we obtain the properties of steady states by variational arguments and building the connection with the aggregation-diffusion equations; see Remark 2.8 and Theorem 5.5.

The main strategy and structure of the paper are as follows: In §2, we present the main results of this paper. In §3, we first derive the local estimates for the radial representation of the Riesz potential and its derivatives. Subsequently, we prove the uniform energy estimates of solutions of the approximate free boundary problem to obtain the uniform $L^p$ estimates. The next step is to prove a BD-type inequality to derive the uniform $L^p$ estimates on the derivative of the density. Finally, we use the local estimates for the Riesz potential to prove the uniform local higher-integrability estimates for the density and the velocity. In §4, we prove the main results concerning the global finite-energy solutions. We also apply the Aubin-Lions lemma via the use of the BD-type estimates to pass from the solutions of the free boundary problem to the global weak solutions of CNSREs, which satisfy the same uniform estimates. We also employ the higher integrability estimates in order to use the compensated compactness argument from Chen-Perepelitsa [27] to pass from the global weak solutions of CNSREs to the global weak solutions of CEREs. Finally, in §5, we develop a simplified variational approach to deal with the nonlinear stability. We employ the concentration compactness argument from Lions [72] to prove the existence of minimizers of (1.5) and the nonlinear stability near such minimizers. The appendices contain details on the convolution inequalities used in the main text and the construction of the approximate initial data.

## Mathematical Problems and Construction of Approximate Solutions

In this section, we first introduce the notion of weak solutions and steady states of CEREs and CNSREs, then describe the main results of this paper, and finally present the construction of approximate solutions to obtain the global weak solutions.

### Weak solutions and steady states of CEREs and CNSREs

We first assume that the given initial data function $(\rho _0,{\mathcal {M}}_0)(\boldsymbol{x})$ has finite total energy and total mass:

2.1 $$\begin{aligned}&E_0 {:=}\int _{\mathbb {R}^n}\rho _0 \Big ( \frac{1}{2} \Big | \frac{{\mathcal {M}}_0}{\rho _0}\Big |^2 + e(\rho _0) + \frac{\kappa }{2} (\Phi _\alpha * \rho _0) \Big )(\boldsymbol{x}) {\, \mathrm d}\boldsymbol{x} < \infty , \end{aligned}$$

2.2 $$\begin{aligned}&M {:=}\int _{\mathbb {R}^n} \rho _0(\boldsymbol{x}) {\, \mathrm d}\boldsymbol{x} = \omega _n \int ^\infty _0 \rho _0(r) r^{n-1} {\, \mathrm d}r < \infty . \end{aligned}$$

When $\alpha \in (-1,0]$, we also assume that $(\rho _0,{\mathcal {M}}_0)(\boldsymbol{x})$ has finite (-α)-moment:

2.3 $$\begin{aligned} \int _{\mathbb {R}^n} \rho _0(\boldsymbol{x}) k_\alpha (1 + |\boldsymbol{x}|^2) {\, \mathrm d}\boldsymbol{x} = \omega _n \int ^\infty _0 \rho _0(r) k_\alpha (1 + r^2) r^{n-1} {\, \mathrm d}r < \infty , \end{aligned}$$

where

$$\begin{aligned} k_\alpha (r) = {\left\{ \begin{array}{ll} r^{- \frac{\alpha }{2}} \quad & \text {for } \alpha \in (-1,0), \\ \log (r) & \text {for } \alpha = 0, \end{array}\right. } \end{aligned}$$

$\omega _n$ represents the surface area of the unit sphere in $\mathbb {R}^n$, and $e(\rho ) = \frac{a_0}{\gamma - 1}\rho ^{\gamma - 1}$ is the internal energy satisfying the relation: $p(\rho ) = \rho ^2e'(\rho )$. Here we require the initial data such that the convolution $\Phi _\alpha *\rho _0$ is well-defined and differentiable. We now give the definition of global finite-energy weak solutions of (1.1)–(1.4).

#### Definition 2.1

A measurable vector function $(\rho ,{\mathcal {M}})(t, \boldsymbol{x})$ is a finite-energy solution of the Cauchy problem (1.1)–(1.4) if $(\rho ,{\mathcal {M}})(t, \boldsymbol{x})$ satisfies the following conditions:

- $\rho (t,\boldsymbol{x}) \ge 0$ *a.e.* and $({\mathcal {M}}, \frac{{\mathcal {M}}}{\sqrt{\rho }})(t,\boldsymbol{x}) = \boldsymbol{0}$ *a.e.* on the vacuum states $\{ (t,\boldsymbol{x}) : \, \rho (t,\boldsymbol{x}) = 0 \}$,
  $$\begin{aligned}&\rho \in L^{\infty }(0,T; L^1(\mathbb {R}^n)\cap L^\gamma (\mathbb {R}^n)), \quad \,\,\, \frac{{\mathcal {M}}}{\sqrt{\rho }}\in L^{\infty }(0,T; L^2(\mathbb {R}^n)). \end{aligned} $$
  Moreover, for $$\alpha \in (0,n-1)$$,
  $$ \Phi _\alpha * \rho \in L^\infty (0,T;L^{\frac{2n}{\alpha }}(\mathbb {R}^n) ),\quad \nabla \Phi _\alpha * \rho \in L^\infty (0,T;L^{\frac{2n}{\alpha + 2}}(\mathbb {R}^n)), $$
  and, for $$\alpha \in (-1,0]$$,
  $$ \Phi _\alpha * \rho \in L^\infty (0,T;L^\infty _\textrm{loc}(\mathbb {R}^n) ), \quad \nabla \Phi _\alpha * \rho \in L^\infty (0,T;L_\textrm{loc}^{\frac{2n}{\alpha + 2}}(\mathbb {R}^n)), $$
  for any T>0.
- For *a.e.* t>0, the total energy is finite:
  For κ=-1,
    $$\begin{aligned} \int _{\mathbb {R}^n} \Big ( \frac{1}{2} \Big | \frac{{\mathcal {M}}}{\sqrt{\rho }}\Big |^2 + \rho e(\rho ) - \frac{1}{2} \rho (\Phi _\alpha * \rho ) \Big )(t,\boldsymbol{x}) {\, \mathrm d}\boldsymbol{x} \le E_0. \end{aligned}$$
  For κ=1,
    $$\begin{aligned} {\left\{ \begin{array}{ll} \displaystyle \int _{\mathbb {R}^n} \Big ( \frac{1}{2} \Big | \frac{{\mathcal {M}}}{\sqrt{\rho }}\Big |^2 + \rho e(\rho ) - \frac{1}{2} \rho (\Phi _\alpha * \rho ) \Big )(t,\boldsymbol{x}) {\, \mathrm d}\boldsymbol{x} \le C(E_0,M), \\ \displaystyle \int _{\mathbb {R}^n} \Big ( \frac{1}{2}\Big |\frac{{\mathcal {M}}}{\sqrt{\rho }}\Big |^2 + \rho e(\rho ) + \frac{1}{2} \rho (\Phi _\alpha * \rho ) \Big )(t,\boldsymbol{x}) {\, \mathrm d}\boldsymbol{x} \le E_0. \end{array}\right. } \end{aligned}$$
- For any $$\zeta \in C^1_0(\mathbb {R}_{+} \times \mathbb {R}^n)$$,
  $$\begin{aligned} \int _{\mathbb {R}_+} \int _{\mathbb {R}^n} \big (\rho \zeta _t + {\mathcal {M}}\cdot \nabla \zeta \big ) {\, \mathrm d}\boldsymbol{x} {\, \mathrm d}t + \int _{\mathbb {R}^n} \rho _0(\boldsymbol{x}) \zeta (0,\boldsymbol{x}) {\, \mathrm d}\boldsymbol{x} = 0. \end{aligned}$$
- For any $$\boldsymbol{\psi } \in (C^1_0(\mathbb {R}_{+} \times \mathbb {R}^n))^n$$,
  $$\begin{aligned}&\int _{\mathbb {R}_+} \int _{\mathbb {R}^n} \Big ({\mathcal {M}}\cdot \boldsymbol{\psi }_t + \frac{{\mathcal {M}}}{\sqrt{\rho }} \cdot \big ( \frac{{\mathcal {M}}}{\sqrt{\rho }} \cdot \nabla \big ) \boldsymbol{\psi } + p(\rho )\nabla \cdot \boldsymbol{\psi } \Big )(t,\boldsymbol{x}) {\, \mathrm d}\boldsymbol{x} {\, \mathrm d}t \\&\qquad + \int _{\mathbb {R}^n} {\mathcal {M}}_0(\boldsymbol{x}) \cdot \boldsymbol{\psi }(0,\boldsymbol{x}) {\, \mathrm d}\boldsymbol{x} \\&\quad = \int _{\mathbb {R}_+} \int _{\mathbb {R}^n} \kappa (\rho \nabla \mathcal {W}_\alpha \cdot \boldsymbol{\psi })(t,\boldsymbol{x}) {\, \mathrm d}\boldsymbol{x} {\, \mathrm d}t. \end{aligned}$$

#### Remark 2.2

The reason for distinguishing between the attractive case κ=1 and the repulsive case κ=-1 lies in the fact that, in the attractive regime, the interaction energy enters with a negative sign. Nevertheless, the second energy condition in the attractive case ensures that the total energy is non-increasing. Combined with the first condition, this implies that the kinetic, internal, and potential energies are each individually bounded.

We first construct global weak solutions of CNSREs with appropriately adapted density-dependent viscosity terms and approximate initial conditions, constructed in Appendix B with the properties as in Lemmas B.1–B.8, given by

2.4 $$\begin{aligned} (\rho ^\varepsilon , {\mathcal {M}}^\varepsilon )(0,\boldsymbol{x}) = (\rho ^\varepsilon _0, {\mathcal {M}}^\varepsilon _0)(\boldsymbol{x}). \end{aligned}$$

Now we provide the definition of global weak solutions of the Cauchy problem (1.6) and (2.4).

#### Definition 2.3

A measurable vector function $(\rho ^\varepsilon ,{\mathcal {M}}^\varepsilon )(t,\boldsymbol{x})$ is a weak solution of the Cauchy problem (1.6) and (2.4) with $(\mu ,\lambda ) = (\rho ,0)$ if $(\rho ^\varepsilon ,{\mathcal {M}}^\varepsilon )(t,\boldsymbol{x})$ satisfies the following conditions:

- $\rho ^\varepsilon (t,\boldsymbol{x}) \ge 0$ *a.e.* and $({\mathcal {M}}^\varepsilon , \frac{{\mathcal {M}}^\varepsilon }{\sqrt{\rho ^\varepsilon }})(t,\boldsymbol{x}) = \boldsymbol{0}$ *a.e.* on the vacuum states $\{ (t,\boldsymbol{x}) : \, \rho ^\varepsilon (t,\boldsymbol{x}) = 0 \}$,
  $$\begin{aligned}&\rho ^\varepsilon \in L^{\infty }(0,T; L^1(\mathbb {R}^n)\cap L^\gamma (\mathbb {R}^n)), \quad \nabla \sqrt{\rho ^\varepsilon }\in L^{\infty }(0,T; L^2(\mathbb {R}^n)),\\&\frac{{\mathcal {M}}^\varepsilon }{\sqrt{\rho ^\varepsilon }}\in L^{\infty }(0,T; L^2(\mathbb {R}^n)). \end{aligned}$$
  Moreover, for $$\alpha \in (0,n-1)$$,
  $$ \Phi _\alpha * \rho ^\varepsilon \in L^\infty (0,T;L^{\frac{2n}{\alpha }}(\mathbb {R}^n) ),\quad \nabla \Phi _\alpha * \rho ^\varepsilon \in L^\infty (0,T;L^{\frac{2n}{\alpha + 2}}(\mathbb {R}^n)), $$
  and, for $$\alpha \in (-1,0]$$,
  $$ \Phi _\alpha * \rho ^\varepsilon \in L^\infty (0,T;L^\infty _\textrm{loc}(\mathbb {R}^n) ),\quad \nabla \Phi _\alpha * \rho ^\varepsilon \in L^\infty (0,T;L_\textrm{loc}^{\frac{2n}{\alpha + 2}}(\mathbb {R}^n)), $$
  for any T>0.
- For any $\zeta \in C^1_0(\mathbb {R}_+ \times \mathbb {R}^n)$ and $t_2 \ge t_1 \ge 0$,
  $$\begin{aligned} \int _{t_1}^{t_2} \int _{\mathbb {R}^n} (\rho ^\varepsilon \zeta _t + {\mathcal {M}}^\varepsilon \cdot \nabla \zeta ) {\, \mathrm d}\boldsymbol{x} {\, \mathrm d}t = \int _{\mathbb {R}^n} (\rho ^\varepsilon \zeta )(t_2,\boldsymbol{x}) {\, \mathrm d}\boldsymbol{x} - \int _{\mathbb {R}^n} (\rho ^\varepsilon \zeta )(t_1,\boldsymbol{x}) {\, \mathrm d}\boldsymbol{x}. \end{aligned}$$
- For any $$\boldsymbol{\psi } \in (C^1_0(\mathbb {R}_+ \times \mathbb {R}^n))^n$$,

$$\begin{aligned} \begin{aligned}&\int _{\mathbb {R}_+} \int _{\mathbb {R}^n} \Big ( {\mathcal {M}}^\varepsilon \cdot \boldsymbol{\psi }_t + \frac{{\mathcal {M}}^\varepsilon }{\sqrt{\rho ^\varepsilon }} \cdot \big ( \frac{{\mathcal {M}}^\varepsilon }{\sqrt{\rho ^\varepsilon }} \cdot \nabla \big ) \boldsymbol{\psi } + p(\rho ^\varepsilon )\nabla \cdot \boldsymbol{\psi } \Big )(t,\boldsymbol{x}) {\, \mathrm d}\boldsymbol{x} {\, \mathrm d}t \\&\qquad + \int _{\mathbb {R}^n} {\mathcal {M}}^\varepsilon _0(\boldsymbol{x}) \cdot \boldsymbol{\psi }(0,\boldsymbol{x}){\, \mathrm d}\boldsymbol{x} \\&\quad = - \varepsilon \int _{\mathbb {R}_+} \int _{\mathbb {R}^n} \Big ( \frac{1}{2} {\mathcal {M}}^\varepsilon \cdot \big ( \Delta \boldsymbol{\psi } + \nabla (\nabla \cdot \boldsymbol{\psi } ) \big ) \\&\qquad \qquad \qquad \qquad \quad + \frac{{\mathcal {M}}^\varepsilon }{\sqrt{\rho ^\varepsilon }} \cdot (\nabla \sqrt{\rho ^\varepsilon } \cdot \nabla ) \boldsymbol{\psi } + \nabla \sqrt{\rho ^\varepsilon } \cdot \big (\frac{{\mathcal {M}}^\varepsilon }{\sqrt{\rho ^\varepsilon }} \cdot \nabla \big )\boldsymbol{\psi }\Big ) {\, \mathrm d}\boldsymbol{x} {\, \mathrm d}t \\&\qquad + \int _{\mathbb {R}_+} \int _{\mathbb {R}^n} \kappa (\rho ^\varepsilon \nabla \mathcal {W}^\varepsilon _\alpha \cdot \boldsymbol{\psi })(t,\boldsymbol{x}) {\, \mathrm d}\boldsymbol{x} {\, \mathrm d}t, \end{aligned} \end{aligned}$$

where

$$ \nabla \mathcal {W}^\varepsilon _\alpha (\boldsymbol{x}) = {\left\{ \begin{array}{ll} \displaystyle (\nabla \Phi _\alpha * \rho ^\varepsilon ) (\boldsymbol{x}) &  \text {for } \alpha \in (-1,n-1), \\ \displaystyle \int _{\mathbb {R}^n} \nabla \Phi _\alpha (\boldsymbol{x} - \boldsymbol{y}) \big (\rho ^\varepsilon (\boldsymbol{y}) - \rho ^\varepsilon (\boldsymbol{x})\big ) {\, \mathrm d}\boldsymbol{y} \quad &  \text {for } \alpha \in [n-1,n). \end{array}\right. } $$

One of the important results of this paper is the nonlinear stability of the unique minimizer of the energy functional relating to the attractive CEREs. For $\alpha \in (0,n)$ and 0<M<∞, define the set of admissible functions:

2.5 $$\begin{aligned} X_M {:=}\Big \{ \varrho \in L^1_+(\mathbb {R}^n) \cap L^\frac{n + \alpha }{n}(\mathbb {R}^n) \, : \, \int _{\mathbb {R}^n} \varrho (\boldsymbol{x}) \, {\, \mathrm d}\boldsymbol{x} = M \Big \}. \end{aligned}$$

For $\varrho \in X_M$, define the energy functional $\mathcal {G}$:

2.6 $$\begin{aligned} \mathcal {G}(\varrho )=\int _{\mathbb {R}^n} \Big (\varrho (\boldsymbol{x}) e(\varrho (\boldsymbol{x}))+\frac{1}{2}\varrho (\boldsymbol{x})(\Phi _\alpha *\varrho )(\boldsymbol{x})\Big )\,{\, \mathrm d}\boldsymbol{x}. \end{aligned}$$

For $\varrho , \tilde{\rho } \in X_M,$ define the distance:

2.7 $$\begin{aligned} d(\varrho ,\tilde{\rho }) =\int _{\mathbb {R}^n}\big ((\varrho e(\varrho )-\tilde{\rho }e(\tilde{\rho })) +(\Phi _\alpha *\tilde{\rho })(\varrho -\tilde{\rho })\big )\,{\, \mathrm d}\boldsymbol{x}. \end{aligned}$$

Moreover, it can be shown that, in some cases, the unique minimizer of the energy functional $\mathcal {G}$ in $X_M$ exactly corresponds to the unique steady state of (1.1), that is,

$$\begin{aligned} \nabla p + \kappa \varrho \nabla \mathcal {W}_\alpha = \boldsymbol{0}; \end{aligned}$$

see §5 for more details.

Then this, paired with a nonlinear stability result for the minimizer of $\mathcal {G}$, allows us to show that the steady state is nonlinearly stable.

#### Definition 2.4

$\tilde{\rho } \in L^1_+(\mathbb {R}^n) \cap L^\infty (\mathbb {R}^n)$ is called a steady state of (1.1) for κ=1 if $\tilde{\rho }^\gamma \in {W}^{1,2}_\text {loc}(\mathbb {R}^n)$ and $\mathcal {\tilde{W}}_\alpha =\Phi _\alpha *\tilde{\rho }$ with $\nabla \mathcal {\tilde{W}}_\alpha \in L^1_\text {loc}(\mathbb {R}^n)$, and

$$ \nabla p(\tilde{\rho }) + \kappa \tilde{\rho } \nabla \mathcal {\tilde{W}}_\alpha = \boldsymbol{0} $$

is satisfied in the sense of distributions; in addition, when $\alpha \in [n-1,n)$, $\tilde{\rho } \in C^{0,\beta }(\mathbb {R}^n)$ for some $\beta \in (1 + \alpha - n,1)$.

Notice that the notion of being a steady state of (1.1) is equivalent to the notion of being a steady state of the aggregation-diffusion equation

$$ \frac{\partial \rho }{\partial t}=\nabla \cdot \big (\nabla p(\rho ) + \kappa \rho \nabla \mathcal {W}_\alpha \big ); $$

see for instance [18, 19]. We will summarize the state-of-the-art for steady states in Theorem 5.5 in §5.

In the case that, for any fixed α, there exists a unique steady solution $\rho _s$ modulo mass normalization and translations; then $\rho _s$ is referred to as the Lane-Emden-Fowler type solution. A detailed discussion on the case of the existence and uniqueness modulo symmetries is summarized in Theorem 5.5 and postponed to §5. The steady state $\rho _s(|\boldsymbol{x}|)$ has compact support and is determined by the equation:

$$\begin{aligned} \nabla p_s + \kappa \rho _s\,\nabla \Phi _{\alpha } * \rho _s=\boldsymbol{0}, \end{aligned}$$

where $p_s(|\boldsymbol{x}|)=a_0(\rho _s(|\boldsymbol{x}|))^{\frac{n + \alpha }{n}}$. In particular, for α=n-2, the steady solution is the Lane-Emden solution.

### Main results

We first have the following global existence theorem:

#### Theorem 2.5

(Existence of Spherically Symmetric Solutions of CEREs). Consider problem (1.1)–(1.4) with $\alpha \in (-1,n-1)$ and the initial data of spherical symmetry as defined in (1.7)–(1.8). Assume that $(\rho _0,{\mathcal {M}}_0)$ satisfy (2.1)–(2.2). In addition, when $\alpha \in (-1,0],$ assume that (2.3) also holds. Assume further that $\gamma > \frac{1}{n-1-\alpha }$, and

- When κ=-1, $\,\,\rho _0 \in L^{\frac{2n}{2n - \alpha }}(\mathbb {R}^n)$ for $\alpha \in [0,n-1)$ and
  - γ>1 for $$\alpha \in (-1,n-2]$$,
  - $$\gamma \ge \frac{3n}{3n - 2(1+\alpha )}$$ for $$\alpha \in (n-2,n-1)$$;
- When κ=1, $\,\,\gamma \ge \frac{3n}{3n - 2(1+\alpha )}$ when $\alpha \ne n-2$ and
  (c) γ>1 for $$\alpha \in (-1,0]$$,
  (d) $$\gamma > \frac{n + \alpha }{n}$$ for $$\alpha \in (0,n-1)$$,
  (e) $$M < M_\textrm{c}^\alpha (\gamma )$$ for $$\gamma \in (\frac{2n}{2n - \alpha }, \frac{n + \alpha }{n}]$$ and $$\alpha \in (0,n-1)$$.

Then there exists a global spherically symmetric finite-energy solution $(\rho ,{\mathcal {M}})(t,\boldsymbol{x})$ of problem (1.1)–(1.4) in the sense of Definition 2.1.

In Theorem 2.5, $M_\textrm{c}^\alpha (\frac{n + \alpha }{n})$ for $\alpha \in (0,n)$ is the sharp critical mass, analogous to that of the Chandrasekhar limit mass $M_\textrm{ch}:=M_\textrm{c}^{n-2}(\frac{2(n-1)}{n})$ for steady gaseous stars [23]. It is known that there is no steady white dwarf star of the total mass $M > M_\textrm{ch}$; that is, there is no steady solution $(\rho _s(|\boldsymbol{x}|),\boldsymbol{0})$ for $p(\rho )=a_0\rho ^{\frac{2n - 2}{n}}$ for this case. The analogous dichotomy and the sharp critical mass for all $\alpha \in (0,n)$ were obtained in [17] in relation to variants of the HLS inequalities; see [7] for the Coulomb potential case α=n-2.

The critical mass for α>0 and $\gamma \in (\frac{2n}{2n-\alpha }, \frac{n + \alpha }{n}]$ is defined by

$$\begin{aligned} M^{\alpha }_\textrm{c}(\gamma ) = {\left\{ \begin{array}{ll} \displaystyle B_{n,\gamma ,\alpha }^{-\frac{n}{n - \alpha }} &  \text { when } \gamma = \frac{n + \alpha }{n},\\ \displaystyle \Big (\frac{\alpha B_{n,\gamma ,\alpha }}{n(\gamma - 1)} \Big )^{\frac{n(\gamma - 1)}{2n - \gamma (2n-\alpha )}} \Big (\frac{\alpha E_0}{\alpha - n(\gamma - 1)}\Big )^{\frac{\alpha - n(\gamma - 1)}{2n - \gamma (2n - \alpha )}} \quad &  \text { when } \gamma \in (\frac{2n}{2n - \alpha }, \frac{n + \alpha }{n}), \end{array}\right. } \end{aligned}$$

where

$$\begin{aligned} B_{n,\gamma ,\alpha } = \frac{C_*(\alpha ,\gamma ,n)}{2\alpha }\omega _n^{\frac{\alpha }{n(\gamma - 1)}-1} \Big (\frac{\gamma - 1}{a_0}\Big )^{\frac{\alpha }{n(\gamma - 1)}}, \end{aligned}$$

and $C_*(\alpha ,\gamma ,n) > 0$ is the sharp constant given by the variation of the HLS inequality from Lemma A.3. For α=n-2, the bound on the critical mass $M_\textrm{c}^{n-2}(\gamma )=B_{n,\gamma ,n-2}^{-\frac{n}{2}}$ for $\gamma <\frac{2(n-1)}{n}$ was obtained in [26].

#### Remark 2.6

**Fig. 1:**
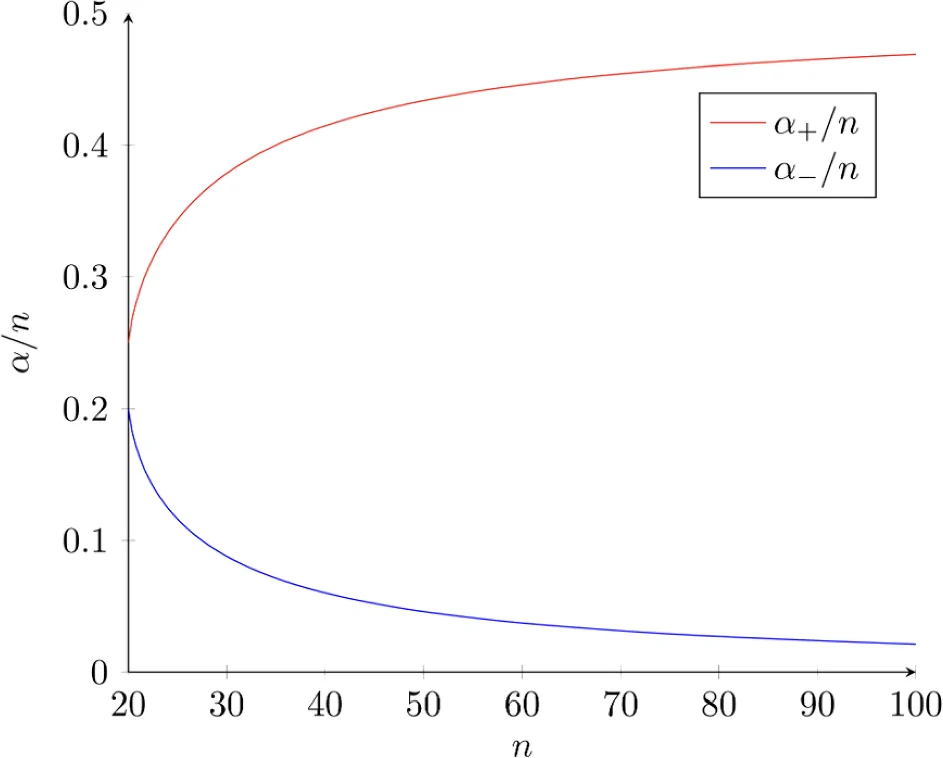
Relation between α and *n*

For Theorem 2.5, we have the following comments:

(i) In the attractive regime for $\gamma \in (\frac{2n}{2n-\alpha },\frac{n+\alpha }{n}]$, the condition $M < M_\textrm{c}$ is required to ensure that the potential energy can be controlled by the internal energy, thereby excluding finite-time blow-up of solutions. Such blow-up behavior has been shown to occur in [47, 55] for α=n-2, as discussed in the introduction.
(ii) The condition α<n-1 arises from several factors. First, we require this to obtain the higher integrability estimates in §3.5. This requirement stems from our use of a variant of the Hardy–Littlewood–Sobolev (HLS) inequality to derive the pointwise estimates for the radial representation of the potential. In addition, we explicitly rely on the HLS inequality to control the behavior of $\nabla \Phi _\alpha * \rho$, which is no longer feasible when this quantity is described by operator (1.2) in the regime $\alpha \ge n-1$. Extending the existence theory to this critical and more singular regime remains an interesting direction for future work.
(iii) For the repulsive case κ=-1 in the super-Coulomb regime $\alpha \in (n-2,n-1)$, and the attractive case κ=1 with $\alpha \ne n-2$, we impose the condition $\gamma \ge \frac{3n}{3n - 2(1+\alpha )}$. This requirement is of a technical nature and is needed to overcome difficulties that arise in the classical approach to establishing a BD-entropy estimate, which relies on integration by parts to control the potential term. In the classical approach, while in the super-Coulomb regime $\alpha \in (n-2, n-1)$ the lack of sufficient regularity prevents a direct use of integration by parts. These issues are discussed in more detail in the introduction and in §3.4. Given the technical nature of this assumption, we expect that solutions should exist for all $\gamma > \frac{2n}{2n-\alpha }$. In the critical case α=n-2, this additional condition is not required, yielding equivalent results known in previous literature for CEPEs. Moreover, we observe that $\frac{2n}{2n - \alpha } < \frac{3n}{3n - 2(1+\alpha )}$ for all $\alpha \in (-1,n-1)$, and the inequality $\frac{n + \alpha }{n} > \frac{3n}{3n - 2(1+\alpha )}$ holds only for certain values of α when n≥20. More precisely, this occurs for $\alpha \in (\alpha _-,\alpha _+)$, where $\alpha _{\pm } = \frac{n-2 \pm \sqrt{n^2 - 20n + 4}}{4}$ with $\frac{\alpha _-}{n}\rightarrow 0$ and $\frac{\alpha _+}{n} \rightarrow \frac{1}{2}$ as $n \rightarrow \infty$. Consequently, assumption (e) of Theorem 2.5 is relevant only for n≥20 and $\alpha \in (\alpha _-,\alpha _+)$, as illustrated in Fig. 1.
(iv) We summarize the conditions on γ in Theorem 2.5, together with simplified explanations for each case, in Table 1.

**Table 1:** Conditions on γ and *M* in Theorem 2.5

Condition	Ranges	Requisite for
$\gamma \ge \frac{3n}{3n-2(1+\alpha )}$	κ=-1 with $\alpha \in (n-2,n-1)$ or κ=1 with $\alpha \ne n-2$	Alternative BD-entropy method in §3.4.
$\gamma > \frac{1}{n-1-\alpha }$	$\alpha \in (n-2,n-1)$	Higher integrability estimates in §3.5.
$\gamma > \frac{2n}{2n - \alpha }$	κ=1 and $\alpha \in (0,n-1)$	Control of the potential energy by the HLS inequality in §3.2.
$M < M_\textrm{c}^\alpha (\gamma )$	κ=1, $\alpha \in (0,n-1)$, and $\gamma \in (\frac{2n}{2n - \alpha }, \frac{n + \alpha }{n}]$	Control of the potential energy by the internal energy in §3.2.

The nonlinear stability theorem is as follows:

#### Theorem 2.7

(Nonlinear Stability of Steady States for CEREs). Suppose that $\tilde{\rho }$ is a unique minimizer (up to translation) of the functional $\mathcal {G}$ in $X_M$. Let $\alpha \in (0,n - 1)$ with $\gamma > \frac{n + \alpha }{n}$. Let $(\rho ,\mathcal {M})(t,\boldsymbol{x})$ be global finite-energy solutions of the attractive CEREs (κ=1) in the sense of Definition 2.1 satisfying

$$ \int _{\mathbb {R}^n} \rho _0(\boldsymbol{x})\,{\, \mathrm d}\boldsymbol{x} =\int _{\mathbb {R}^n} \tilde{\rho }(\boldsymbol{x})\,{\, \mathrm d}\boldsymbol{x}=M. $$

Then, for any ε>0, there exists $\delta = \delta (\varepsilon )>0$ such that, if

2.8 $$\begin{aligned} d(\rho _0,\tilde{\rho })+ \Vert \rho _0 - \tilde{\rho } \Vert _{L^\frac{2n}{2n - \alpha }}^2 +\frac{1}{2}\int _{\mathbb {R}^n} \Big |\frac{\mathcal {M}_0}{\sqrt{\rho _0}}\Big |^2\,{\, \mathrm d}\boldsymbol{x}<\delta , \end{aligned}$$

there exists a translation $\boldsymbol{y}\in \mathbb {R}^n$ such that

2.9 $$\begin{aligned} d(\rho (t),T^{\boldsymbol{y}}\tilde{\rho }) + \Vert \rho (t)- T^{\boldsymbol{y}}\tilde{\rho } \Vert _{L^\frac{2n}{2n - \alpha }}^2+\frac{1}{2}\int _{\mathbb {R}^n} \Big |\frac{\mathcal {M}}{\sqrt{\rho }}\Big |^2(t)\,{\, \mathrm d}\boldsymbol{x}<\varepsilon \qquad \text {for a.e. } t > 0 , \end{aligned}$$

where $T^{\boldsymbol{y}}\tilde{\rho }(\boldsymbol{x})=\tilde{\rho }(\boldsymbol{x}+\boldsymbol{y})$ and, for a.e. t>0, $\rho (t):=\rho (t,\cdot )\in X_M$ for the density $\rho (t,\boldsymbol{x})$ of the global finite-energy solution.

#### Remark 2.8

For Theorem 2.7, we have the following comments:

i) The proof follows the same strategy of [80] for CEPEs. Concerning the existence proof of the minimizers of the free energy with homogeneous interaction potentials, different from Luo-Smoller [75, 76], we develop a simplified approach by employing the work of [72], as it was done in [17]. These methods may be further developed to handle the general pressure law case and other related problems, which will be explored elsewhere.
ii) We summarize the properties of global minimizers and the uniqueness of steady states in Theorem 5.5. As already mentioned, steady states of aggregation-diffusion equations are equivalent to steady states of CEREs or CEPEs. The results we have obtained cover a wider range of potentials than the previous results for CEREs and CEPEs, in particular, including n=2.

### Approximate PDEs

Some of the main difficulties we face for problem (1.1)–(1.4) and (1.6) in the whole space $\mathbb {R}^n$ are that the solutions may involve cavitation, concentration, and shock waves. In order to avoid these difficulties, we propose the following formulation of a free boundary problem for our approximate solutions:

2.10 $$\begin{aligned} {\left\{ \begin{array}{ll} \displaystyle \rho _t + (\rho u)_r + \frac{n-1}{r}\rho u = 0, \\ \displaystyle (\rho u )_t + (\rho u^2 + p(\rho ))_r + \frac{n-1}{r}\rho u^2 + \kappa \rho (\Phi _\alpha * \rho )_r = \varepsilon \Big ( \rho \big (u_r + \frac{n-1}{r}u \big ) \Big )_r - \varepsilon \frac{n-1}{r}\rho _ru. \end{array}\right. } \end{aligned}$$

This is defined on the moving domain:

$$ \Omega ^T = \big \{ (t,r) \,:\, 0 \le t \le T, \, a \le r \le b(t)\big \}, $$

where *b*(*t*) is a free boundary that is subject to:

2.11 $$\begin{aligned} {\left\{ \begin{array}{ll} b' (t) = u(t,b(t)) &  \text { for}\; t>0, \\ b(0) = b, \end{array}\right. } \end{aligned}$$

with boundary conditions:

2.12 $$\begin{aligned} u(t,a) = 0, \quad \big (p(\rho )-\varepsilon \rho (u_r + \frac{n-1}{r}u) \big )(t,b(t)) = 0 \qquad \,\,\, \text {for}\; t >0, \end{aligned}$$

for $$a:= b^{-1}$$ with b≫1.

The initial conditions are prescribed by

2.13 $$\begin{aligned} (\rho ,u)|_{t=0}(r) = (\rho _0^{\varepsilon ,b},u_0^{\varepsilon ,b})(r) \qquad \text { for}\; r \in [a,b] , \end{aligned}$$

where $(\rho _0^{\varepsilon ,b},u_0^{\varepsilon ,b})(r)$ are defined in the appendix and satisfy that, for each (ε,b), there exists some $C_{\varepsilon ,b} > 0$ such that

2.14 $$\begin{aligned} 0< C_{\varepsilon ,b}^{-1} \le \rho _0^{\varepsilon ,b}(r) \le C_{\varepsilon ,b} < \infty . \end{aligned}$$

We define the spatial domain $\Omega _t$ of the free boundary problem at time t≥0 to be

$$ \Omega _t = \big \{ \boldsymbol{x} \in \mathbb {R}^n \,: \, a \le |\boldsymbol{x}| \le b(t)\big \}. $$

Here, system (2.10) is just system (1.9) with $(\mu ,\lambda ) = (\rho ,0)$ written with $u = \frac{m}{\rho }$. Owing to the fact that, under this construction as a free boundary problem, we can prove the existence of smooth solutions via a similar approach to [42], which have a strictly positive density $\rho ^{\varepsilon ,b} > 0$ due to (2.14) so that the fluid velocity $u^{\varepsilon ,b}$ is well defined and hence this construction is equivalent. More precisely, there exists $\tilde{C}_{\varepsilon ,b,T} > 0$ such that, for t∈[0,T],

2.15 $$\begin{aligned} 0< \tilde{C}_{\varepsilon ,b,T}^{-1} \le \rho ^{\varepsilon ,b}(t,r) \le \tilde{C}_{\varepsilon ,b,T} < \infty . \end{aligned}$$

Note that the boundary condition in (2.12) on the free boundary is a stress-free boundary condition, making the problem physically relevant, due to the fact that, under such an assumption, the boundary moves with the velocity of the fluid as modeled by the free boundary. We denote the solution of the free boundary problem (2.10)–(2.13) by $(\rho ^{\varepsilon ,b},u^{\varepsilon ,b})$, but will suppress the superscript when no confusion arises until §4 for simplicity.

#### Remark 2.9

From $(2.10)_1$ and (2.12), as in [26], we have

2.16 $$\begin{aligned} \rho (t,b(t)) \le \rho _0(b). \end{aligned}$$

The critical mass, as before, is defined for α>0 as

2.17 $$\begin{aligned} M^{\varepsilon ,b,\alpha }_\textrm{c}(\gamma ) = {\left\{ \begin{array}{ll} B_{n,\gamma ,\alpha }^{-\frac{n}{n - \alpha }} &  \text { for } \gamma = \frac{n + \alpha }{n},\\ \Big (\frac{\alpha B_{n,\gamma ,\alpha }}{n(\gamma - 1)} \Big )^{\frac{n(\gamma - 1)}{2n - \gamma (2n-\alpha )}} \Big (\frac{\alpha E^{\varepsilon ,b}_0}{\alpha - n(\gamma - 1)}\Big )^{\frac{\alpha - n(\gamma - 1)}{2n-\gamma (2n-\alpha )}} &  \text { for } \gamma \in (\frac{2n}{2n - \alpha }, \frac{n + \alpha }{n}), \end{array}\right. } \end{aligned}$$

where the total energy $E^{\varepsilon ,b}_0$ is defined in Definition B.7. $M^{\varepsilon ,\alpha }_\textrm{c}(\gamma )$ is defined the same way as $M^{\varepsilon ,b,\alpha }_\textrm{c}(\gamma )$ in (2.17), except $E^{\varepsilon ,b}_0$ is replaced by $E^{\varepsilon }_0$, where $E^{\varepsilon }_0$ is defined in Definition B.7. The construction of $\rho ^{\varepsilon ,b}_0$ implies that the following equality for mass holds:

$$\begin{aligned} \int ^b_a \rho ^{\varepsilon ,b}_0(r)\, r^{n-1} {\, \mathrm d}r = \frac{M}{\omega _n}. \end{aligned}$$

We can simply use $(2.10)_1$ and (2.11)–(2.12) to prove the conservation of mass:

$$\begin{aligned} \frac{\textrm{d}}{\textrm{d}t} \int ^{b(t)}_a \rho (t,r)\, r^{n-1} {\, \mathrm d}r = (\rho u)(t,b(t))b(t)^{n-1} - \int ^{b(t)}_a (\rho u r^{n-1})_r(t,r) {\, \mathrm d}r = 0. \end{aligned}$$

Thus, for all t≥0,

$$\begin{aligned} \int ^{b(t)}_a \rho (t,r)\, r^{n-1} {\, \mathrm d}r = \frac{M}{\omega _n}. \end{aligned}$$

Using this, we can define the Lagrangian coordinates (τ,x)=(t,x(t,r)) as in [26] by

$$\begin{aligned} x(t,r) = \int ^r_a \rho (t,y)\,y^{n-1} {\, \mathrm d}y. \end{aligned}$$

It is direct to see that this is a smooth bijective mapping from [0,T]×[a,b(t)] to $[0,T] \times [0, \frac{M}{\omega _n}]$, which is incredibly useful due to the fact that the latter domain is fixed. Then we have

2.18 $$\begin{aligned} \nabla _{(t,r)} x = (-\rho u r^{n-1},\rho r^{n-1}), \,\,\,\, \nabla _{(t,r)} \tau = (1,0), \,\,\,\, \nabla _{(\tau ,x)} r = (u, \rho ^{-1}r^{1-n}), \,\,\,\,\nabla _{(\tau ,x)} t = (1,0). \end{aligned}$$

Under the changes of variables, the boundary conditions become

2.19 $$\begin{aligned} u(\tau ,0) = 0, \quad \big (p-\varepsilon \rho ^2(r^{n-1}u)_x\big )(\tau ,\frac{M}{\omega _n}) = 0 \qquad \,\, \text { for} \, \tau \in [0,T], \end{aligned}$$

and (2.10) can be written in the form:

2.20 $$\begin{aligned} {\left\{ \begin{array}{ll} \rho _\tau + \rho ^2(r^{n-1}u)_x = 0,\\ u_\tau + r^{n-1}p_x + \kappa \rho r^{n-1}(\Phi _\alpha * \rho )_x = \varepsilon r^{n-1}\big (\rho ^2(r^{n-1}u)_x\big )_x - (n-1)\varepsilon r^{n-2}\rho _xu. \end{array}\right. } \end{aligned}$$

## Uniform Estimates of the Approximate Solutions

In this section, we carefully derive uniform estimates of the approximate solutions constructed in §2.3 above.

### Estimates on the radial non-local potential

Given a radial function $f \in L^1(\mathbb {R}^n) \cap L^q_\textrm{loc}(\mathbb {R}^n \setminus \{\boldsymbol{0}\})$ for $q \in (\frac{n}{n - 1 - \alpha }, \infty )$, $(\Phi _\alpha *f)(\boldsymbol{x})$ is a radial function so that we are able to represent it as a convolution of some potentials with the radial function. Like in [5], we can represent the convolution by

$$\begin{aligned} (\Phi _\alpha *f)(\boldsymbol{x}) = \int ^{\infty }_0 K(|\boldsymbol{x}|,\eta ) f(\eta ) \eta ^{n-1} {\, \mathrm d}\eta , \end{aligned}$$

where

$$\begin{aligned} K(r,\eta ) {:=}{\left\{ \begin{array}{ll} \displaystyle \int _{\mathbb {S}^{n-1}} \frac{|r\boldsymbol{e_1}-\eta \boldsymbol{y}|^{-\alpha }}{-\alpha } {\, \mathrm d}\sigma (\boldsymbol{y}) &  \text { for } \alpha \ne 0, \\ \displaystyle \int _{\mathbb {S}^{n-1}} \log (|r\boldsymbol{e_1}-\eta \boldsymbol{y}|) {\, \mathrm d}\sigma (\boldsymbol{y}) \quad & \text { for } \alpha = 0, \\ \end{array}\right. } \end{aligned}$$

with $\boldsymbol{e_1} = (1,0,\cdots ,0)$. We now consider the differentiability of such a convolution and the potential that can be used to represent the derivative.

#### Lemma 3.1

(Continuity of the Potential). Let $\alpha \in (-1,n-1)$. Given a radial function $f \in L^1(\mathbb {R}^n) \cap L^q_\textrm{loc}(\mathbb {R}^n \setminus \{\boldsymbol{0}\})$ for some $q \in (\frac{n}{n - 1 - \alpha }, \infty )$ such that $\Phi _\alpha *f$ and $\nabla \Phi _\alpha *f$ are well-defined, then $(\Phi _\alpha *f)(\boldsymbol{x})$ is continuously differentiable on $\mathbb {R}^n \setminus \{\boldsymbol{0}\}$, and

$$\begin{aligned} (\nabla \Phi _\alpha *f)(\boldsymbol{x}) \cdot \frac{\boldsymbol{x}}{|\boldsymbol{x}|} = (\Phi _\alpha *f)_r(|\boldsymbol{x}|) = \int ^{\infty }_0 \omega (|\boldsymbol{x}|,\eta ) f(\eta )\,\eta ^{n-1}{\, \mathrm d}\eta \end{aligned}$$

is a radial function, where

$$ \omega (r,\eta ) {:=}\int _{\mathbb {S}^{n-1}} \nabla \Phi _\alpha (r\boldsymbol{e_1} - \eta \boldsymbol{y}) \cdot \boldsymbol{e_1} {\, \mathrm d}\sigma (\boldsymbol{y}). $$

Moreover, there exists C=C(α,n)>0 such that

3.1 $$\begin{aligned} \begin{aligned} {\left\{ \begin{array}{ll} \displaystyle |\omega (r,\eta )| \le C (r\eta )^{-\frac{\alpha + 1}{2}} &  \text { for } \alpha \in (-1,n-2), \\ \displaystyle \omega (r,\eta ) = \frac{\omega _n 1\!\!1_{(0,r)}(\eta )}{r^{n-1}} &  \text { for } \alpha = n-2, \\ \displaystyle |\omega (r,\eta )| \le C(r\eta )^{-\frac{n-1}{2}} |r-\eta |^{n-2-\alpha } &  \text { for } \alpha \in (n-2,n-1), \end{array}\right. } \end{aligned} \end{aligned}$$

where $1\!\!1_{(0,r)}$ is the indicator function on interval (0, *r*).

#### Proof

We divide the proof into five steps.

1. For $\alpha \in (0,n-1)$, since $q > \frac{n}{n - 1 - \alpha }$, then, by Theorem A.1, there exists $C_1 > 0$ such that, for ε>0, the function:

$$\begin{aligned} \boldsymbol{z}\, \mapsto \, \int _{B_1(\boldsymbol{x})} \Phi _\alpha (\boldsymbol{z} - \boldsymbol{y}) 1\!\!1_{B_\varepsilon (\boldsymbol{x})}(\boldsymbol{y}) f(\boldsymbol{y}) {\, \mathrm d}\boldsymbol{y} \end{aligned}$$

is in $C^{0,1}(\overline{B_1(\boldsymbol{x})})$ for $|\boldsymbol{x}| > 0$, with Lipschitz constant bounded by $C_1 \Vert f \Vert _{L^q(B_\varepsilon (\boldsymbol{x}))}$ for ε>0 small enough, where $\overline{B_1(\boldsymbol{x})}$ denotes the closure of the open ball $B_1(\boldsymbol{x})$ of radius 1 centered at $\boldsymbol{x}$. For $\alpha \in (0,n)$ and $0<|\boldsymbol{z}| \le 1$,

$$\begin{aligned} \begin{aligned}&\frac{1}{|\boldsymbol{z}|} \bigg |\int _{B_\varepsilon (\boldsymbol{x})} (\Phi _\alpha (\boldsymbol{x} + \boldsymbol{z} - \boldsymbol{y}) - \Phi _\alpha (\boldsymbol{x} - \boldsymbol{y})) f(\boldsymbol{y}) {\, \mathrm d}\boldsymbol{y} \bigg | \\&\quad = \frac{1}{\alpha |\boldsymbol{z}|} \bigg | \int _{B_1(\boldsymbol{x})} \left( |\boldsymbol{x} + \boldsymbol{z} - \boldsymbol{y}|^{-\alpha } - |\boldsymbol{x} - \boldsymbol{y}|^{-\alpha } \right) 1\!\!1_{B_\varepsilon (\boldsymbol{x})}(\boldsymbol{y}) f(\boldsymbol{y}) {\, \mathrm d}\boldsymbol{y} \bigg | \\&\quad \le \frac{C_1}{\alpha } \Vert f \Vert _{L^q(B_\varepsilon (\boldsymbol{x}))} \rightarrow 0 \qquad \,\, \text { uniformly in}\, \boldsymbol{z}\,\, \text {as } \varepsilon \rightarrow 0. \end{aligned} \end{aligned}$$

2. For $\alpha \in (-1,0]$, since $q > \frac{n}{n - 1 - \alpha }$, then, by Theorem A.1, there exists $C_2 > 0$ such that, for ε>0, the function:

$$\begin{aligned} \boldsymbol{z}\, \mapsto \, \int _{B_1(\boldsymbol{x})} |\boldsymbol{z} - \boldsymbol{y}|^{-(\alpha + 1)} 1\!\!1_{B_\varepsilon (\boldsymbol{x})}(\boldsymbol{y}) f(\boldsymbol{y}) {\, \mathrm d}\boldsymbol{y} \end{aligned}$$

is in $C(\overline{B_1(\boldsymbol{x})})$ for $|\boldsymbol{x}| > 0$ such that, for ε small enough,

$$\begin{aligned} \Big \Vert \int _{B_1(\boldsymbol{x})} |\cdot - \boldsymbol{y}|^{-(\alpha + 1)} 1\!\!1_{B_\varepsilon (\boldsymbol{x})}(\boldsymbol{y}) f(\boldsymbol{y}) {\, \mathrm d}\boldsymbol{y} \Big \Vert _{C(B_1(\boldsymbol{x}))} \le C_2 \Vert f\Vert _{L^q(B_\varepsilon (\boldsymbol{x}))}. \end{aligned}$$

For $0<|\boldsymbol{z}| \le 1$, $\boldsymbol{0} \not \in \{ \boldsymbol{x} + s \boldsymbol{z} - \boldsymbol{y} : \, s \in [0,1] \}$ for *a.e.* $\boldsymbol{y}\in B_1(\boldsymbol{x})$. Thus, for *a.e.* $\boldsymbol{y}\in B_1(\boldsymbol{x})$ (that is, for $\boldsymbol{y}$ such that $\boldsymbol{0} \not \in \{ \boldsymbol{x} + s \boldsymbol{z} - \boldsymbol{y} \,: \, s \in [0,1] \}$), we have

$$\begin{aligned} |\Phi _\alpha (\boldsymbol{x} + \boldsymbol{z} - \boldsymbol{y}) - \Phi _\alpha (\boldsymbol{x} - \boldsymbol{y})| = \Big |\int ^1_0 \frac{\textrm{d}}{\textrm{d}s} \Phi _\alpha (\boldsymbol{x} + s \boldsymbol{z} - \boldsymbol{y}) {\, \mathrm d}s \Big | = \Big | \boldsymbol{z} \cdot \int ^1_0 \frac{\boldsymbol{x} + s \boldsymbol{z} - \boldsymbol{y}}{|\boldsymbol{x} + s \boldsymbol{z} - \boldsymbol{y}|^{\alpha + 2}} {\, \mathrm d}s \Big |. \end{aligned}$$

Therefore, for $\alpha \in (-1,0]$ and $0<|\boldsymbol{z}| \le 1$,

$$\begin{aligned} \begin{aligned}&\frac{1}{|\boldsymbol{z}|} \bigg | \int _{B_\varepsilon (\boldsymbol{x})} (\Phi _\alpha (\boldsymbol{x} + \boldsymbol{z} - \boldsymbol{y}) - \Phi _\alpha (\boldsymbol{x} - \boldsymbol{y})) f(\boldsymbol{y}) {\, \mathrm d}\boldsymbol{y} \bigg | \\&\quad \le \int ^1_0 \int _{B_1(\boldsymbol{x})}|\boldsymbol{x} + s \boldsymbol{z} - \boldsymbol{y}|^{-(\alpha + 1)} 1\!\!1_{B_\varepsilon (\boldsymbol{x})}(\boldsymbol{y}) |f(\boldsymbol{y})| {\, \mathrm d}\boldsymbol{y} {\, \mathrm d}s \\&\quad \le C_2 \Vert f \Vert _{L^q(B_\varepsilon (\boldsymbol{x}))} \rightarrow 0 \qquad \,\,\text { as } \varepsilon \rightarrow 0. \end{aligned} \end{aligned}$$

3. For $\alpha \in (-1,n - 1)$, we see that, for fixed ε>0,

$$\begin{aligned} \frac{|\Phi _\alpha (\boldsymbol{x} + \boldsymbol{z} - \boldsymbol{y}) - \Phi _\alpha (\boldsymbol{x} - \boldsymbol{y}) - \boldsymbol{z} \cdot \nabla \Phi _\alpha (\boldsymbol{x} - \boldsymbol{y})|}{|\boldsymbol{z}|} \longrightarrow 0 \end{aligned}$$

uniformly for $\boldsymbol{y} \in \mathbb {R}^n \setminus B_\varepsilon (\boldsymbol{x})$ as $\boldsymbol{z} \rightarrow \boldsymbol{0}$. Since $f \in L^1$, we obtain

$$\begin{aligned} \begin{aligned}&\frac{1}{|\boldsymbol{z}|} \bigg | \int _{B_\varepsilon ^c(\boldsymbol{x})} \big (\Phi _\alpha (\boldsymbol{x} + \boldsymbol{z} - \boldsymbol{y})-\Phi _\alpha (\boldsymbol{x}- \boldsymbol{y})\big ) f(\boldsymbol{y}) {\, \mathrm d}\boldsymbol{y} - \boldsymbol{z} \cdot \int _{B^c_\varepsilon (\boldsymbol{x})} \nabla \Phi _\alpha (\boldsymbol{x} - \boldsymbol{y}) f(\boldsymbol{y}) {\, \mathrm d}\boldsymbol{y} \bigg | \\&\,\,\longrightarrow 0 \qquad \text { as} \boldsymbol{z} \rightarrow 0 . \end{aligned} \end{aligned}$$

Then we conclude that $(\Phi _\alpha *f)(\boldsymbol{x})$ is differentiable on $\mathbb {R}^n \setminus \{\textbf{0}\}$ with $\nabla (\Phi _\alpha *f)(\boldsymbol{x}) = (\nabla \Phi _\alpha *f)(\boldsymbol{x})$.

4. As in [5], we can write

$$ (\nabla \Phi _\alpha *f)(\boldsymbol{x}) = \int ^{\infty }_0 \omega (|\boldsymbol{x}|,\eta ) f(\eta ) \eta ^{n-1}{\, \mathrm d}\eta \, \frac{\boldsymbol{x}}{|\boldsymbol{x}|}. $$

Then we can see that $(\nabla \Phi _\alpha *f)(\boldsymbol{x}) \cdot \frac{\boldsymbol{x}}{|\boldsymbol{x}|} =(\Phi _\alpha *f)_r(|\boldsymbol{x}|)$ is a radial function. Now, as in [20], we see that, for r>0,

$$\begin{aligned} (\Phi _\alpha *f)_r( r) = \int ^{\infty }_0 \Big ( \int _{\mathbb {S}^{n-1}} \frac{r\boldsymbol{e_1} - \eta \boldsymbol{y}}{|r\boldsymbol{e_1} - \eta \boldsymbol{y}|^{\alpha + 2}} \cdot \boldsymbol{e_1} {\, \mathrm d}\sigma (\boldsymbol{y}) \Big ) f(\eta ) \eta ^{n-1} {\, \mathrm d}\eta . \end{aligned}$$

Now, considering the inner integral and applying a change of coordinates to the polyspherical coordinates given by

$$ \boldsymbol{y} = (\cos \theta , \sin {\theta } \,\hat{\boldsymbol{z}}) $$

with $\theta \in [0,\pi )$ and $\hat{\boldsymbol{z}} \in \mathbb {S}^{n-2}$, we have

$$\begin{aligned} \begin{aligned}&\int _{\mathbb {S}^{n-1}} \frac{r\boldsymbol{e_1} - \eta \boldsymbol{y}}{|r\boldsymbol{e_1} - \eta \boldsymbol{y}|^{\alpha + 2}} \cdot \boldsymbol{e_1} {\, \mathrm d}\sigma (\boldsymbol{y}) \\&\quad = \omega _{n-1} \int ^\pi _0 \big ((r - \eta \cos {\theta })^2 + (\eta \sin {\theta })^2 \big )^{-\frac{\alpha + 2}{2}}(r - \eta \cos {\theta })|\sin {\theta }|^{n-2}{\, \mathrm d}\theta . \end{aligned} \end{aligned}$$

Looking at the integrand, we have

3.2 $$\begin{aligned}&\Big | \big ((r - \eta \cos {\theta })^2 + (\eta \sin {\theta })^2 \big )^{-\frac{\alpha + 2}{2}}(r - \eta \cos {\theta })|\sin {\theta }|^{n-2} \Big | \nonumber \\&\quad \le \big ((r - \eta )^2 + 2r\eta (1-\cos {\theta }) \big )^{-\frac{\alpha + 1}{2}}|\sin {\theta }|^{n-2} \nonumber \\&\quad \le \big ((r - \eta )^2 + r\eta \theta ^2(1 - \frac{\pi ^2}{12}) \big )^{-\frac{\alpha + 1}{2}}\theta ^{n-2}. \end{aligned}$$

For ζ>0, using (3.2), we obtain that, for α>n-2,

3.3 $$\begin{aligned}&\Big | \int ^\pi _0 \big ((r - \eta \cos {\theta })^2 + (\eta \sin {\theta })^2 \big )^{-\frac{\alpha + 2}{2}}(r - \eta \cos {\theta })|\sin {\theta }|^{n-2}{\, \mathrm d}\theta \Big |\nonumber \\&\quad \le \int ^\pi _0 \big ((r - \eta )^2 + r\eta \theta ^2(1 - \frac{\pi ^2}{12}) \big )^{-\frac{\alpha + 1}{2}} \theta ^{n-2}{\, \mathrm d}\theta \nonumber \\&\quad \le \int ^\pi _\zeta \big (r\eta \theta ^2(1 - \frac{\pi ^2}{12}) \big )^{-\frac{\alpha + 1}{2}}\theta ^{n-2}{\, \mathrm d}\theta + \int ^\zeta _0 |r - \eta |^{-(\alpha + 1)}\theta ^{n-2}{\, \mathrm d}\theta \nonumber \\&\quad =\big (r\eta (1 - \frac{\pi ^2}{12}) \big )^{-\frac{\alpha + 1}{2}} \Big [ \frac{\theta ^{n-2-\alpha }}{n-2-\alpha } \Big ]^\pi _\zeta + |r - \eta |^{-(\alpha + 1)} \Big [\frac{\theta ^{n-1}}{n-1} \Big ]^\zeta _0. \end{aligned}$$

Letting $\zeta = \min \{\pi , \frac{|r-\eta |}{\sqrt{r\eta }}\}$, then there exists C>0 such that

3.4 $$\begin{aligned}&\Big | \int ^\pi _0 \big ((r - \eta \cos {\theta })^2 + (\eta \sin {\theta })^2 \big )^{-\frac{\alpha + 2}{2}} (r - \eta \cos {\theta })|\sin {\theta }|^{n-2}{\, \mathrm d}\theta \Big | \nonumber \\&\quad \le C(r\eta )^{-\frac{n-1}{2}} |r-\eta |^{n-2-\alpha }. \end{aligned}$$

5. Let r>0. Then, for $r \gg \varepsilon > 0$, we use (3.2) to obtain that, for $s \in (r-\frac{\varepsilon }{2},r+\frac{\varepsilon }{2})$ and $\eta \in (0,\infty )\setminus (r - \varepsilon , r + \varepsilon )$,

$$\begin{aligned} \Big |\big ((s - \eta \cos {\theta })^2 + (\eta \sin {\theta })^2 \big )^{-\frac{\alpha + 2}{2}}(s - \eta \cos {\theta })|\sin {\theta }|^{n-2} \Big | \le C(\varepsilon ). \end{aligned}$$

Hence, by the Lebesgue dominated convergence theorem, we have

$$\begin{aligned} \begin{aligned} \lim _{s \rightarrow r} \int _{(0,\infty )\setminus (r - \varepsilon , r +\varepsilon )} \omega (s,\eta ) f(\eta )\,\eta ^{n-1} {\, \mathrm d}\eta = \int _{(0,\infty )\setminus (r - \varepsilon ,r + \varepsilon )} \omega (r,\eta ) f(\eta ) \,\eta ^{n-1} {\, \mathrm d}\eta . \end{aligned} \end{aligned}$$

By (3.4) and Lemma A.1, for $s\in (r-\varepsilon /2,r+\varepsilon /2)$ and $\varepsilon \ll r$, we have

$$ \begin{aligned} \left| \int _{r-\varepsilon }^{r+\varepsilon } \omega (s,\eta )f(\eta )\eta ^{n-1}\,d\eta \right|&\le C_r\int _{r-\varepsilon }^{r+\varepsilon } |s-\eta |^{n-2-\alpha }|f(\eta )|\,d\eta \\&\le C_r\Vert f\Vert _{L^q(r-\varepsilon ,r+\varepsilon )} \left( \int _{r-\varepsilon }^{r+\varepsilon } |s-\eta |^{(n-2-\alpha )q'}\,d\eta \right) ^{1/q'} \\&\le C_r\Vert f\Vert _{L^q(r-\varepsilon ,r+\varepsilon )} \varepsilon ^{\,n-1-\alpha -\frac{1}{q}} \rightarrow 0 , \end{aligned} $$

as $\varepsilon \rightarrow 0$, where $q'=\frac{q}{q-1}$. Since

$$\begin{aligned} \lim _{\varepsilon \rightarrow 0} \int _{(0,\infty )\setminus (r - \varepsilon , r +\varepsilon )}\omega (r,\eta )f(\eta )\,\eta ^{n-1}{\, \mathrm d}\eta = \int ^{\infty }_0 \omega (r,\eta ) f(\eta )\,\eta ^{n-1} {\, \mathrm d}\eta , \end{aligned}$$

we conclude that $\lim _{s \rightarrow r} (\Phi _\alpha *f)_r(s) = (\Phi _\alpha *f)_r(r)$.

For $\alpha \in (-1,n-2)$, following the same approach as (3.3), we have

$$\begin{aligned} \begin{aligned} |\omega (r,\eta )| \le C \int ^\pi _0 \big (r\eta \theta ^2 \big )^{-\frac{\alpha + 1}{2}}\theta ^{n-2}{\, \mathrm d}\theta \le C (r\eta )^{-\frac{\alpha + 1}{2}}. \end{aligned} \end{aligned}$$

Thus, it follows from the same approach as for α>n-2 that $(\Phi _\alpha *f)_r$ is continuous for $\alpha \in (-1,n-2)$.

For α=n-2, since $\Phi _{n-2}$ is the fundamental solution of the Laplace equation up to a constant, we see that, for any radial function $f \in C^\infty _\textrm{c}(\mathbb {R}^n)$,

$$\begin{aligned} \big (r^{n-1}(\Phi _{n-2} * f)_r\big )_r(r) = r^{n-1} \Delta (\Phi _{n-2} * f)(r) = \omega _n r^{n-1} f(r). \end{aligned}$$

Since it is classically differentiable, then $r^{n-1}(\Phi _{n-2} * f)_r$ and hence $(\Phi _{n-2} * f)_r$ are continuous. Furthermore, we integrate it over *r* to obtain

$$ \int ^{\infty }_0 \omega (r,\eta ) f(\eta ) \eta ^{n-1}{\, \mathrm d}\eta = (\Phi _{n-2} * f)_r(r) = \frac{\omega _n}{r^{n-1}} \int ^r_0 f(\eta ) \eta ^{n-1} {\, \mathrm d}\eta . $$

This implies

$$ \int ^{\infty }_0 \Big ( \omega (r,\eta ) - \frac{\omega _n 1\!\!1_{(0,r)}(\eta )}{r^{n-1}} \Big ) f(\eta ) \eta ^{n-1} {\, \mathrm d}\eta = 0 $$

for any radial function $f \in C^\infty _\textrm{c}(\mathbb {R}^n)$. Then, by the fundamental lemma of the calculus of variations, we conclude

$$\begin{aligned} \omega (r,\eta ) = \frac{\omega _n 1\!\!1_{(0,r)}(\eta )}{r^{n-1}}. \end{aligned}$$

□

#### Remark 3.2

Notice that, for fixed t>0, density $\rho (t,\boldsymbol{x})$ satisfies the assumption on *f* in Lemma 3.1, as $\rho (t,\boldsymbol{x})$ is smooth in $\Omega _t$ and 0 everywhere else by convention, $\rho (t,\cdot ) \in L^q_\textrm{loc}(\mathbb {R}^n \setminus \{\boldsymbol{0}\})$ for all $q \in [1,\infty ]$, and $\rho (t,\cdot )\in L^1(\mathbb {R}^n)$ since it has finite mass *M*. Thus, we can apply Lemma 3.1 for $\rho (t,\boldsymbol{x})$ regarding the solution of the free boundary problem.

#### Remark 3.3

Alves-Grafakos-Tzavaras in [2] proved the uniform estimates for a bilinear operator that relates to a divergence form of the Riesz potential. That is, for sufficiently regular *f* on $\mathbb {R}^n$, it is shown that there exists $S_\alpha (f) = I_\alpha (f,f)$ such that

$$ f \nabla (\Phi _\alpha * f) = \nabla \cdot S_{n-\alpha }(f). $$

Moreover, for $1< p,q < \frac{n}{\alpha }$ and r≥1 such that

$$ \frac{1}{p} + \frac{1}{q} = \frac{1}{r} + \frac{\alpha }{n}, $$

then, for $f \in L^p(\mathbb {R}^n)$ and $g \in L^q(\mathbb {R}^n)$,

$$ \Vert I_\alpha (f,g) \Vert _{L^r(\mathbb {R}^n)} \le C \Vert f \Vert _{L^p(\mathbb {R}^n)} \Vert g \Vert _{L^q(\mathbb {R}^n)}. $$

With this, some compensated integrability can be obtained by using the HLS inequality to estimate $f \nabla (\Phi _\alpha * f)$, so that the weak solutions in the divergence form can be framed. However, our approximate solutions (ρ,u) of the free boundary problem are only defined and differentiable on $\Omega _t$, as such the construction of $S_\alpha$ no longer works in the context of our problem, where it can be seen that $S_\alpha (\rho )$ defined in [2] is not differentiable. Moreover, since we have no control over the derivatives of the gradient of *u*, unlike [2] where it is assumed in the definition of the weak solutions, it is difficult to control the potential term in the same way.

### Energy estimates

Now we make some uniform energy estimates of the solutions of (2.10)–(2.13).

#### Lemma 3.4

(Energy Estimates). For $\alpha \in (-1, n-1)$, then any smooth solution (ρ,u)(t,r) of the free boundary problem (2.10)–(2.13) satisfies the following energy estimate:

3.5 $$\begin{aligned}&\int ^{b(t)}_a \rho \Big (\frac{1}{2}u^2 + e(\rho ) + \frac{\kappa }{2} (\Phi _\alpha * \rho ) \Big ) r^{n-1} {\, \mathrm d}r + \varepsilon \int ^t_0\int ^{b(s)}_a \rho \Big (u_r^2 + (n-1)\frac{u^2}{r^2} \Big ) r^{n-1} {\, \mathrm d}r{\, \mathrm d}s \nonumber \\&\qquad \, + \varepsilon (n-1) \int ^t_0 (\rho u^2)(s,b(s))b(s)^{n-2} {\, \mathrm d}s\nonumber \\&\quad = \int ^b_a \rho _0 \Big (\frac{1}{2}u_0^2 + e(\rho _0) + \frac{\kappa }{2} (\Phi _\alpha * \rho _0) \Big )r^{n-1} {\, \mathrm d}r. \end{aligned}$$

In particular, we have the following estimates:

Case 1: $\alpha \in (0,n-1)$ and κ=-1 with γ>1,

$$\begin{aligned}&\int ^{b(t)}_a \rho \Big (\frac{1}{2}u^2 + e(\rho ) - \frac{1}{2} (\Phi _\alpha * \rho ) \Big )r^{n-1} {\, \mathrm d}r \\&\qquad + \varepsilon \int ^t_0\int ^{b(s)}_a \rho \Big (u_r^2 + (n-1)\frac{u^2}{r^2}\Big ) r^{n-1} {\, \mathrm d}r{\, \mathrm d}s \\&\qquad + \varepsilon (n-1) \int ^t_0 (\rho u^2)(s,b(s))b(s)^{n-2} {\, \mathrm d}s\\&\quad = \int ^b_a \rho _0 \Big (\frac{1}{2}u_0^2 + e(\rho _0) - \frac{1}{2} (\Phi _\alpha * \rho _0)\Big )r^{n-1} {\, \mathrm d}r = E^{\varepsilon ,b}_0. \end{aligned}$$

Case 2: $\alpha \in (0,n-1)$ and κ=1 with $\gamma \in (\frac{2n}{2n - \alpha }, \frac{n + \alpha }{n} ]$ and $M < M^{\varepsilon ,b,\alpha }_\textrm{c}(\gamma )$,

3.6 $$\begin{aligned}&\int ^{b(t)}_a \rho \Big (\frac{1}{2}u^2 + C_\gamma e(\rho ) \Big )r^{n-1} {\, \mathrm d}r + \varepsilon \int ^t_0\int ^{b(s)}_a \rho \Big (u_r^2 + (n-1)\frac{u^2}{r^2} \Big ) r^{n-1} {\, \mathrm d}r{\, \mathrm d}s\nonumber \\&\qquad + \varepsilon (n-1)\int ^t_0 (\rho u^2)(s,b(s))b(s)^{n-2} {\, \mathrm d}s \nonumber \\&\quad \le \int ^b_a \rho _0 \Big (\frac{1}{2}u_0^2 + e(\rho _0) + \frac{\kappa }{2} (\Phi _\alpha * \rho _0) \Big )r^{n-1} {\, \mathrm d}r = E^{\varepsilon ,b}_0, \end{aligned}$$

where the positive constant $C_\gamma > 0$ is defined as

$$ C_\gamma {:=}{\left\{ \begin{array}{ll} 1 - B_{n,\gamma ,\alpha }M^{\frac{n - \alpha }{n}} \quad &  \text { for } \gamma = \frac{n + \alpha }{n},\\ \frac{\alpha - n(\gamma -1)}{\alpha } &  \text { for } \gamma \in (\frac{2n}{2n - \alpha }, \frac{n + \alpha }{n} ). \end{array}\right. } $$

Case 3: $\alpha \in (0,n-1)$ and κ=1 with $\gamma > \frac{n + \alpha }{n}$,

$$\begin{aligned}&\frac{1}{2} \int ^{b(t)}_a \rho \left( u^2 + e(\rho ) \right) r^{n-1} {\, \mathrm d}r + \varepsilon \int ^t_0\int ^{b(s)}_a \rho \Big (u_r^2 + (n-1)\frac{u^2}{r^2} \Big ) r^{n-1} {\, \mathrm d}r{\, \mathrm d}s \\&\,\, + \varepsilon (n-1)\int ^t_0 (\rho u^2)(s,b(s))b(s)^{n-2} {\, \mathrm d}s \le C(M,E^{\varepsilon ,b}_0). \end{aligned}$$

Case 4: $$\alpha \in (-1,0]$$, $$\kappa \in \{-1,1\}$$ with γ>1,

3.7 $$\begin{aligned}&\frac{1}{2} \int ^{b(t)}_a \rho \left( u^2 + e(\rho ) + k_\alpha (1 + r^2) \right) r^{n-1} {\, \mathrm d}r + \varepsilon \int ^t_0\int ^{b(s)}_a \rho \Big (u_r^2 + \frac{u^2}{r^2} \Big ) r^{n-1} {\, \mathrm d}r {\, \mathrm d}s\nonumber \\&\,\,+ \varepsilon (n-1) \int ^t_0 (\rho u^2)(s,b(s)) b(s)^{n-2} {\, \mathrm d}s \le C(M,E^{\varepsilon ,b}_0,T). \end{aligned}$$

#### Proof

We divide the proof into six steps.

1. Multiplying $(2.20)_2$ in Lagrangian coordinates by *u* and integrating over *x* yield

3.8 $$\begin{aligned}&\frac{1}{2}\frac{\textrm{d}}{\textrm{d}\tau }\int ^{\frac{M}{\omega _n}}_0 u^2 {\, \mathrm d}x + \int ^{\frac{M}{\omega _n}}_0 \big (p-\varepsilon \rho ^2(r^{n-1}u)_x\big )_xur^{n-1} {\, \mathrm d}x \nonumber \\&\quad = -\varepsilon (n-1)\int ^{\frac{M}{\omega _n}}_0 \rho _xu^2r^{n-2} {\, \mathrm d}x - \kappa \int ^{\frac{M}{\omega _n}}_0 \rho u r^{n-1} (\Phi _\alpha * \rho )_x {\, \mathrm d}x. \end{aligned}$$

For the second term on the left-hand side (LHS) of (3.8), using the change of variables (2.18), the boundary conditions (2.19), the conservation of mass $(2.20)_1$, and integration by parts, we have

3.9 $$\begin{aligned}&\int _0^{\frac{M}{\omega _n}}\big (p(\rho )-\varepsilon \rho ^2(r^{n-1}u)_x\big )_xu\,r^{n-1} {\, \mathrm d}x\nonumber \\&\quad =-\int _0^{\frac{M}{\omega _n}}\big (p(\rho )-\varepsilon \rho ^2(r^{n-1}u)_x\big )(r^{n-1}u)_x {\, \mathrm d}x \nonumber \\&\quad =-\int _0^{\frac{M}{\omega _n}}p(\rho )(r^{n-1}u)_x {\, \mathrm d}x +\varepsilon \int _0^{\frac{M}{\omega _n}}\rho ^2\big ((r^{n-1}u)_x\big )^2{\, \mathrm d}x \nonumber \\&\quad =a_0\int _0^{\frac{M}{\omega _n}}\rho ^{\gamma -2}\rho _\tau {\, \mathrm d}x +\varepsilon \int _0^{\frac{M}{\omega _n}}\rho ^2\big (r^{n-1}u_x+(n-1)r^{n-2}r_xu\big )^2{\, \mathrm d}x\nonumber \\&\quad =\frac{\textrm{d}}{\textrm{d}\tau }\int _0^{\frac{M}{\omega _n}} e(\rho ){\, \mathrm d}x +\varepsilon \int _0^{\frac{M}{\omega _n}}\Big (\rho ^2(r^{n-1}u_x)^2 +(n-1)^2\frac{u^2}{r^2}+2(n-1)r^{n-2}\rho u u_x\Big ){\, \mathrm d}x. \end{aligned}$$

For the first term on the right-hand side (RHS) of (3.8), integrating by parts yields

3.10 $$\begin{aligned}&(n-1)\varepsilon \int _0^{\frac{M}{\omega _n}}\rho _xu^2r^{n-2} {\, \mathrm d}x \nonumber \\&\quad =-(n-1)\varepsilon \int _0^{\frac{M}{\omega _n}}\big (2 r^{n-2}\rho uu_x +(n-2)\frac{ u^2}{r^2}\big ){\, \mathrm d}x + \varepsilon (n-1)(\rho u^2 r^{n-2})(\tau ,\frac{M}{\omega _n}). \end{aligned}$$

For the second term on the RHS of (3.8), converting back to the Eulerian coordinates, and applying (2.19) and the conservation of mass $(2.10)_1$, we have

3.11 $$\begin{aligned}&\kappa \int ^{\frac{M}{\omega _n}}_0 \rho u\, (\Phi _\alpha * \rho )_x\, r^{n-1} {\, \mathrm d}x\nonumber \\&\quad = \kappa \int ^{b(t)}_a \rho u\, (\Phi _\alpha * \rho )_r\, r^{n-1} {\, \mathrm d}r \nonumber \\&\quad = \kappa \big (\rho u r^{n-1} (\Phi _\alpha * \rho ) \big )(t,b(t)) - \kappa \int ^{b(t)}_a (\rho u r^{n-1})_r (\Phi _\alpha * \rho ) {\, \mathrm d}r\nonumber \\&\quad = \kappa \big (\rho u r^{n-1} (\Phi _\alpha * \rho ) \big )(t,b(t)) + \kappa \int ^{b(t)}_a \rho _t (\Phi _\alpha * \rho )\,r^{n-1}{\, \mathrm d}r\nonumber \\&\quad = \frac{\kappa }{2} \big (\rho u r^{n-1} (\Phi _\alpha * \rho ) \big )(t,b(t)) + \frac{\kappa }{2} \int ^{b(t)}_a \frac{\partial }{\partial t} \big (\rho (\Phi _\alpha * \rho ) \big )\,r^{n-1}{\, \mathrm d}r\nonumber \\&\quad = \frac{\kappa }{2} \frac{\textrm{d}}{\textrm{d}t} \int ^{b(t)}_a\rho (\Phi _\alpha * \rho )\, r^{n-1}{\, \mathrm d}r\nonumber \\&\quad = \frac{\kappa }{2} \frac{\textrm{d}}{\textrm{d}\tau } \int ^{\frac{M}{\omega _n}}_0\Phi _\alpha * \rho {\, \mathrm d}x, \end{aligned}$$

where the 5th line follows from $K(r,\eta ) = K(\eta ,r)$, the Fubini theorem, and

$$\begin{aligned} \begin{aligned}&\int ^{b(t)}_a \rho (\Phi _\alpha * \rho )_t\,r^{n-1}{\, \mathrm d}r \\&\quad = \int ^{b(t)}_a \rho \Big ( \int ^{b(t)}_a K(r,\eta )\rho (t,\eta )\eta ^{n-1}{\, \mathrm d}\eta \Big )_t\,r^{n-1}{\, \mathrm d}r \\&\quad = (\rho u)(t,b(t))b(t)^{n-1} \int ^{b(t)}_a K(b(t),r) \rho (t,r)\, r^{n-1}{\, \mathrm d}r \\&\quad \quad + \int ^{b(t)}_a \rho \Big ( \int ^{b(t)}_a K(r,\eta )\rho _t(t,\eta )\eta ^{n-1}{\, \mathrm d}\eta \Big )\, r^{n-1}{\, \mathrm d}r \\&\quad = \big (\rho u r^{n-1} (\Phi _\alpha * \rho ) \big )(t,b(t)) + \int ^{b(t)}_a \rho _t \Big ( \int ^{b(t)}_a K(\eta ,r)\rho (t,r)r^{n-1}{\, \mathrm d}r \Big )\,\eta ^{n-1}{\, \mathrm d}\eta \\&\quad = \big (\rho u r^{n-1} (\Phi _\alpha * \rho ) \big )(t,b(t)) + \int ^{b(t)}_a \rho _t (\Phi _\alpha * \rho )\, r^{n-1}{\, \mathrm d}r. \end{aligned} \end{aligned}$$

Plugging (3.9)–(3.11) into (3.8), we obtain

3.12 $$\begin{aligned}&\frac{{\, \mathrm d}}{{\, \mathrm d}\tau } \bigg \{ \int ^{\frac{M}{\omega _n}}_0 \Big (\frac{1}{2}u^2 + e(\rho ) + \frac{\kappa }{2}(\Phi _\alpha * \rho )\Big ) {\, \mathrm d}x \bigg \} +\varepsilon \int _0^{\frac{M}{\omega _n}}\big (\rho ^2(r^{n-1}u_x)^2 + (n-1)\frac{u^2}{r^2}\big ){\, \mathrm d}x \nonumber \\&\,\,+ \varepsilon (n-1)(\rho u^2 r^{n-2})(\tau ,\frac{M}{\omega _n})=0. \end{aligned}$$

Converting (3.12) into the Eulerian coordinates, we have

$$\begin{aligned} \begin{aligned}&\frac{\textrm{d}}{\textrm{d}t} \int ^{b(t)}_a \rho \Big (\frac{1}{2}u^2 + e(\rho ) + \frac{\kappa }{2} (\Phi _\alpha * \rho )\Big )\,r^{n-1} {\, \mathrm d}r + \varepsilon \int ^{b(t)}_a \rho \Big (u_r^2 + (n-1)\frac{u^2}{r^2} \Big )\,r^{n-1} {\, \mathrm d}r \\&\,\, + \varepsilon (n-1) (\rho u^2)(t,b(t))b(t)^{n-2} = 0. \end{aligned} \end{aligned}$$

Integrating over [0, *t*], we derive the first part of this lemma:

3.13 $$\begin{aligned}&\int ^{b(t)}_a \rho \Big (\frac{1}{2}u^2 + e(\rho ) + \frac{\kappa }{2} (\Phi _\alpha * \rho )\Big )\,r^{n-1} {\, \mathrm d}r + \varepsilon \int ^t_0\int ^{b(s)}_a \rho \Big (u_r^2 + (n-1)\frac{u^2}{r^2} \Big )\,r^{n-1} {\, \mathrm d}r{\, \mathrm d}s \nonumber \\&\quad \quad + (n-1)\varepsilon \int ^t_0 (\rho u^2)(s,b(s))b(s)^{n-2} {\, \mathrm d}s \nonumber \\&\quad = \int ^b_a \rho _0 \Big (\frac{1}{2}u_0^2 + e(\rho _0) + \frac{\kappa }{2} (\Phi _\alpha * \rho _0) \Big )\,r^{n-1} {\, \mathrm d}r. \end{aligned}$$

Then Case 1 follows directly from (3.13).

2. For Case 2 & 3 (*i.e.,* κ=1), we need to control the potential energy term by the internal energy term. This is done by applying the Hölder inequality to the potential term and then the variation of the HLS inequality from Lemma A.3, and finally by using the interpolation as follows:

3.14 $$\begin{aligned}&\frac{1}{2}\int ^{b(t)}_a \rho (\Phi _\alpha * \rho ) r^{n-1} {\, \mathrm d}r \nonumber \\&\quad = \frac{1}{2 \omega _n}\int _{\Omega _t} \big (\rho (\Phi _\alpha * \rho )\big )(t,\boldsymbol{y}) {\, \mathrm d}\boldsymbol{y} \nonumber \\&\quad \le \frac{1}{2 \alpha \omega _n} \bigg | \int _{\Omega _t} \big (\rho (|\cdot |^{-\alpha } * \rho )\big )(t,\boldsymbol{y}) {\, \mathrm d}\boldsymbol{y} \bigg |\nonumber \\&\quad \le \frac{C_*(\alpha ,\gamma ,n)}{2 \alpha \omega _n} \Vert \rho \Vert ^{\frac{\gamma (2n - \alpha ) - 2n}{n(\gamma -1)}}_{L^1(\Omega _t)} \Vert \rho \Vert ^{\frac{\alpha \gamma }{n(\gamma - 1)}}_{L^\gamma (\Omega _t)} \nonumber \\&\quad = \frac{C_*(\alpha ,\gamma ,n)}{2 \alpha }\omega _n^{\frac{\alpha }{n(\gamma - 1)} - 1} M^{\frac{\gamma (2n - \alpha ) - 2n}{n(\gamma -1)}} \Big ( \int ^{b(t)}_a \rho ^\gamma r^{n-1} {\, \mathrm d}r \Big )^\frac{\alpha }{n(\gamma - 1)}\nonumber \\&\quad = \frac{C_*(\alpha ,\gamma ,n)}{2 \alpha }\omega _n^{\frac{\alpha }{n(\gamma - 1)} - 1} \Big (\frac{\gamma - 1}{a_0} \Big )^{\frac{\alpha }{n(\gamma - 1)}} M^{\frac{\gamma (2n - \alpha ) - 2n}{n(\gamma -1)}} \Big ( \int ^{b(t)}_a \rho e(\rho ) r^{n-1} {\, \mathrm d}r \Big )^\frac{\alpha }{n(\gamma - 1)} \nonumber \\&\quad = B_{n,\gamma ,\alpha } M^{\frac{\gamma (2n - \alpha ) - 2n}{n(\gamma -1)}} \Big ( \int ^{b(t)}_a \rho e(\rho ) r^{n-1} {\, \mathrm d}r \Big )^\frac{\alpha }{n(\gamma - 1)}. \end{aligned}$$

Thus, applying (3.14), we obtain

3.15 $$\begin{aligned}&\int ^{b(t)}_a \rho \Big (e(\rho ) + \frac{1}{2} (\Phi _\alpha * \rho )\Big )r^{n-1} {\, \mathrm d}r\nonumber \\&\quad \ge \int ^{b(t)}_a \rho e(\rho )r^{n-1} {\, \mathrm d}r - B_{n,\gamma ,\alpha } M^{\frac{\gamma (2n - \alpha ) - 2n}{n(\gamma -1)}} \Big ( \int ^{b(t)}_a \rho e(\rho ) r^{n-1} {\, \mathrm d}r \Big )^\frac{\alpha }{n(\gamma - 1)}. \end{aligned}$$

3. For Case 3, $\gamma > \frac{n + \alpha }{n}$ that implies that $\frac{\alpha }{n(\gamma - 1)} < 1$. Thus, using (3.15) and the Hölder inequality, we obtain

$$\begin{aligned} \begin{aligned}&\int ^{b(t)}_a \rho \Big ( e(\rho ) + \frac{1}{2} (\Phi _\alpha * \rho ) \Big )r^{n-1} {\, \mathrm d}r \ge \frac{1}{2}\int ^{b(t)}_a \rho e(\rho )r^{n-1} {\, \mathrm d}r - C(M). \end{aligned} \end{aligned}$$

4. Now, for Case 2, we first consider the case that $\gamma = \frac{n + \alpha }{n}$. Then, from (3.15), we have

$$\begin{aligned} \begin{aligned}&\int ^{b(t)}_a \rho \Big ( e(\rho ) + \frac{1}{2} (\Phi _\alpha * \rho )\Big )r^{n-1} {\, \mathrm d}r \\&\quad \ge \int ^{b(t)}_a \rho e(\rho )r^{n-1} {\, \mathrm d}r - B_{n,\gamma ,\alpha } M^{\frac{n - \alpha }{n}}\int ^{b(t)}_a \rho e(\rho ) r^{n-1} {\, \mathrm d}r \\&\quad = C_\gamma \int ^{b(t)}_a \rho e(\rho )r^{n-1} {\, \mathrm d}r, \end{aligned} \end{aligned}$$

where $C_\gamma > 0$, as we have assumed that $M < M^{\varepsilon ,b,\alpha }_\textrm{c}(\gamma )$. Now, considering the remaining case $\gamma \in (\frac{2n}{2n - \alpha },\frac{n + \alpha }{n})$, inspired by (3.15), we define

$$\begin{aligned} F(s) = s - B_{n,\gamma ,\alpha } M^{\frac{\gamma (2n - \alpha ) - 2n}{n(\gamma -1)}}s^\frac{\alpha }{n(\gamma - 1)}. \end{aligned}$$

Then

$$\begin{aligned} {\left\{ \begin{array}{ll} \displaystyle F'(s) = 1 - \frac{\alpha }{n(\gamma - 1)}B_{n,\gamma ,\alpha } M^{\frac{\gamma (2n - \alpha ) - 2n}{n(\gamma -1)}}s^\frac{\alpha - n(\gamma - 1)}{n(\gamma - 1)}, \\ \displaystyle F''(s) = - \frac{\alpha (\alpha - n(\gamma -1))}{n^2(\gamma - 1)^2} B_{n,\gamma ,\alpha }M^{\frac{\gamma (2n - \alpha ) - 2n}{n(\gamma -1)}}s^\frac{\alpha - 2n(\gamma - 1)}{n(\gamma - 1)}. \end{array}\right. } \end{aligned}$$

which, for $$\gamma \in (\frac{2n}{2n - \alpha },\frac{n + \alpha }{n})$$, implies $F^{\prime \prime }\left(s\right)<0$ for any s>0. This implies that *F*(*s*) is concave for s>0. Denote

$$\begin{aligned} s_* = \Big ( \frac{\alpha B_{n,\gamma ,\alpha }}{n(\gamma - 1)} \Big )^\frac{n(\gamma - 1)}{n(\gamma - 1) - \alpha } M^\frac{\gamma (2n - \alpha ) - 2n}{n(\gamma - 1) - \alpha }, \end{aligned}$$

the critical point of *F*(*s*) (*i.e.,* $$F'(s_*) = 0$$). Since $F^{\prime \prime }\left(s\right)<0$ for s>0, we see that the maximum of *F*(*s*) for s≥0 is given by

$$\begin{aligned} F(s_*) = \frac{\alpha - n(\gamma - 1)}{\alpha } s_* > 0. \end{aligned}$$

Thus, using $M < M^{\varepsilon ,b,\alpha }_\textrm{c}(\gamma )$, (2.17), and $\frac{\gamma (2n - \alpha ) - 2n}{n(\gamma - 1) - \alpha } < 0$ for $\gamma \in (\frac{2n}{2n - \alpha },\frac{n + \alpha }{n})$, we have

3.16 $$\begin{aligned} F(s_*) > \frac{\alpha - n(\gamma - 1)}{\alpha } \Big (\frac{\alpha B_{n,\gamma ,\alpha }}{n(\gamma - 1)} \Big )^\frac{n(\gamma - 1)}{n(\gamma - 1) - \alpha }M^{\varepsilon ,b,\alpha }_\textrm{c}(\gamma )^\frac{\gamma (2n - \alpha ) - 2n}{n(\gamma - 1) - \alpha } = E^{\varepsilon ,b}_0, \end{aligned}$$

and

3.17 $$\begin{aligned} s_* = \frac{\alpha }{\alpha - n(\gamma - 1)}F(s_*)> \frac{\alpha }{\alpha - n(\gamma - 1)}E^{\varepsilon ,b}_0 > 2E^{\varepsilon ,b}_0. \end{aligned}$$

Now, using (3.13) and (3.15)–(3.17), we obtain

3.18 $$\begin{aligned}&F( \int ^{b(t)}_a \rho e(\rho ) r^{n-1} {\, \mathrm d}r) \le E^{\varepsilon ,b}_0 < F(s_*),\end{aligned}$$

3.19 $$\begin{aligned}&\quad \int ^{b}_a \rho _0 e(\rho _0) r^{n-1} {\, \mathrm d}r \le E^{\varepsilon ,b}_0 < s_*. \end{aligned}$$

Thus, since $t \mapsto \int ^{b(t)}_a \rho e(\rho ) r^{n-1} {\, \mathrm d}r$ is continuous with respect to *t*, we must have

$$\begin{aligned} \int ^{b(t)}_a \rho e(\rho ) r^{n-1} {\, \mathrm d}r < s_*, \end{aligned}$$

as otherwise we can apply the intermediate value theorem to derive a contradiction to (3.18). This implies

3.20 $$\begin{aligned}&F(\int ^{b(t)}_a \rho e(\rho ) r^{n-1} {\, \mathrm d}r) \nonumber \\&\quad \ge \Big (1 - B_{n,\gamma ,\alpha } M^{\frac{\gamma (2n - \alpha ) - 2n}{n(\gamma -1)}}s_*^\frac{\alpha - n(\gamma - 1)}{n(\gamma - 1)}\Big ) \int ^{b(t)}_a \rho e(\rho ) r^{n-1} {\, \mathrm d}r \nonumber \\&\quad = \frac{\alpha - n(\gamma -1)}{\alpha }\int ^{b(t)}_a \rho e(\rho ) r^{n-1} {\, \mathrm d}r. \end{aligned}$$

Therefore, using (3.13), (3.15), and (3.20), we derive (3.6).

5. For Case 4, we first consider α=0. For $\boldsymbol{x},\boldsymbol{y} \in \mathbb {R}^n$ with $|\boldsymbol{x} - \boldsymbol{y}| \ge 1$, we know that $\log (|\boldsymbol{x}| + |\boldsymbol{y}|) \ge \log (|\boldsymbol{x} - \boldsymbol{y}|) \ge 0$. Then, if $|\boldsymbol{y}| \ge |\boldsymbol{x}|$,

$$ \log (|\boldsymbol{x}| + |\boldsymbol{y}|) = \frac{1}{2} \log ((|\boldsymbol{x}| + |\boldsymbol{y}|)^2) \le \frac{1}{2} \log (4 + 4 |\boldsymbol{y}|^2) = \log 2 + \frac{1}{2} \log (1 + |\boldsymbol{y}|^2). $$

If $$|\boldsymbol{x}| \ge |\boldsymbol{y}|$$, then

$$ \log (|\boldsymbol{x}| + |\boldsymbol{y}|) \le \log 2 + \frac{1}{2} \log (1 + |\boldsymbol{x}|^2). $$

Thus, we obtain that, for $\boldsymbol{x}, \boldsymbol{y} \in \mathbb {R}^n$ such that $|\boldsymbol{x} - \boldsymbol{y}| \ge 1$,

3.21 $$\begin{aligned} \log (|\boldsymbol{x}| + |\boldsymbol{y}|) \le \log 4 + \frac{1}{2} \log (1 + |\boldsymbol{x}|^2) + \frac{1}{2} \log (1 + |\boldsymbol{y}|^2). \end{aligned}$$

Then, by (3.21) and the Hölder inequality, we have

3.22 $$\begin{aligned}&\bigg | \int ^{b(t)}_a \rho (\Phi _0 * \rho ) r^{n-1} {\, \mathrm d}r \bigg |\nonumber \\&\quad = \frac{1}{\omega _n} \bigg |\int _{\mathbb {R}^n} \int _{\mathbb {R}^n} \rho (\boldsymbol{x}) \log (|\boldsymbol{x} - \boldsymbol{y}|) \rho (\boldsymbol{y}) {\, \mathrm d}\boldsymbol{x} {\, \mathrm d}\boldsymbol{y} \bigg |\nonumber \\&\quad \le \frac{1}{\omega _n} \int _{\mathbb {R}^n} \rho (\boldsymbol{y}) \Big ( \int _{B_1(\boldsymbol{y})} \rho (\boldsymbol{x}) \log (|\boldsymbol{x} - \boldsymbol{y}|^{-1}) {\, \mathrm d}\boldsymbol{x} + \int _{B_1(\boldsymbol{y})^c} \rho (\boldsymbol{x}) \log (|\boldsymbol{x} - \boldsymbol{y}|) {\, \mathrm d}\boldsymbol{x} \Big ) {\, \mathrm d}\boldsymbol{y} \nonumber \\&\quad \le \frac{1}{\omega _n} \int _{\mathbb {R}^n} \rho (\boldsymbol{y}) \bigg ( \Vert \rho \Vert _{L^\gamma (B_1(\boldsymbol{y}))} \Vert \log (|\cdot |) \Vert _{L^\frac{\gamma }{\gamma - 1}(B_1)} \nonumber \\&\qquad \qquad \qquad \qquad \qquad + \int _{|\boldsymbol{x} - \boldsymbol{y}| \ge 1} \rho (\boldsymbol{x}) \Big (\log 4 + \frac{1}{2} \log (1 + |\boldsymbol{x}|^2) + \frac{1}{2} \log (1 + |\boldsymbol{y}|^2) \Big ) {\, \mathrm d}\boldsymbol{x} \bigg ){\, \mathrm d}\boldsymbol{y}\nonumber \\&\quad \le C(M) \Big (1 + \Vert \rho \Vert _{L^\gamma (\Omega _t)} + \int _{\Omega _t} \rho (\boldsymbol{x}) \log (1+|\boldsymbol{x}|^2) {\, \mathrm d}\boldsymbol{x}\Big ) \nonumber \\&\quad \le \frac{1}{2} \int ^{b(t)}_a \rho e(\rho ) r^{n-1} {\, \mathrm d}r + C(M) \Big (1 + \int _a^{b(t)} \rho (r) \log (1+r^2) r^{n-1} {\, \mathrm d}r \Big ), \end{aligned}$$

where we have applied the Young inequality in the final line.

6. For $\alpha \in (-1,0)$, we see that, for $\boldsymbol{x},\boldsymbol{y} \in \mathbb {R}^n$,

3.23 $$\begin{aligned} |\Phi _\alpha (\boldsymbol{x} - \boldsymbol{y})| \le \frac{2^{-\alpha }}{-\alpha } \big (k_\alpha (1 + |\boldsymbol{x}|^2) + k_\alpha (1 + |\boldsymbol{y}|^2) \big ). \end{aligned}$$

Thus, employing a similar method to (3.22), we use (3.23) to obtain

3.24 $$\begin{aligned} \left| \int ^{b(t)}_a \rho (\Phi _\alpha * \rho ) r^{n-1} {\, \mathrm d}r \right| \le \frac{2^{1 - \alpha } M}{- \alpha } \int ^{b(t)}_a \rho k_\alpha (1 + r^2) r^{n-1} {\, \mathrm d}r. \end{aligned}$$

Considering the latter integral in (3.22) and (3.24), but in generality for $\alpha \in (-1,0]$, from (2.10) and integration by parts, we obtain

3.25 $$\begin{aligned}&\frac{\textrm{d}}{\textrm{d}t} \int ^{b(t)}_a \rho k_\alpha (1 + r^2) r^{n-1} {\, \mathrm d}r\nonumber \\&\quad = \big (\rho u k_\alpha (1 + r^2) r \big )(t,b(t)) + \int ^{b(t)}_a \rho _t k_\alpha (1 + r^2) r^{n-1} {\, \mathrm d}r\nonumber \\&\quad = \big (\rho u k_\alpha (1 + r^2) r \big )(t,b(t)) - \int ^{b(t)}_a k_\alpha (1 + r^2) ( \rho u r^{n-1})_r {\, \mathrm d}r\nonumber \\&\quad = \int ^{b(t)}_a (k_\alpha (1 + r^2))_r \rho u r^{n-1} {\, \mathrm d}r\nonumber \\&\quad \le \int ^{b(t)}_a \rho |u| r^{n-1} {\, \mathrm d}r \nonumber \\&\quad \le \frac{1}{2} \int ^{b(t)}_a \rho (u^2 + 1) r^{n-1} {\, \mathrm d}r\nonumber \\&\quad = \frac{1}{2} \int ^{b(t)}_a \rho u^2 r^{n-1} {\, \mathrm d}r + \frac{M}{2 \omega _n}. \end{aligned}$$

Integrating (3.25) in *t*, we see that, for $\alpha \in (-1,0]$,

3.26 $$\begin{aligned} \int ^{b(t)}_a \rho k_\alpha (1 + r^2) r^{n-1} {\, \mathrm d}r \le \int ^{b}_a \rho _0 k_\alpha (1 + r^2) r^{n-1} {\, \mathrm d}r + \frac{Mt}{2\omega _n} + \frac{1}{2} \int ^t_0 \int ^{b(s)}_a \rho u^2 r^{n-1} {\, \mathrm d}r {\, \mathrm d}s. \end{aligned}$$

Hence, combining (3.22), (3.24), and (3.26), we obtain

3.27 $$\begin{aligned}&\bigg | \int ^{b(t)}_a \rho (\Phi _\alpha * \rho ) r^{n-1} {\, \mathrm d}r \bigg | + \int ^{b(t)}_a \rho k_\alpha (1 + r^2) r^{n-1} {\, \mathrm d}r \nonumber \\&\quad \le \frac{1}{2} \int ^{b(t)}_a \rho e(\rho ) r^{n-1} {\, \mathrm d}r\nonumber \\&\qquad + C(M,T) \Big (1 + \int ^{b}_a \rho _0 k_\alpha (1 + r^2) r^{n-1} {\, \mathrm d}r + \int ^t_0 \int ^{b(s)}_a \rho u^2 r^{n-1} {\, \mathrm d}r {\, \mathrm d}s \Big ). \end{aligned}$$

Combining (3.13) and (3.27), we obtain that, for $\alpha \in (-1,0]$,

3.28 $$\begin{aligned}&\frac{1}{2} \int ^{b(t)}_a \rho \big (u^2 + e(\rho ) + k_\alpha (1 + r^2) \big )r^{n-1} {\, \mathrm d}r + \varepsilon \int ^t_0\int ^{b(s)}_a \rho \big (u_r^2 + \frac{u^2}{r^2} \big ) r^{n-1} {\, \mathrm d}r {\, \mathrm d}s\nonumber \\&\quad \quad + \varepsilon \int ^t_0 (\rho u^2)(s,b(s)) b(s)^{n-2} {\, \mathrm d}s\nonumber \\&\quad \le \int ^b_a \rho _0 \Big (\frac{1}{2}u_0^2 + e(\rho _0) + \frac{\kappa }{2} (\Phi _\alpha * \rho _0) \Big )r^{n-1} {\, \mathrm d}r\nonumber \\&\quad \quad + C(M,T) \bigg (1 + \int ^{b}_a \rho _0 k_\alpha (1 + r^2) r^{n-1} {\, \mathrm d}r + \int ^t_0 \int ^{b(s)}_a \rho u^2 r^{n-1} {\, \mathrm d}r {\, \mathrm d}s\bigg ). \end{aligned}$$

Applying the Grönwall inequality on (3.28) and then using (B.10), we conclude

$$\begin{aligned}&\frac{1}{2} \int ^{b(t)}_a \rho \big (u^2 + e(\rho ) + k_\alpha (1 + r^2) \big )r^{n-1} {\, \mathrm d}r + \varepsilon \int ^t_0\int ^{b(s)}_a \rho \big (u_r^2 + \frac{u^2}{r^2} \big ) r^{n-1} {\, \mathrm d}r {\, \mathrm d}s \\&\quad \quad + \varepsilon \int ^t_0 (\rho u^2)(s,b(s)) b(s)^{n-2} {\, \mathrm d}s \\&\quad \le C(M,T) \bigg ( 1 + \int ^b_a \rho _0 \Big (\frac{1}{2}u_0^2 + e(\rho _0) + \frac{\kappa }{2} (\Phi _\alpha *\rho _0) + k_\alpha (1 + r^2) \Big )r^{n-1} {\, \mathrm d}r \bigg ) \\&\quad \le C(M,E^{\varepsilon ,b}_0,T). \end{aligned}$$

□

#### Remark 3.5

Adding $\frac{\kappa M^2}{2\alpha } = \frac{\kappa }{2} \int ^{b(t)}_a \rho (\frac{1}{\alpha } * \rho )r^{n-1} {\, \mathrm d}r = \frac{\kappa }{2} \int ^{b}_a \rho _0 (\frac{1}{\alpha } * \rho _0)r^{n-1} {\, \mathrm d}r$ to both sides of (3.5) yields

3.29 $$\begin{aligned}&\int ^{b(t)}_a \rho \Big (\frac{1}{2}u^2 + e(\rho ) + \frac{\kappa }{2} \big ((\frac{1}{\alpha } + \Phi _\alpha \big ) * \rho \big ) \Big )r^{n-1} {\, \mathrm d}r \nonumber \\&\qquad + \varepsilon \int ^t_0\int ^{b(s)}_a \rho \Big (u_r^2 + (n-1)\frac{u^2}{r^2} \Big ) r^{n-1} {\, \mathrm d}r{\, \mathrm d}s \nonumber \\&\quad \quad + (n-1)\varepsilon \int ^t_0 (\rho u^2)(s,b(s))b(s)^{n-2} {\, \mathrm d}s \nonumber \\&\quad = \int ^b_a \rho _0 \Big (\frac{1}{2}u_0^2 + e(\rho _0) + \frac{\kappa }{2} \big ((\frac{1}{\alpha } + \Phi _\alpha ) * \rho _0 \big ) \Big )r^{n-1} {\, \mathrm d}r. \end{aligned}$$

We can formally see that, using (1.3), as $\alpha \rightarrow 0$, (3.29) converges to

$$\begin{aligned} \begin{aligned}&\int ^{b(t)}_a \rho \Big (\frac{1}{2}u^2 + e(\rho ) + \frac{\kappa }{2} ( \Phi _0 * \rho ) \Big )r^{n-1} {\, \mathrm d}r + \varepsilon \int ^t_0\int ^{b(s)}_a \rho \Big (u_r^2 + (n-1)\frac{u^2}{r^2} \Big ) r^{n-1} {\, \mathrm d}r{\, \mathrm d}s \\&\quad \quad + (n-1)\varepsilon \int ^t_0 (\rho u^2)(s,b(s))b(s)^{n-2} {\, \mathrm d}s \\&\quad = \int ^b_a \rho _0 \Big (\frac{1}{2}u_0^2 + e(\rho _0) + \frac{\kappa }{2} (\Phi _0 * \rho _0) \Big )r^{n-1} {\, \mathrm d}r. \end{aligned} \end{aligned}$$

#### Corollary 3.6

(Uniform Estimates). Under the conditions of Lemma 3.4, the following uniform estimates in (ε,b) (for ε small enough and *b* large enough) hold:

Case 1: κ=-1 with γ>1 for $\alpha \in (0,n-2]$ or $\gamma > \frac{2n}{2n - \alpha }$ for $\alpha \in (n-2,n-1)$,

3.30 $$\begin{aligned} \int _{\mathbb {R}^n} \rho \big (1 + u^2 + \rho ^{\gamma - 1} - \Phi _\alpha * \rho \big ) {\, \mathrm d}\boldsymbol{x} \le C(M,E_0); \end{aligned}$$

Case 2: $\alpha \in (0,n-1)$ and κ=1 with $\gamma > \frac{2n}{2n - \alpha }$,

3.31 $$\begin{aligned} \int _{\mathbb {R}^n} \rho \big (1 + u^2 + \rho ^{\gamma -1} \big ) {\, \mathrm d}\boldsymbol{x} \le C(M,E_0); \end{aligned}$$

Case 3: $\alpha \in (-1,0]$ and $\kappa \in \{-1,1\}$ with γ>1,

3.32 $$\begin{aligned} \int _{\mathbb {R}^n} \rho \big (1 + u^2 + \rho ^{\gamma - 1} + k_\alpha (1 + |\boldsymbol{x}|^2) \big ) {\, \mathrm d}\boldsymbol{x} \le C(M,E_0,T), \end{aligned}$$

where we have used the convention that ρ and *u* are zero outside of $\Omega _t$. In particular, the following uniform estimates in (ε,b) (for ε small enough and *b* large enough) hold: For Cases 1–2,

$$\begin{aligned} \Vert (\Phi _\alpha *\rho )(t)\Vert _{L^{\frac{2n}{\alpha }}(\mathbb {R}^n)} +\Vert (\nabla \Phi _\alpha *\rho )(t)\Vert _{L^\frac{2n}{\alpha + 2}(\mathbb {R}^n)} \le C(M,E_0); \end{aligned}$$

and for Case 3, for any $K \Subset \mathbb {R}^n$, there exists $C(M,E_0,T,K)>0$ such that

$$\begin{aligned} \Vert (\Phi _\alpha *\rho )(t)\Vert _{L^\infty (K)} +\Vert (\nabla \Phi _\alpha *\rho )(t)\Vert _{L^\frac{2n}{\alpha + 2}(K)} \le C(M,E_0,T,K). \end{aligned}$$

#### Proof

Estimates (3.30)–(3.32) follow from Lemma 3.4 and the fact that $\int ^{b(t)}_a \rho r^{n-1} {\, \mathrm d}r = \frac{M}{\omega _n}$, $e(\rho ) = \frac{a_0}{\gamma - 1} \rho ^{\gamma - 1}$, and $E_0^{\varepsilon ,b} \rightarrow E_0$ as $b \rightarrow \infty$ and $\varepsilon \rightarrow 0$ due to Lemma B.8.

For the second part of the corollary, for Case 2 or κ=-1 and $\gamma > \frac{2n}{2n - \alpha }$, by the HLS inequality in Lemma A.2 and interpolation, we have

$$\begin{aligned}&\Vert (\Phi _\alpha *\rho )(t)\Vert _{L^{\frac{2n}{\alpha }}(\mathbb {R}^n)} \le \frac{C_{n,\alpha }}{\alpha } \Vert \rho (t)\Vert _{L^1(\mathbb {R}^n)}^\frac{\gamma (2n - \alpha ) - 2n}{n(\gamma - 1)} \Vert \rho (t)\Vert _{L^\gamma (\mathbb {R}^n)}^\frac{\alpha \gamma }{n(\gamma - 1)} \le C(M,E_0), \\&\Vert (\nabla \Phi _\alpha *\rho )(t)\Vert _{L^{\frac{2n}{\alpha + 2}}(\mathbb {R}^n)} \le C_{n,\alpha + 1} \Vert \rho (t)\Vert _{L^1(\mathbb {R}^n)}^\frac{\gamma (2n - \alpha ) - 2n}{n(\gamma - 1)} \Vert \rho (t)\Vert _{L^\gamma (\mathbb {R}^n)}^\frac{\alpha \gamma }{n(\gamma - 1)} \le C(M,E_0), \end{aligned}$$

where the final inequalities follow from the first part of this corollary.

For Case 1, we use the Riesz composition formula in Theorem A.5, the HLS inequality in Lemma A.2, and the Tonelli theorem to obtain

$$\begin{aligned} \Vert \Phi _\alpha * \rho \Vert _{L^\frac{2n}{\alpha }(\mathbb {R}^n)}&\quad = \frac{1}{\alpha }\big \Vert |\cdot |^{-\alpha } * \rho \big \Vert _{L^\frac{2n}{\alpha }(\mathbb {R}^n)} \\&\quad = \frac{1}{\alpha \kappa _{\frac{n-\alpha }{2},\frac{n-\alpha }{2}}} \Big \Vert |\cdot |^{-\frac{n+\alpha }{2}} * \big (|\cdot |^{-\frac{n+\alpha }{2}} * \rho \big )\Big \Vert _{L^\frac{2n}{\alpha }(\mathbb {R}^n)} \\&\quad \le \frac{C_{n,\frac{n+\alpha }{2}}}{\alpha \kappa _{\frac{n-\alpha }{2},\frac{n-\alpha }{2}}} \Big \Vert |\cdot |^{-\frac{n+\alpha }{2}} * \rho \Big \Vert _{L^2(\mathbb {R}^n)} \\&\quad = \frac{C_{n,\frac{n+\alpha }{2}}}{\alpha \kappa _{\frac{n-\alpha }{2},\frac{n-\alpha }{2}}} \int _{\mathbb {R}^n} \rho \,\Big ( |\cdot |^{-\frac{n+\alpha }{2}}* \big (|\cdot |^{-\frac{n+\alpha }{2}} * \rho \big ) \Big ) {\, \mathrm d}\boldsymbol{x} \\&\quad = - C_{n,\frac{n+\alpha }{2}} \int _{\mathbb {R}^n} \rho ( \Phi _\alpha * \rho ) {\, \mathrm d}\boldsymbol{x} \\&\quad \le C(M,E_0), \end{aligned}$$

and, for $$\alpha \in (0,n-2)$$,

3.33 $$\begin{aligned} \Vert \nabla \Phi _\alpha * \rho \Vert _{L^\frac{2n}{\alpha + 2}(\mathbb {R}^n)}&\quad \le \frac{1}{\alpha }\big \Vert |\cdot |^{-(\alpha + 1)} * \rho \big \Vert _{L^\frac{2n}{\alpha + 2}(\mathbb {R}^n)}\nonumber \\&\quad = \frac{1}{\alpha \kappa _{\frac{n-\alpha }{2} - 1,\frac{n-\alpha }{2}}} \Big \Vert |\cdot |^{-\left( 1 + \frac{n+\alpha }{2} \right) } * \big (|\cdot |^{-\frac{n+\alpha }{2}} * \rho \big ) \Big \Vert _{L^\frac{2n}{\alpha + 2}(\mathbb {R}^n)} \nonumber \\&\quad \le \frac{C_{n,\frac{n+\alpha }{2} + 1}}{\alpha \kappa _{\frac{n-\alpha }{2} - 1,\frac{n-\alpha }{2}}} \Big \Vert |\cdot |^{-\frac{n+\alpha }{2}} * \rho \Big \Vert _{L^2(\mathbb {R}^n)}, \end{aligned}$$

where $\kappa _{\alpha ,\beta }$ is defined in (A.4).

For α=n-2, it follows directly that

3.34 $$\begin{aligned} \Vert \Phi _\alpha * \rho \Vert _{L^\frac{2n}{\alpha }(\mathbb {R}^n)}&\le \Big \Vert |\cdot |^{-\frac{n+\alpha }{2}} * \rho \Big \Vert _{L^2(\mathbb {R}^n)}. \end{aligned}$$

Now, applying Theorem A.5 and the Tonelli theorem, we have

3.35 $$\begin{aligned} \Big \Vert |\cdot |^{-\frac{n+\alpha }{2}} * \rho \Big \Vert _{L^2(\mathbb {R}^n)} ^{2}&= \int _{\mathbb {R}^n} \rho \,\Big ( |\cdot |^{-\frac{n+\alpha }{2}} * \big (|\cdot |^{-\frac{n+\alpha }{2}} * \rho \big ) \Big ) {\, \mathrm d}\boldsymbol{x}\nonumber \\&= - \kappa _{\frac{n-\alpha }{2},\frac{n-\alpha }{2}}\int _{\mathbb {R}^n} \rho (\Phi _\alpha * \rho ) {\, \mathrm d}\boldsymbol{x}\nonumber \\&\le C(M,E_0). \end{aligned}$$

Tying together (3.33)–(3.35), we obtain that, for κ=-1 and $\alpha \in (0,n-2]$,

$$\begin{aligned} \Vert \nabla \Phi _\alpha * \rho \Vert _{L^\frac{2n}{\alpha + 2}(\mathbb {R}^n)} \le C(M,E_0). \end{aligned}$$

For α=0, for simplicity, we may assume that $K = \overline{B_D(\textbf{0})}$ for some D>0, where $B_D(\textbf{0})$ denotes the ball with origin at $\textbf{0}$ and radius *D*. Notice that, for any $\boldsymbol{x} \in K$ and $\boldsymbol{y} \in \mathbb {R}^n$ with $|\boldsymbol{x} - \boldsymbol{y}| \ge 1$, $\log (|\boldsymbol{x}| + |\boldsymbol{y}|) \ge \log (|\boldsymbol{x} - \boldsymbol{y}|) \ge 0$. Then, if $\boldsymbol{y} \in K$,

$$ \log (|\boldsymbol{x}| + |\boldsymbol{y}|) \le \log (1 + 2D). $$

If $$\boldsymbol{y} \in \mathbb {R}^n \setminus K$$, then

$$ \log (|\boldsymbol{x}| + |\boldsymbol{y}|) = \frac{1}{2} \log ((|\boldsymbol{x}| + |\boldsymbol{y}|)^2) \le \frac{1}{2} \log (4 + 4 |\boldsymbol{y}|^2) = \log 2 + \frac{1}{2} \log (1 + |\boldsymbol{y}|^2). $$

Thus, for $\boldsymbol{y} \in \mathbb {R}^n$, we have

3.36 $$\begin{aligned} \log (|\boldsymbol{x}| + |\boldsymbol{y}|) \le \log (1 + 2D) + \log 2 + \frac{1}{2} \log (1 + |\boldsymbol{y}|^2) = \log (2 + 4D) + \frac{1}{2} \log (1 + |\boldsymbol{y}|^2). \end{aligned}$$

Then, for $\boldsymbol{x} \in K$, we apply the Hölder inequality, (3.36), and (3.7) to obtain

3.37 $$\begin{aligned}&|(\Phi _0 * \rho )(\boldsymbol{x})| \nonumber \\&\quad \le \int _{B_1(\boldsymbol{x})} \log (|\boldsymbol{x} - \boldsymbol{y}|^{-1}) \rho (\boldsymbol{y}) {\, \mathrm d}\boldsymbol{y} + \int _{B_1(\boldsymbol{x})^c} \log (|\boldsymbol{x} - \boldsymbol{y}|) \rho (\boldsymbol{y}) {\, \mathrm d}\boldsymbol{y}\nonumber \\&\quad \le \Vert \log (|\cdot |) \Vert _{L^\frac{\gamma }{\gamma - 1}(B_1)} \Vert \rho \Vert _{L^\gamma (B_1(\boldsymbol{x}))} + \int _{B_1(\boldsymbol{x})^c} \big (\log (2 + 4D) + \frac{1}{2} \log (1 + |\boldsymbol{y}|^2) \big ) \rho (\boldsymbol{y}) {\, \mathrm d}\boldsymbol{y}\nonumber \\&\quad \le C \Vert \rho \Vert _{L^\gamma (B_1(\boldsymbol{x}))} + \log (2 + 4D) \Vert \rho \Vert _{L^1(B_1(\boldsymbol{x})^c)} + \frac{1}{2} \int _{B_1(\boldsymbol{x})^c} \log (1 +|\boldsymbol{y}|^2)\rho (\boldsymbol{y}) {\, \mathrm d}\boldsymbol{y} \nonumber \\&\quad \le C(K,M,E_0,T). \end{aligned}$$

Therefore, we have

$$ \Vert \Phi _0 * \rho \Vert _{L^\infty (K)} \le C(K,M,E_0,T). $$

For $\alpha \in (-1,0)$, we assume again that $K = \overline{B_D}$ for some D>0. Using (3.23), we obtain that, for any $\boldsymbol{x} \in K$ and $y \in \mathbb {R}^n$,

3.38 $$\begin{aligned} |\Phi _\alpha (\boldsymbol{x} - \boldsymbol{y})| \le \frac{2^{-\alpha }}{-\alpha } \left( k_\alpha (1 + D^2) + k_\alpha (1 + |\boldsymbol{y}|^2) \right) . \end{aligned}$$

Thus, similar to (3.37), using (3.7) and (3.38), we have

$$\begin{aligned} \begin{aligned} |(\Phi _\alpha * \rho )(\boldsymbol{x})|&\le \frac{2^{-\alpha }}{-\alpha } \int _{\mathbb {R}^n} \left( k_\alpha (1 + D^2) + k_\alpha (1 + |\boldsymbol{y}|^2) \right) \rho (\boldsymbol{y}) {\, \mathrm d}\boldsymbol{y} \\&\le C(K,M,E_0,T). \end{aligned} \end{aligned}$$

Then we obtain

$$ \Vert \Phi _\alpha * \rho \Vert _{L^\infty (K)} \le C(K,M,E_0,T). $$

For $\alpha \in (-1,0]$, take $1< p < \min \{\frac{2n}{\alpha + 2},\gamma \}$. Applying the HLS inequality in Lemma A.2, interpolation, and (3.7), we have

$$\begin{aligned}&\Vert \nabla \Phi _\alpha * \rho \Vert _{L^\frac{2n}{\alpha + 2}(K)} \\&\quad \le \Vert (|\cdot |^{-(\alpha + 1)} 1\!\!1_{B_1}) * \rho \Vert _{L^\frac{2n}{\alpha + 2}(K)} + \Vert (|\cdot |^{-(\alpha + 1)} 1\!\!1_{B_1^c}) * \rho \Vert _{L^\frac{2n}{\alpha + 2}(K)} \\&\quad \le \Vert (|\cdot |^{-(1 + \frac{\alpha }{2} + n(1-\frac{1}{p}))}) * \rho \Vert _{L^\frac{2n}{\alpha + 2}(K)} + \Vert 1\!\!1_{B_1^c} * \rho \Vert _{L^\frac{2n}{\alpha + 2}(K)} \\&\quad \le C_{n,1 + \frac{\alpha }{2}+ n(1-\frac{1}{p})} \Vert \rho \Vert _{L^p(\mathbb {R}^n)} + |K|^{\frac{\alpha + 2}{2n}} \Vert \rho \Vert _{L^1(\mathbb {R}^n)} \\&\quad \le C_{n,1 + \frac{\alpha }{2} + n(1-\frac{1}{p})} \Vert \rho \Vert _{L^1(\mathbb {R}^n)}^{\frac{\gamma - p}{p(\gamma - 1)}} \Vert \rho \Vert _{L^\gamma (\mathbb {R}^n)}^{\frac{\gamma (p-1)}{p(\gamma - 1)}} + |K|^{\frac{\alpha + 2}{2n}} \Vert \rho \Vert _{L^1(\mathbb {R}^n)} \\&\quad \le C(K,M,E_0,T). \end{aligned}$$

□

#### Remark 3.7

It is still open for the repulsive case κ=-1 and $\alpha \in (n-2,n-1)$ without any restriction on γ, owing to the fact that the $L^\frac{2n}{\alpha + 2}$–norm of the derivative of the potential term cannot be bounded by the potential energy of the system.

### Expanding the domain

We need to ensure that, as $b \rightarrow \infty$, our domain $\Omega ^T$ converges to the whole space $\mathbb {R}^n$.

#### Lemma 3.8

(Expanding of Domain $\Omega ^T$). Given T>0 and $\varepsilon \in (0,\varepsilon _0]$, there exists a positive constant $C_1(M,E_0,T,\varepsilon )>0$ such that, if $b\ge \max \{C_1(M,E_0,T,\varepsilon ),\mathfrak {B}(\varepsilon )\}$ (as defined in Lemma B.8),

3.39 $$\begin{aligned} b(t)\ge \frac{b}{2}\qquad \,\, \text {for } t\in [0,T]. \end{aligned}$$

This lemma can be achieved by following the same argument as in [26, Lemma 3.4]. Therefore, as $b \rightarrow \infty$, $\lim _{b \rightarrow \infty } \inf _{t \in [0,T]} b(t) = \infty$, as we desire.

### BD-type entropy

We now need to make uniform estimates on the derivatives of the density in order to apply the Aubin-Lions lemma to prove the convergence of $(\rho ^{\varepsilon ,b}, \rho ^{\varepsilon ,b}u^{\varepsilon ,b})$ as $b \rightarrow \infty$ to global weak solutions $(\rho ^\varepsilon , \rho ^\varepsilon u^\varepsilon )$ of (1.6). We do so by proving a BD entropy bound, taking advantage of the fact that our viscosity terms satisfy $\lambda (\rho ) = \rho \mu '(\rho ) - \mu (\rho )$. We have two different BD-type estimates: The first is to follow the same approach as in [26] for $\alpha \in (-1,n-2]$ due to the inability to integrate by parts rigorously for α>n-2, as $\Phi _\alpha * \rho$ is only twice differentiable for $\alpha \in (-1,n-2]$. This approach fails to provide a suitable estimate in the attractive case κ=1, due to the lack of the control of the boundary term $a^{n-1} (\rho (\Phi _\alpha * \rho )_r)(t,a)$ as we have no *a priori* estimate of the density at the lower boundary r=a except for (2.15). Moreover, this is insufficient, as we require this to be a uniform bound in *b*, which is not the case. Although, for α=n-2, we have no issue, as we can show that the boundary term disappears. However, in the repulsive case κ=-1, we can show that the boundary term has favourable sign and can thus be controlled.

#### Lemma 3.9

(BD-Type Entropy Estimate I). Under the conditions of Lemma 3.4, for κ=-1 with $\alpha \in (-1,n-2]$ or κ=1 with α=n-2, any given T>0, $\varepsilon \in (0,\varepsilon _0]$, and $b \ge \max \{C_1(M,E_0,T,\varepsilon ), \mathfrak {B}(\varepsilon )\}$ (as defined in Lemmas 3.8 and B.8), the following estimate holds:

$$\begin{aligned}&\frac{\varepsilon ^2}{4}\int _a^{b(t)} \big |(\sqrt{\rho (t,r)})_r\big |^2\,r^{n-1} {\, \mathrm d}r +\frac{4 a_0\varepsilon }{\gamma } \int _0^t\int _a^{b(s)} |(\rho ^{\frac{\gamma }{2}})_r|^2\,r^{n-1}{\, \mathrm d}r{\, \mathrm d}s\\&\quad \quad +\frac{1}{n}p(\rho (t,b(t)))b(t)^n +\frac{1}{n\varepsilon }\int _0^t p(\rho (s,b(s)))p'(\rho (s,b(s))) b(s)^n{\, \mathrm d}s \\&\quad \le C(E_0,M,T) \qquad \,\,\text {for all } t \in [0,T]. \end{aligned}$$

#### Proof

We divide the proof into four steps.

1. We first consider (2.10) in Lagrangian coordinates. Taking the derivative of the mass equation $(2.20)_1$ with respect to *x*, we have

$$\begin{aligned} \rho _{x \tau } = -\big (\rho ^2(r^{n-1}u)_x\big )_x. \end{aligned}$$

Plugging it into $(2.20)_2$ leads to

3.40 $$\begin{aligned} u_\tau + r^{n-1}p_x = -\varepsilon r^{n-1} \rho _{x \tau } - \varepsilon (n-1)r^{n-2}\rho _x u - \kappa \rho r^{n-1}(\Phi _\alpha * \rho )_x. \end{aligned}$$

Furthermore, (3.40) can be simplified to

3.41 $$\begin{aligned} (u + \varepsilon r^{n-1}\rho _x)_\tau + r^{n-1}p_x = - \kappa \rho r^{n-1}(\Phi _\alpha * \rho )_x. \end{aligned}$$

Now, multiplying (3.41) by $u + \varepsilon r^{n-1}\rho _x$ and integrating with respect to *x* over $[0,\frac{M}{\omega _n}]$, we obtain

3.42 $$\begin{aligned}&\frac{1}{2}\frac{\textrm{d}}{\textrm{d}\tau }\int _0^{\frac{M}{\omega _n}}(u+\varepsilon r^{n-1}\rho _x)^2 {\, \mathrm d}x +\varepsilon \int _0^{\frac{M}{\omega _n}}r^{2n-2}p(\rho )_x\rho _x{\, \mathrm d}x+\int _0^{\frac{M}{\omega _n}}r^{n-1}p(\rho )_x u{\, \mathrm d}x\nonumber \\&\quad = - \kappa \int ^{\frac{M}{\omega _n}}_0 u\rho r^{n-1}(\Phi _\alpha * \rho )_x {\, \mathrm d}x - \kappa \varepsilon \int ^{\frac{M}{\omega _n}}_0 \rho \rho _x r^{2(n-1)} (\Phi _\alpha * \rho )_x {\, \mathrm d}x. \end{aligned}$$

Using (3.11), we can rewrite (3.42) in the form:

3.43 $$\begin{aligned}&\frac{1}{2}\frac{\textrm{d}}{\textrm{d}\tau }\int _0^{\frac{M}{\omega _n}}(u+\varepsilon r^{n-1}\rho _x)^2 {\, \mathrm d}x +\varepsilon \int _0^{\frac{M}{\omega _n}}r^{2n-2}p(\rho )_x\rho _x{\, \mathrm d}x+\int _0^{\frac{M}{\omega _n}}r^{n-1}p(\rho )_x u{\, \mathrm d}x\nonumber \\&\quad = - \frac{\kappa }{2} \frac{\textrm{d}}{\textrm{d}\tau } \int ^{\frac{M}{\omega _n}}_0(\Phi _\alpha * \rho ) {\, \mathrm d}x - \kappa \varepsilon \int ^{\frac{M}{\omega _n}}_0 \rho \rho _x r^{2(n-1)} (\Phi _\alpha * \rho )_x {\, \mathrm d}x. \end{aligned}$$

Using $(2.20)_1$ and (2.12), we have

3.44 $$\begin{aligned} \rho _\tau (\tau ,\frac{M}{\omega _n}) = -(\rho ^2(r^{n-1}u)_x)(\tau ,\frac{M}{\omega _n}) = -\frac{a_0 \rho ^\gamma }{\varepsilon }(\tau ,\frac{M}{\omega _n}). \end{aligned}$$

Now, applying $(2.20)_1$, (2.18), and (3.44), we obtain

3.45 $$\begin{aligned}&\int _0^{\frac{M}{\omega _n}}p(\rho )_xu\,r^{n-1}{\, \mathrm d}x\nonumber \\&\quad =-\int _0^{\frac{M}{\omega _n}}p(\rho )(r^{n-1}u)_x {\, \mathrm d}x +( p(\rho )u r^{n-1})(\tau ,\frac{M}{\omega _n})\nonumber \\&\quad =\frac{\textrm{d}}{\textrm{d}\tau }\int _0^{\frac{M}{\omega _n}}e(\rho ){\, \mathrm d}x +( p(\rho )r^{n-1})(\tau ,\frac{M}{\omega _n})\, r_\tau (\tau ,\frac{M}{\omega _n})\nonumber \\&\quad =\frac{\textrm{d}}{\textrm{d}\tau }\int _0^{\frac{M}{\omega _n}}e(\rho ){\, \mathrm d}x +\Big (\frac{1}{n}p(\rho (\tau ,\frac{M}{\omega _n}))b(\tau )^n\Big )_\tau -\frac{1}{n}p'(\rho (\tau ,\frac{M}{\omega _n}))\rho _{\tau }(\tau ,\frac{M}{\omega _n})b(\tau )^n\nonumber \\&\quad =\frac{\textrm{d}}{\textrm{d}\tau }\int _0^{\frac{M}{\omega _n}}e(\rho ) {\, \mathrm d}x +\Big (\frac{1}{n}p(\rho (\tau ,\frac{M}{\omega _n}))b(\tau )^n\Big )_\tau + \frac{1}{n\varepsilon }p(\rho (\tau ,\frac{M}{\omega _n}))p'(\rho (\tau ,\frac{M}{\omega _n}))b(\tau )^n. \end{aligned}$$

Substituting (3.45) into (3.43), we have

3.46 $$\begin{aligned}&\frac{\textrm{d}}{\textrm{d}\tau }\Big \{\int _0^{\frac{M}{\omega _n}}\frac{1}{2}(u+\varepsilon r^{n-1}\rho _x)^2\,{\, \mathrm d}x +\int _0^{\frac{M}{\omega _n}}e(\rho )\,{\, \mathrm d}x + \frac{\kappa }{2}\int ^{\frac{M}{\omega _n}}_0\Phi _\alpha * \rho {\, \mathrm d}x\Big \} \nonumber \\&\quad \quad +\varepsilon \int _0^{\frac{M}{\omega _n}}p'(\rho )\rho _x^2\,r^{2n-2} {\, \mathrm d}x +\Big (\frac{1}{n}p(\rho (\tau ,\frac{M}{\omega _n}))b(\tau )^n\Big )_\tau \nonumber \\&\quad \quad +\frac{1}{n\varepsilon }p(\rho (\tau ,\frac{M}{\omega _n}))p'(\rho (\tau ,\frac{M}{\omega _n})) b(\tau )^n \nonumber \\&\quad = - \kappa \varepsilon \int ^{\frac{M}{\omega _n}}_0 \rho \rho _x r^{2(n-1)} (\Phi _\alpha * \rho )_x {\, \mathrm d}x\nonumber \\&\quad = - \kappa \varepsilon \left[ \rho ^2 r^{2(n-1)} (\Phi _\alpha * \rho )_x \right] ^\frac{M}{\omega _n}_0 + \kappa \varepsilon \int ^{\frac{M}{\omega _n}}_0 \rho (\rho r^{2(n-1)} (\Phi _\alpha * \rho )_x)_x {\, \mathrm d}x. \end{aligned}$$

2. Consider the last integral of (3.46). Using the HLS inequality in Lemma A.2, we obtain the estimate for $\alpha \in (-1,n-2]$:

$$\begin{aligned} {\begin{matrix} &  \int ^{\frac{M}{\omega _n}}_0 \rho \big (\rho r^{2(n-1)} (\Phi _\alpha * \rho )_x\big )_x {\, \mathrm d}x \\ &  = \frac{1}{\omega _n} \int _{\Omega _t} (\rho \Delta (\Phi _\alpha * \rho ))(t,\boldsymbol{y}) {\, \mathrm d}\boldsymbol{y} \\ &  \le \frac{1}{\omega _n} \Vert \rho \Vert _{L^{\frac{2n}{2n - (\alpha + 2)}}(\Omega _t)} \Vert \Delta (\Phi _\alpha * \rho ) \Vert _{L^\frac{2n}{\alpha + 2}(\mathbb {R}^n)} \\ &  = \frac{1}{\omega _n} \Vert \rho \Vert _{L^{\frac{2n}{2n - (\alpha + 2)}}(\Omega _t)} \big \Vert \, \Delta \Phi _\alpha * \rho \big \Vert _{L^\frac{2n}{\alpha + 2}(\mathbb {R}^n)} \\ &  \le C \Vert \rho \Vert _{L^{\frac{2n}{2n - (\alpha + 2)}}(\Omega _t)}^2. \end{matrix}} \end{aligned}$$

Case 1: κ=-1. We can show that the last term on the RHS of (3.46) is negative as

$$\begin{aligned} \Delta \Phi _\alpha = {\left\{ \begin{array}{ll} \frac{(n-2) - \alpha }{|\,\cdot \,|^{\alpha + 2}}\quad &  \text { for } \alpha \in (-1,n-2), \\ \omega _n \delta _0 &  \text { for } \alpha = n-2, \end{array}\right. } \end{aligned}$$

in the sense of distributions, where $\delta _0$ is the Dirac mass centred at 0. Then we have

3.47 $$\begin{aligned} \begin{aligned} \rho (\rho r^{2(n-1)} (\Phi _\alpha * \rho )_x)_x = \Delta (\Phi _\alpha * \rho )&= {\left\{ \begin{array}{ll} (n-2 - \alpha )(|\cdot |^{-(\alpha + 2)} * \rho ) & \text { for } \alpha \in (-1,n-2), \\ \omega _n \rho &  \text { for } \alpha = n-2 \end{array}\right. } \\&\ge 0. \end{aligned} \end{aligned}$$

Thus, we obtain

$$\begin{aligned} \begin{aligned}&\frac{\textrm{d}}{\textrm{d}\tau }\Big \{\int _0^{\frac{M}{\omega _n}}\frac{1}{2}(u+\varepsilon r^{n-1}\rho _x)^2\,{\, \mathrm d}x +\int _0^{\frac{M}{\omega _n}}e(\rho )\,{\, \mathrm d}x + \frac{\kappa }{2}\int ^{\frac{M}{\omega _n}}_0 \Phi _\alpha * \rho {\, \mathrm d}x\Big \}\\&\quad \quad +\varepsilon \int _0^{\frac{M}{\omega _n}}p'(\rho )\rho _x^2\,r^{2n-2} {\, \mathrm d}x +\Big (\frac{1}{n}p(\rho (\tau ,\frac{M}{\omega _n}))b(\tau )^n\Big )_\tau \\&\quad \quad +\frac{1}{n\varepsilon }p(\rho (\tau ,\frac{M}{\omega _n}))p'(\rho (\tau ,\frac{M}{\omega _n})) b(\tau )^n \\&\quad \le - \kappa \varepsilon \left[ \rho ^2 r^{2(n-1)} (\Phi _\alpha * \rho )_x \right] ^\frac{M}{\omega _n}_0. \end{aligned} \end{aligned}$$

Case 2: κ=1 and $\gamma \ge \frac{2n}{2n - \alpha - 2}$. Then we can control the integral by the internal energy:

$$\begin{aligned} \begin{aligned} \Vert \rho \Vert _{L^{\frac{2n}{2n - \alpha - 2}}(\Omega _t)}^2 \le C \bigg ( \int ^{b(t)}_a \rho \big (1 + e(\rho )\big )r^{n-1} {\, \mathrm d}r \bigg )^\frac{2n - \alpha - 2}{n}. \end{aligned} \end{aligned}$$

Case 3: κ=1 and $\gamma \in (\frac{2n}{2n - \alpha }, \frac{2n}{2n - \alpha -2})$. Applying the interpolation yields

$$\begin{aligned} \Vert \rho \Vert _{L^{\frac{2n}{2n -\alpha -2}}(\Omega _t)} = \Vert \rho \Vert _{L^{\frac{n\gamma }{n-2}}(\Omega _t)}^{\theta } \Vert \rho \Vert _{L^{\gamma }(\Omega _t)}^{1 - \theta } \qquad \text { with } \theta = \frac{2n + (\alpha + 2 - 2n)\gamma }{4}. \end{aligned}$$

This can be controlled as in [26].

3. Now, looking at the second-to-last term of (3.46), we have

$$\begin{aligned} \big [ \rho ^2 r^{2(n-1)} (\Phi _\alpha * \rho )_x \big ]^\frac{M}{\omega _n}_0 = \big [ \rho r^{n-1} (\Phi _\alpha * \rho )_r \big ]^{b(t)}_a. \end{aligned}$$

From (3.47), we see that $r \mapsto r^{n-1}(\Phi _\alpha * \rho )_r$ is increasing in *r*. Since $\lim _{r \searrow 0} r^{n-1}(\Phi _\alpha * \rho )_r = 0$, then $a^{n-1}(\rho (\Phi _\alpha * \rho )_r)(t,a) \ge 0$, owing to the fact that ρ(t,a)>0.

In the case that κ=-1, we can ignore this term as it has a negative sign.

In the case that α=n-2, we obtain

$$\begin{aligned} \begin{aligned} (r^{n-1}(\Phi _{n-2} * \rho )_r)_r = r^{n-1} \Delta (\Phi _{n-2} * \rho ) = \omega _n r^{n-1} \rho (t,r) = 0 \qquad \text { for all } r \in (0,a). \end{aligned} \end{aligned}$$

Hence, $r \mapsto r^{n-1}(\Phi _{n-2} * \rho )_r$ is constant in r∈(0,a). Since $\lim _{r \searrow 0} r^{n-1}(\Phi _{n-2} * \rho )_r = 0$, then $a^{n-1}(\rho (\Phi _{n-2} * \rho )_r)(t,a) = 0$.

4. We now focus on the other boundary term for $\alpha \in (-1,n-2)$ to obtain

3.48 $$\begin{aligned} \bigg | \int _{\mathbb {S}^{n-1}} \frac{b(s)\boldsymbol{e_1} - \eta \boldsymbol{y}}{|b(s)\boldsymbol{e_1} - \eta \boldsymbol{y}|^{\alpha + 2}} \cdot \boldsymbol{e_1} {\, \mathrm d}\sigma (\boldsymbol{y}) \bigg | \le \omega _n |b(s) - \eta |^{-(\alpha + 1)}. \end{aligned}$$

Employing (3.48), (3.39), (3.1), and $a = \frac{1}{b}$, we obtain that, for $\eta \in (a,b(s))$,

3.49 $$\begin{aligned} \bigg | \int _{\mathbb {S}^{n-1}} \frac{b(s)\boldsymbol{e_1} - \eta \boldsymbol{y}}{|b(s)\boldsymbol{e_1} - \eta \boldsymbol{y}|^{\alpha + 2}} \cdot \boldsymbol{e_1} {\, \mathrm d}\sigma (\boldsymbol{y}) \bigg |&\quad \le C \min \{|b(s) - \eta |^{-(\alpha + 1)}, \,(\eta b(s))^{-\frac{\alpha + 1}{2}} \} \nonumber \\&\quad \le C \min \{|b(s) - \eta |^{-(\alpha + 1)},\, 1 \} \le C. \end{aligned}$$

From Lemma 3.4 in [26], we see that, for $b \ge \max \{C_1(M,E_0,T,\varepsilon ), \mathfrak {B}(\varepsilon )\}$,

3.50 $$\begin{aligned} \bigg | \int ^t_0 u(s,b(s)) {\, \mathrm d}s \bigg | \le \frac{1}{4}b. \end{aligned}$$

Using (2.16), (3.49), and (3.50), we obtain

$$\begin{aligned} \begin{aligned}&\bigg | \int ^t_0 (\rho r^{n-1}(\Phi _\alpha * \rho )_r)(s,b(s)) {\, \mathrm d}s \bigg | \\&\quad \le \int ^t_0 \rho _0(b) \Big ( \int ^{b(s)}_a \Big | \int _{\mathbb {S}^{n-1}} \frac{b(s)\boldsymbol{e_1}-\eta \boldsymbol{y}}{|b(s)\boldsymbol{e_1}-\eta \boldsymbol{y}|^{\alpha + 2}} \cdot \boldsymbol{e_1} {\, \mathrm d}\sigma (\boldsymbol{y}) \Big | \rho (s,\eta ) \eta ^{n-1} {\, \mathrm d}\eta \Big ) b(s)^{n-1} {\, \mathrm d}s \\&\quad \le C \int ^t_0 \rho _0(b) \Big ( \int ^{b(s)}_a \rho (s,\eta ) \eta ^{n-1} {\, \mathrm d}\eta \Big ) b(s)^{n-1} {\, \mathrm d}s \\&\quad \le C(M) \int ^t_0 \rho _0(b) b(s)^{n-1} {\, \mathrm d}s \\&\quad = C(M) \int ^t_0 \rho _0(b) \Big ( b + \int ^s_0 u(\tau ,b(\tau )) {\, \mathrm d}\tau \Big )^{n-1} {\, \mathrm d}s \\&\quad \le C(M) \int ^t_0 \rho _0(b) b^{n-1} {\, \mathrm d}s \\&\quad \le C(M,T) b^{-(n-\beta )} b^{n-1} \\&\quad \le C(M,T), \end{aligned} \end{aligned}$$

where we have used that $\rho _0(b) \cong b^{-(n-\beta )}$, with $\beta = \min \{ \frac{1}{2},(1 - \frac{1}{\gamma })n \}$. We can also obtain this bound for α=n-2 from [26]. Hence, similar to [26], we see that, for κ=-1 with $\alpha \in (-1,n-2]$ or κ=1 with α=n-2,

$$\begin{aligned} \begin{aligned}&\frac{\varepsilon ^2}{4}\int _a^{b(t)} \big |(\sqrt{\rho (t,r)})_r\big |^2\,r^{n-1} {\, \mathrm d}r +\frac{4 a_0\varepsilon }{\gamma } \int _0^t\int _a^{b(s)} \big |(\rho ^{\frac{\gamma }{2}})_r\big |^2\,r^{n-1}{\, \mathrm d}r{\, \mathrm d}s\\&\quad \quad +\frac{1}{n}p(\rho (t,b(t)))b(t)^n +\frac{1}{n\varepsilon }\int _0^t p(\rho (s,b(s)))p'(\rho (s,b(s))) b(s)^n{\, \mathrm d}s \\&\quad \le C(E_0,M,T). \end{aligned} \end{aligned}$$

□

#### Remark 3.10

In Step 2 above, when κ=1, it follows from (3.47) that the boundary term given by $\kappa \varepsilon a^{n-1}(\rho (\Phi _\alpha * \rho )_r)(t,a)$ has a bad sign. When $\alpha \in (-1,n-2)$, we have

3.51 $$\begin{aligned} a^{n-1}(\Phi _\alpha * \rho )_r(t,a)&= \int ^a_0 (r^{n-1}(\Phi _\alpha * \rho )_r)_r {\, \mathrm d}r\nonumber \\&= \frac{(n-2) - \alpha }{\omega _n} \int _{B_a} (|\cdot |^{-(\alpha + 2)} * \rho )(\boldsymbol{y}) {\, \mathrm d}\boldsymbol{y}>0. \end{aligned}$$

For $\gamma < \frac{n}{(n-2) - \alpha }$, by the HLS inequality from Lemma A.2 and (3.51), we have

$$\begin{aligned} a^{n-1}(\Phi _\alpha * \rho )_r(t,a)&\le C \Vert \rho \Vert _{L^\gamma (\Omega _t)} \le C(E_0). \end{aligned}$$

For $\gamma \ge \frac{n}{(n-2) - \alpha }$, using the above and the interpolation, we obtain

$$\begin{aligned} \begin{aligned} a^{n-1}(\Phi _\alpha * \rho )_r(t,a)&\le C(M,E_0). \end{aligned} \end{aligned}$$

Thus, with such estimates, in order to control $\kappa \varepsilon a^{n-1}(\rho (\Phi _\alpha * \rho )_r)(t,a)$, it suffices to control ρ(t,a). However, such an *a priori* estimate on the density at r=a has not yet been available.

We now prove the second BD-type estimate result. Here we are able to provide a result for both the repulsive and attractive case $\kappa \in \{-1,1\}$ and for the more singular Riesz potential $\alpha \in (0,n-1)$. However, the estimate given by the Grönwall inequality is rough, compared to that of the case where integration by parts can be applied, so that we obtain a different range of γ for which it can be applied. This is due to the non-locality of the Riesz potential, while the potential is a local operator in the already proved Coulomb case α=n-2.

#### Lemma 3.11

(BD-Type Entropy Estimate II). Under the conditions of Lemma 3.4, for $\alpha \in (-1,n-1)$, $\gamma \ge \frac{3n}{3n - 2(1+\alpha )}$, any given T>0, $\varepsilon \in (0,\varepsilon _0]$, and $b \ge \max \{C_1(M,E_0,T,\varepsilon ), \mathfrak {B}(\varepsilon )\}$ (as defined in Lemmas 3.8 and B.8), the following uniform estimate holds for t∈[0,T]:

$$\begin{aligned} \begin{aligned}&\frac{\varepsilon ^2}{4}\int _a^{b(t)} \big |(\sqrt{\rho (t,r)})_r\big |^2\,r^{n-1} {\, \mathrm d}r +\frac{4 a_0\varepsilon }{\gamma } \int _0^t\int _a^{b(s)} \big |(\rho ^{\frac{\gamma }{2}})_r\big |^2\,r^{n-1}{\, \mathrm d}r{\, \mathrm d}s\\&\quad \quad +\frac{1}{n}p(\rho (t,b(t)))b(t)^n +\frac{1}{n\varepsilon }\int _0^t p(\rho (s,b(s)))p'(\rho (s,b(s))) b(s)^n{\, \mathrm d}s \\&\quad \le C(E_0,M,T). \end{aligned} \end{aligned}$$

#### Proof

We use calculations (3.40)–(3.45) done in Lemma 3.9. Substituting (3.45) into (3.43), we have

3.52 $$\begin{aligned}&\frac{\textrm{d}}{\textrm{d}\tau }\bigg \{\int _0^{\frac{M}{\omega _n}}\frac{1}{2}(u+\varepsilon r^{n-1}\rho _x)^2\,{\, \mathrm d}x +\int _0^{\frac{M}{\omega _n}}e(\rho )\,{\, \mathrm d}x \bigg \} +\varepsilon \int _0^{\frac{M}{\omega _n}}p'(\rho )\rho _x^2\,r^{2n-2} {\, \mathrm d}x\nonumber \\&\quad \quad +\Big (\frac{1}{n}p(\rho (\tau ,\frac{M}{\omega _n}))b(\tau )^n\Big )_\tau +\frac{1}{n\varepsilon }p(\rho (\tau ,\frac{M}{\omega _n}))p'(\rho (\tau ,\frac{M}{\omega _n})) b(\tau )^n \nonumber \\&\quad = - \kappa \int ^{\frac{M}{\omega _n}}_0 (u + \varepsilon r^{n-1} \rho _x)\rho r^{n-1}(\Phi _\alpha * \rho )_x {\, \mathrm d}x. \end{aligned}$$

In the case that $\gamma \ge \frac{3n}{3n - 2(1+\alpha )}$, we apply the Hölder inequality and then the HLS inequality from Lemma A.2 to obtain

3.53 $$\begin{aligned}&\int ^{b(t)}_a |(\Phi _\alpha * \rho )_r(t,r)|^2 \rho (t,r) r^{n-1} {\, \mathrm d}r\nonumber \\&\quad \le \frac{1}{\omega _n} \int _{\Omega _t} |\nabla (\Phi _\alpha * \rho )(t,\boldsymbol{y})|^2 \rho (t,\boldsymbol{y}) {\, \mathrm d}\boldsymbol{y} \nonumber \\&\quad \le C \Vert \nabla (\Phi _\alpha * \rho ) \Vert ^2_{L^{\frac{3n}{1+\alpha }}(\Omega _t)} \Vert \rho \Vert _{L^{\frac{3n}{3n - 2(1+\alpha )}}(\Omega _t)} \nonumber \\&\quad \le C \Vert |\cdot |^{-(\alpha + 1)}* \rho \Vert ^2_{L^{\frac{3n}{1+\alpha }}(\Omega _t)} \Vert \rho \Vert _{L^{\frac{3n}{3n - 2(1+\alpha )}}(\Omega _t)} \nonumber \\&\quad \le C \Vert \rho \Vert ^3_{L^{\frac{3n}{3n - 2(1+\alpha )}}(\Omega _t)}\nonumber \\&\quad \le C \Vert \rho \Vert ^{3\theta }_{L^\gamma (\Omega _t)} \Vert \rho \Vert ^{3(1 - \theta )}_{L^1(\Omega _t)} \nonumber \\&\quad \le C(M,E_0), \end{aligned}$$

where $\theta = \frac{2\gamma (1 + \alpha )}{3n(\gamma -1)}$. Applying the Cauchy-Schwarz inequality to (3.52) and using (3.53), we have

3.54 $$\begin{aligned} \begin{aligned}&\frac{\textrm{d}}{\textrm{d}\tau }\bigg \{\int _0^{\frac{M}{\omega _n}}\frac{1}{2}(u+\varepsilon r^{n-1}\rho _x)^2 {\, \mathrm d}x +\int _0^{\frac{M}{\omega _n}}e(\rho )\,{\, \mathrm d}x \bigg \} +\varepsilon \int _0^{\frac{M}{\omega _n}}p'(\rho )\rho _x^2\,r^{2n-2} {\, \mathrm d}x \\&\quad \quad +\Big (\frac{1}{n}p(\rho (\tau ,\frac{M}{\omega _n}))b(\tau )^n\Big )_\tau +\frac{1}{n\varepsilon }p(\rho (\tau ,\frac{M}{\omega _n}))p'(\rho (\tau ,\frac{M}{\omega _n})) b(\tau )^n \\&\quad \le C(M,E_0) + \int ^{\frac{M}{\omega _n}}_0 \frac{1}{2}\big (u + \varepsilon r^{n-1} \rho _x\big )^2 {\, \mathrm d}x.\\ \end{aligned} \end{aligned}$$

Applying the Grönwall type inequality to (3.54) yields

$$\begin{aligned} \begin{aligned}&\int _0^{\frac{M}{\omega _n}}\frac{1}{2}(u+\varepsilon r^{n-1}\rho _x)^2 {\, \mathrm d}x \\&\quad \quad + \int _0^\tau e^{\tau -s} \bigg \{ \Big (\int _0^{\frac{M}{\omega _n}}e(\rho )\,{\, \mathrm d}x \Big )_s + \varepsilon \int _0^{\frac{M}{\omega _n}}p'(\rho )\rho _x^2\,r^{2n-2} {\, \mathrm d}x +\Big (\frac{1}{n}p(\rho (s,\frac{M}{\omega _n}))b(s)^n\Big )_s \\&\qquad \qquad \qquad \qquad \quad + \frac{1}{n\varepsilon }p(\rho (s,\frac{M}{\omega _n}))p'(\rho (s,\frac{M}{\omega _n})) b(s)^n \bigg \} {\, \mathrm d}s \\&\quad \le C(M,E_0) \int ^\tau _0 e^{\tau -s} {\, \mathrm d}s + e^\tau \int _0^{\frac{M}{\omega _n}}\frac{1}{2}(u_0+\varepsilon r^{n-1}\rho _{0,x})^2 {\, \mathrm d}x. \end{aligned} \end{aligned}$$

Integrating by parts, we have

3.55 $$\begin{aligned}&\int _0^{\frac{M}{\omega _n}}\frac{1}{2}(u+\varepsilon r^{n-1}\rho _x)^2 {\, \mathrm d}x + \int _0^{\frac{M}{\omega _n}}e(\rho )\,{\, \mathrm d}x + \frac{1}{n}p(\rho (\tau ,\frac{M}{\omega _n}))b(\tau )^n\nonumber \\&\quad \quad + \int _0^\tau e^{\tau -s} \bigg \{ \int _0^{\frac{M}{\omega _n}}e(\rho )\,{\, \mathrm d}x + \varepsilon \int _0^{\frac{M}{\omega _n}}p'(\rho )\rho _x^2\,r^{2n-2} {\, \mathrm d}x + \frac{1}{n}p(\rho (s,\frac{M}{\omega _n}))b(s)^n\nonumber \\&\qquad \qquad \qquad \qquad \quad + \frac{1}{n\varepsilon }p(\rho (s,\frac{M}{\omega _n}))p'(\rho (s,\frac{M}{\omega _n})) b(s)^n \Bigg \} {\, \mathrm d}s \nonumber \\&\quad \le e^\tau \bigg \{ \int _0^{\frac{M}{\omega _n}}\frac{1}{2}(u_0+\varepsilon r^{n-1}\rho _{0,x})^2 {\, \mathrm d}x + \int _0^{\frac{M}{\omega _n}}e(\rho _0)\,{\, \mathrm d}x \bigg \}\nonumber \\&\qquad + C(M,E_0) (e^\tau - 1) + \frac{e^\tau }{n}p(\rho _0(\frac{M}{\omega _n}))b^n. \end{aligned}$$

Using the positivity of the integrands in the integral of the second line in (3.55), we conclude the following inequality:

$$\begin{aligned} \begin{aligned}&\int _0^{\frac{M}{\omega _n}}\frac{1}{2}(u+\varepsilon r^{n-1}\rho _x)^2 {\, \mathrm d}x + \int _0^{\frac{M}{\omega _n}}e(\rho )\,{\, \mathrm d}x + \frac{1}{n}p(\rho (\tau ,\frac{M}{\omega _n}))b(\tau )^n \\&\quad \quad + \int _0^\tau \bigg \{ \varepsilon \int _0^{\frac{M}{\omega _n}}p'(\rho )\rho _x^2\,r^{2n-2} {\, \mathrm d}x + \frac{1}{n\varepsilon }p(\rho (s,\frac{M}{\omega _n}))p'(\rho (s,\frac{M}{\omega _n})) b(s)^n \bigg \} {\, \mathrm d}s \\&\quad \le e^\tau \bigg \{ \int _0^{\frac{M}{\omega _n}}\frac{1}{2}(u_0+\varepsilon r^{n-1}\rho _{0,x})^2 {\, \mathrm d}x + \int _0^{\frac{M}{\omega _n}}e(\rho _0)\,{\, \mathrm d}x \bigg \} \nonumber \\&\qquad + C(M,E_0) (e^\tau - 1) + \frac{e^\tau }{n}p(\rho _0(\frac{M}{\omega _n}))b^n. \end{aligned} \end{aligned}$$

□

### Higher integrability

In order to apply the compensated compactness framework in Chen-Perepelitsa [27] to prove the convergence of weak solutions $(\rho ^\varepsilon ,\rho ^\varepsilon u^\varepsilon )$ of (1.6) to finite-energy solutions of (1.1)–(1.4) as $\varepsilon \rightarrow 0$, we need higher integrability uniform estimates of the density and the velocity. In the following lemma, we require that $\gamma > \frac{1}{n-1- \alpha }$. Indeed, this is only required so that we can obtain a pointwise estimate for the derivative of the convolution of the kernel with the density.

#### Lemma 3.12

(Higher Integrability of the Density). Let (ρ,u) be the smooth solution of (2.10). Then, under the assumptions of Theorem 2.5, T>0, $\varepsilon \in (0,\varepsilon _0]$, and $b \ge \max \{C_1(M,E_0,T,\varepsilon ), \mathfrak {B}(\varepsilon )\}$ (as defined in Lemmas 3.8 and B.8),

$$\begin{aligned} \int ^T_0 \int _K \rho ^{\gamma + 1}(t,r) {\, \mathrm d}r {\, \mathrm d}t \le C(K,M,E_0,T) \end{aligned}$$

for any K⋐(a,b(t)) and any t∈[0,T].

#### Proof

We follow a similar proof to that of [26]. Given K⋐(a,b(t)) for any t∈[0,T], there exists a<d<D such that $K \Subset (d,D) \Subset (a,b(t))$. Let $w \in C^\infty _0(\mathbb {R})$ such that $\textrm{supp}(w) \subset (d,D)$ and w(r)=1 for all r∈K. Next, we multiply $(2.10)_2$ by *w* to obtain the equation:

3.56 $$\begin{aligned}&(\rho u w)_t + \big ((\rho u^2 + p(\rho ))w\big )_r + \frac{n-1}{r}\rho u^2w + \kappa \rho (\Phi _\alpha * \rho )_rw \nonumber \\&\quad = \varepsilon \Big ( \rho \big (u_r + \frac{n-1}{r}u \big ) w \Big )_r - \varepsilon \frac{n-1}{r}\rho _ru w + \Big ( \rho u^2 + p(\rho ) - \varepsilon \rho \big (u_r + \frac{n-1}{r}u \big ) \Big ) \Big )w_r. \end{aligned}$$

We integrate (3.56) over [*d*, *r*) and then multiply by ρw to obtain

3.57 $$\begin{aligned} \rho p(\rho ) w^2&= \varepsilon \rho ^2\big (u_r+\frac{n-1}{r} u\big ) w^2 -\varepsilon \rho w\int _d^r \frac{n-1}{z} u\rho _z w{\, \mathrm d}z -\Big (\rho w\int _d^r\rho u w {\, \mathrm d}z\Big )_t\nonumber \\&\quad -\Big (\rho uw\int _d^r\rho u w {\, \mathrm d}z\Big )_r+\rho uw_r \int _d^r \rho u w {\, \mathrm d}z -\frac{n-1}{r} \rho u w \int _d^r \rho u w {\, \mathrm d}z\nonumber \\&\quad -\rho w\int _d^r \frac{n-1}{z}\rho u^2 w {\, \mathrm d}z +\rho w\int _d^r\Big (\rho u^2+p(\rho ) - \varepsilon \rho (u_z+\frac{n-1}{z}u)\Big )w_z {\, \mathrm d}z\nonumber \\&\quad - \kappa \rho w \int ^r_d \rho (\Phi _\alpha * \rho )_z w {\, \mathrm d}z =: \sum _{i=1}^9 I_i. \end{aligned}$$

As in [26], we have

3.58 $$\begin{aligned} \bigg | \int ^T_0 \int ^D_d \Big ( \sum _{i=1}^8 I_i \Big ) {\, \mathrm d}r {\, \mathrm d}t \bigg | \le C(d,M,E_0,T). \end{aligned}$$

When $\alpha \in (n-2,n-1)$, employing (3.1) and (3.4) with the Fubini theorem yields

3.59 $$\begin{aligned}&\left| \int ^T_0 \int ^D_d I_9 {\, \mathrm d}r {\, \mathrm d}t \right| \nonumber \\&\quad \le C \int ^T_0 \int ^D_d (\rho w)(t,r) \int ^r_d (\rho w)(t,z) \bigg ( \int _{a,b(t)) \cap (z - \frac{d}{2},z + \frac{d}{2})} \omega (z,\eta ) \rho (t,\eta ) \eta ^{n-1} {\, \mathrm d}\eta \nonumber \\&\qquad \qquad \qquad \qquad \qquad \qquad \qquad \qquad \qquad \qquad \qquad + \int _{(a,b(t)) \cap (z - \frac{d}{2},z + \frac{d}{2})^c} \omega (z,\eta ) \rho (t,\eta ) \eta ^{n-1} {\, \mathrm d}\eta \bigg ) {\, \mathrm d}z {\, \mathrm d}r {\, \mathrm d}t \nonumber \\&\quad \le C \int ^T_0 \int ^D_d (\rho w)(t,r) \int ^r_d (\rho w)(t,z) \bigg ( \int _{(a,b(t)) \cap (z - \frac{d}{2},z + \frac{d}{2})} \frac{|z-\eta |^{n-2-\alpha }}{({z}\eta )^{\frac{n-1}{2}}} \rho (t,\eta ) \eta ^{n-1} {\, \mathrm d}\eta \nonumber \\&\qquad \qquad \qquad \qquad \qquad \qquad \qquad \qquad \qquad \qquad \qquad + \int _{(a,b(t)) \cap (z - \frac{d}{2},z + \frac{d}{2})^c} |z-\eta |^{-(\alpha + 1)} \rho (t,\eta ) \eta ^{n-1}{\, \mathrm d}\eta \bigg ) {\, \mathrm d}z {\, \mathrm d}r {\, \mathrm d}t \nonumber \\&\quad \le C(d) \int ^T_0 \int ^D_d (\rho w)(t,r) \int ^r_d (\rho w)(t,z) \int _a^{b(t)} \big (1 + |z-\eta |^{n-2-\alpha }\big ) \rho (t,\eta ) \eta ^{n-1} {\, \mathrm d}\eta {\, \mathrm d}z {\, \mathrm d}r {\, \mathrm d}t. \nonumber \\&\quad \le C(d,M,T) + C(d,D) \int ^T_0 \int ^D_d (\rho w)(t,r) \Big (\int _a^{b(t)} {\rho (t,\eta ) \eta ^{n-1}} \int ^D_d |z-\eta |^{n-2-\alpha } (\rho w)(t,z) {\, \mathrm d}z {\, \mathrm d}\eta \Big ){\, \mathrm d}r {\, \mathrm d}t. \end{aligned}$$

For $\gamma > \frac{1}{n-1 - \alpha }$, by Theorem A.1, we obtain

3.60 $$\begin{aligned}&\bigg | \int ^T_0 \int ^D_d I_9 {\, \mathrm d}r {\, \mathrm d}t \bigg | \nonumber \\&\quad \le C(d,M,T) \nonumber \\&\quad \quad + C(d,D) \int ^T_0 \int ^D_d (\rho w)(t,r) \Big (\int _a^{b(t)} {\rho (t,\eta ) \eta ^{n-1}} \sup _{s \in \mathbb {R}_+} \Big \{ \int ^D_d {|z-s|}^{n-2-\alpha } (\rho w)(t,z) {\, \mathrm d}z \Big \}{\, \mathrm d}\eta \Big ) {\, \mathrm d}r {\, \mathrm d}t \nonumber \\&\quad = C(d,M,T) \nonumber \\&\quad \quad + C(d,D) \int ^T_0 \int ^D_d (\rho w)(t,r)\Big ( \int _a^{b(t)} {\rho (t,\eta ) \eta ^{n-1}} \sup _{s \in [d,D]} \Big \{ \int ^D_d {|z-s|}^{n-2-\alpha } (\rho w)(t,z) {\, \mathrm d}z \Big \} {\, \mathrm d}\eta \Big ) {\, \mathrm d}r {\, \mathrm d}t \nonumber \\&\quad \le C(d,M,T) + C(d,D) \Vert \rho \Vert _{L^\gamma (d,D)} \int ^T_0 \int ^D_d (\rho w)(t,r)\Big ( \int _a^{b(t)} {\rho (t,\eta ) \eta ^{n-1}} {\, \mathrm d}\eta \Big ) {\, \mathrm d}r {\, \mathrm d}t. \nonumber \\&\quad \le C(d,D,M,E_0,T). \end{aligned}$$

When $\alpha \in (-1,n-2)$, by a similar approach to (3.59) and using (3.1), we have

3.61 $$\begin{aligned}&\bigg | \int ^T_0 \int ^D_d I_9 {\, \mathrm d}r {\, \mathrm d}t \bigg | \nonumber \\&\quad \le C \int ^T_0 \int ^D_d (\rho w)(t,r) \int ^r_d (\rho w)(t,z) \bigg ( \int _{(a,b(t)) \cap (z - \frac{d}{2},z + \frac{d}{2})} \omega (z,\eta ) \rho (t,\eta ) \eta ^{n-1} {\, \mathrm d}\eta \nonumber \\&\qquad \qquad \qquad \qquad \qquad \qquad \qquad \qquad \qquad \qquad \qquad + \int _{(a,b(t)) \cap (z - \frac{d}{2},z + \frac{d}{2})^c} \omega (z,\eta ) \rho (t,\eta ) \eta ^{n-1} {\, \mathrm d}\eta \bigg ) {\, \mathrm d}z {\, \mathrm d}r {\, \mathrm d}t \nonumber \\&\quad \le C(d,M,T)\nonumber \\&\qquad + C \int ^T_0 \int ^D_d (\rho w)(t,r) \Big (\int ^r_d (\rho w)(t,z) \int _{(a,b(t)) \cap (z - \frac{d}{2},z + \frac{d}{2})} (r\eta )^{-\frac{\alpha + 1}{2}} \rho (t,\eta ) \eta ^{n-1} {\, \mathrm d}\eta {\, \mathrm d}z\Big ) {\, \mathrm d}r {\, \mathrm d}t \nonumber \\&\quad \le C(d,M,T) + C(d) \int ^T_0 \int ^D_d (\rho w)(t,r) \Big ( \int ^r_d (\rho w)(t,z) \int _a^{b(t)} \rho (t,\eta ) \eta ^{n-1} {\, \mathrm d}\eta {\, \mathrm d}z\Big ) {\, \mathrm d}r {\, \mathrm d}t \nonumber \\&\quad \le C(d,M,T). \end{aligned}$$

When α=n-2, using (3.1), we obtain

3.62 $$\begin{aligned} \bigg | \int ^T_0 \int ^D_d I_9 {\, \mathrm d}r {\, \mathrm d}t \bigg |&= \int ^T_0 \int ^D_d (\rho w)(t,r) \Big (\int ^r_d (\rho w)(t,z) \int _a^{r} \frac{\omega _n}{r^{n-1}} \rho (t,\eta ) \eta ^{n-1} {\, \mathrm d}\eta {\, \mathrm d}z\Big ) {\, \mathrm d}r {\, \mathrm d}t \nonumber \\&\quad \le C(d,M,T). \end{aligned}$$

Therefore, integrating (3.57) over [0,T]×[d,D] and using (3.58)–(3.62), we conclude the desired result. □

For the next result, we need to make use of entropy pairs. As defined in Lax [63], a pair of functions $(\eta (\rho ,m),q(\rho ,m))$ is called an entropy pair of the 1-D isentropic Euler system if they satisfy

$$\begin{aligned} \partial _t \eta (\rho (t,r),m(t,r)) + \partial _r q(\rho (t,r),m(t,r)) = 0 \end{aligned}$$

for any smooth solution (ρ,m) of the Euler system. Moreover, η is called a weak entropy if

$$\begin{aligned} \eta |_{\rho = 0}=0 \qquad \text { for all fixed}~u = \frac{m}{\rho }. \end{aligned}$$

From [74], it has been shown that any weak entropy pair (η,q) can be represented by

3.63 $$\begin{aligned} {\left\{ \begin{array}{ll} \displaystyle \eta ^\psi (\rho ,m) = \eta ^\psi (\rho ,\rho u) = \int _\mathbb {R} \chi (\rho ;s-u) \psi (s) {\, \mathrm d}s,\\ \displaystyle q^\psi (\rho ,m) = q^\psi (\rho , \rho u) = \int _\mathbb {R}(\theta s + (1 - \theta )u) \chi (\rho ;s-u) \psi (s) {\, \mathrm d}s, \end{array}\right. } \end{aligned}$$

where $\chi (\rho ;s-u) = (\rho ^{2\theta } - (s-u)^2)_+^{\mathfrak {b}}$ with $\mathfrak {b} = \frac{3 - \gamma }{2(\gamma -1)}$, $\theta = \frac{\gamma - 1}{2}$, and $(\cdot )_+ = \max \{\cdot , 0\}$. Choosing $\psi (s) = \frac{1}{2}s|s|$ as in [26], we obtain the corresponding entropy pair $(\eta ^{\#}(\rho , m), q^{\#}(\rho ,m))$ that is represented by

3.64 $$\begin{aligned} {\left\{ \begin{array}{ll} \displaystyle \eta ^{\#}(\rho ,m) = \eta ^{\#}(\rho ,\rho u) = \frac{1}{2} \rho \int _{-1}^1 (u+\rho ^{\theta } s) |u+\rho ^{\theta }s| (1-s^2)_+^{\mathfrak {b}} {\, \mathrm d}s,\\ \displaystyle q^{\#}(\rho ,m) = q^{\#}(\rho , \rho u) = \frac{1}{2} \rho \int _{-1}^1 (u+\theta \rho ^{\theta }s) (u+\rho ^{\theta } s)|u+\rho ^{\theta }s| (1-s^2)_+^{\mathfrak {b}} {\, \mathrm d}s. \end{array}\right. } \end{aligned}$$

Using (3.64), it can be shown that there exists $C_\gamma > 0$ such that the following inequalities hold:

3.65 $$\begin{aligned} \begin{aligned}&|\eta ^{\#}(\rho ,\rho u)| \le C_\gamma (\rho |u|^2 + \rho ^\gamma ), \quad q^\#(\rho , \rho u) \ge C_\gamma ^{-1}(\rho |u|^3 + \rho ^{\gamma + \theta }), \\&|\eta ^{\#}_m(\rho ,\rho u)| \le C_\gamma (|u| + \rho ^\theta ), \quad \quad |\eta ^{\#}_\rho (\rho ,\rho u)| \le C_\gamma (|u|^2 + \rho ^{2\theta }). \end{aligned} \end{aligned}$$

As in [27, 28], we have

3.66 $$\begin{aligned} \rho u \partial _\rho \eta ^{\#}+\rho u^2 \partial _m\eta ^{\#}-q^{\#}&=\frac{\theta }{2} \rho ^{1+\theta }\int _{-1}^1 (u-\rho ^{\theta }s)s |u+\rho ^{\theta }s|\, (1-s^2)_+^{\mathfrak {b}} {\, \mathrm d}s\le 0. \end{aligned}$$

#### Lemma 3.13

(Higher Integrability of the Velocity). Let (ρ,u) be the smooth solution of (2.10). Then, under the assumption of Lemma 3.12,

$$\begin{aligned} \int _0^T\int _d^D \big (\rho |u|^3 + \rho ^{\gamma +\theta }\big )(t,r)\,{\, \mathrm d}r {\, \mathrm d}t\le C(d,D,M,E_0,T) \end{aligned}$$

for any (d,D)⋐[a,b(t)] for any t∈[0,T].

#### Proof

Firstly, multiplying $(2.10)_1$ by $r^{n-1} \eta ^\#_\rho$ and $(2.10)_2$ by $r^{n-1} \eta ^\#_m$, we have

3.67 $$\begin{aligned}&(\eta ^\# r^{n-1})_t + (q^\# r^{n-1})_r + (n-1)\big (-q^\# + \rho u \eta ^\#_\rho + \rho u^2 \eta _m^\#\big ) r^{n-2}\nonumber \\&\quad = \varepsilon \eta ^\#_m \big ( (\rho u_r)_r + (n-1)\rho ( \frac{u}{r} )_r\big ) r^{n-1} - \kappa \eta ^\#_m \rho (\Phi _\alpha * \rho )_r r^{n-1}. \end{aligned}$$

Integrating (3.67) over [*r*, *b*(*t*)) and using (2.11) and (3.66), we obtain

3.68 $$\begin{aligned} q^{\#}(t,r)r^{n-1}&\le -\varepsilon \int _r^{b(t)} \eta ^{\#}_m(t,z)(\rho u_z)_z\, z^{n-1}{\, \mathrm d}z -(n-1)\varepsilon \int _r^{b(t)}\eta ^{\#}_m(t,z) \left( \frac{u}{z} \right) _z \rho \, z^{n-1}{\, \mathrm d}z\nonumber \\&\quad \, +\Big ( \int _r^{b(t)}\eta ^{\#}(t,z)\,z^{n-1}{\, \mathrm d}z\Big )_t +\big (q^{\#}-u\eta ^{\#}\big )(t,b(t))b(t)^{n-1}\nonumber \\&\quad \, +\kappa \int _r^{b(t)} \eta ^\#_m(t,z) \rho (\Phi _\alpha * \rho )_z z^{n-1} {\, \mathrm d}z =: \sum ^5_{i=1} I_i. \end{aligned}$$

Now, as in [26, Lemma 3.7], we have

$$\begin{aligned} \bigg | \int ^T_0 \int ^D_d \Big ( \sum ^4_{i = 1} I_i \Big ) {\, \mathrm d}r {\, \mathrm d}t \bigg | \le C(d,D,M,E_0,T). \end{aligned}$$

To estimate the potential term $I_5$, we use (3.65), the Young inequality, and once again the estimates in (3.1), and apply the same method as for (3.59) and (3.60)–(3.61) to obtain

3.69 $$\begin{aligned}&\bigg | \int ^T_0 \int ^D_d \int ^{b(t)}_r \kappa \eta ^{\#}_m \rho (\Phi _\alpha * \rho )_z z^{n-1} {\, \mathrm d}z {\, \mathrm d}r{\, \mathrm d}t\bigg |\nonumber \\&\quad \le C(d,D,M,E_0,T) \int ^T_0 \int ^D_d \int ^{b(t)}_r \big ((|u| + \rho ^\theta )\rho \big )(t,z) z^{n-1} {\, \mathrm d}z {\, \mathrm d}r {\, \mathrm d}t\nonumber \\&\quad \le C(d,D,M,E_0,T) \int ^T_0 \int ^D_d \int ^{b(t)}_r \big (\rho |u| + \rho ^\frac{\gamma }{2}\rho ^\frac{1}{2}\big )(t,z) z^{n-1} {\, \mathrm d}z {\, \mathrm d}r {\, \mathrm d}t \nonumber \\&\quad \le C(d,D,M,E_0,T) \int ^T_0 \int ^D_d \int ^{b(t)}_r\big (\rho u^2 + \rho + \rho ^\gamma \big )(t,z) z^{n-1} {\, \mathrm d}z {\, \mathrm d}r {\, \mathrm d}t\nonumber \\&\quad \le C(d,D,M,E_0,T). \end{aligned}$$

Combining $(3.64)_2$ with (3.68)–(3.69), we obtain the desired result. □

## Global Solutions to the Compressible Navier–Stokes-Riesz Equations

We now show the convergence of our solutions $(\rho ^{\varepsilon ,b},\rho ^{\varepsilon ,b}u^{\varepsilon ,b})$ of the free boundary problem (2.10)–(2.13) to global weak solutions $(\rho ^\varepsilon ,m^\varepsilon )$ of CNSREs (1.6) as $b \rightarrow \infty$. When we take the limit, the cavitation (vacuum states) may form, which should be taken into account carefully. For the following, we extend $(\rho ^{\varepsilon ,b},u^{\varepsilon ,b})$ to be zero on $([0,T] \times (0,\infty )) \setminus \Omega ^T$.

### Lemma 4.1

Under the assumptions of Theorem 2.5, for fixed $\varepsilon \in (0,\varepsilon _0]$ (as defined in Lemma B.8), there exists a non-negative function $\rho ^{\varepsilon }(t,r)$ a.e. such that, as $b\rightarrow \infty$ (up to a sub-sequence),4.1for any $q\in [1,\infty )$, where $L^q_\textrm{loc}$ denotes $L^q(K)$ for $K\Subset (0,\infty )$.

The proof is the same as for [26, Lemma 4.1].

### Corollary 4.2

Under the assumptions of Lemma 4.1, the pressure function sequence $p(\rho ^{\varepsilon ,b})$ is uniformly bounded in $L^\infty (0,T; L^q_\textrm{loc})$ for all $q\in [1,\infty ]$ and, as $b\rightarrow \infty$ (up to a sub-sequence),

$$\begin{aligned} p(\rho ^{\varepsilon ,b})\longrightarrow p(\rho ^{\varepsilon }) \qquad \text {strongly in } L^q(0,T; L^q_\textrm{loc}) \text { for all } q\in [1,\infty ). \end{aligned}$$

### Lemma 4.3

Under the assumptions of Lemma 4.1, as $b\rightarrow \infty$ (up to a sub-sequence), the momentum function sequence $m^{\varepsilon ,b}{:=}\rho ^{\varepsilon ,b} u^{\varepsilon ,b}$ converges strongly in $L^2(0,T; L^q_\textrm{loc})$ to some function $m^{\varepsilon }(t,r)$ for all $q\in [1,\infty )$, which particularly implies that

$$\begin{aligned} m^{\varepsilon ,b}=\rho ^{\varepsilon ,b} u^{\varepsilon ,b}\longrightarrow m^{\varepsilon }(t,r) \qquad \text {a.e. in } [0,T]\times (0,\infty ). \end{aligned}$$

### Proof

From Lemmas 3.4, 3.9, and 3.11, and the Sobolev embedding theorem, we see that $\sqrt{\rho ^{\varepsilon ,b}}$ is uniformly bounded in $L^\infty (0,T; H^{1}_\textrm{loc})\hookrightarrow L^\infty (0,T; L^{\infty }_\textrm{loc})$ for b>0, so that

$$ \rho ^{\varepsilon ,b}u^{\varepsilon ,b} = \sqrt{\rho ^{\varepsilon ,b}}\big (\sqrt{\rho ^{\varepsilon ,b}}u^{\varepsilon ,b} \big ) $$

is uniformly bounded in $L^\infty (0,T; L^2_\textrm{loc})$ for b>0. Since

$$\begin{aligned} \big (\rho ^{\varepsilon ,b}u^{\varepsilon ,b} \big )_r = \rho ^{\varepsilon ,b}_ru^{\varepsilon ,b} + \rho ^{\varepsilon ,b}u^{\varepsilon ,b}_r = 2 \big (\sqrt{\rho ^{\varepsilon ,b}}\big )_r \big ( \sqrt{\rho ^{\varepsilon ,b}}u^{\varepsilon ,b} \big ) + \sqrt{\rho ^{\varepsilon ,b}} \big ( \sqrt{\rho ^{\varepsilon ,b}}u^{\varepsilon ,b}_r \big ), \end{aligned}$$

it follows from Lemmas 3.4, 3.9, and 3.11 that $(\rho ^{\varepsilon ,b}u^{\varepsilon ,b})_r$ is uniformly bounded in $L^2(0,T; L^1_\textrm{loc})$ for b>0. This implies that

4.2 $$\begin{aligned} \rho ^{\varepsilon ,b}u^{\varepsilon ,b} \in L^2(0,T; W^{1,1}_\textrm{loc}) \qquad \text {uniformly for } b > 0. \end{aligned}$$

From Lemma 3.4 and the HLS inequality from Lemma A.2, we have

4.3 $$\begin{aligned} {\left\{ \begin{array}{ll} \displaystyle \partial _r\big ((\sqrt{\rho ^{\varepsilon ,b}} u^{\varepsilon ,b})^2\big )\in L^{\infty }(0,T;W_\textrm{loc}^{-1,1}),\\ \displaystyle \frac{n-1}{r} \big (\sqrt{\rho ^{\varepsilon ,b}} u^{\varepsilon ,b}\big )^2 \in L^{\infty }(0,T; L_\textrm{loc}^{1}),\\ \displaystyle \partial _r p(\rho ^{\varepsilon ,b}) \in L^{2}(0,T;H_\textrm{loc}^{-1}),\\ \displaystyle \rho ^{\varepsilon ,b} \big (\Phi _\alpha * \rho ^{\varepsilon ,b}\big )_r \in L^\infty (0,T; L^\infty _\textrm{loc}), \end{array}\right. } \end{aligned}$$

and

4.4 $$\begin{aligned} \sqrt{\rho ^{\varepsilon ,b}}\,\big (\sqrt{\rho ^{\varepsilon ,b}} u^{\varepsilon ,b}_r\big ) +\frac{n-1}{r} \sqrt{\rho ^{\varepsilon ,b}}\,\big (\sqrt{\rho ^{\varepsilon ,b}}u^{\varepsilon ,b}\big ) \in L^2(0,T; L^2_\textrm{loc}) \end{aligned}$$

uniformly for b>0. Hence, it follows from (4.4) that

4.5 $$\begin{aligned} \partial _r \big ( \rho ^{\varepsilon ,b} (u^{\varepsilon ,b}_r + \frac{n-1}{r}u^{\varepsilon ,b})\big ) \in L^2(0,T; H^{-1}_\textrm{loc}) \end{aligned}$$

uniformly for b>0. Using Lemmas 3.4, 3.9, and 3.11, we have

4.6 $$\begin{aligned} \frac{n-1}{r} u^{\varepsilon ,b} \partial _r \rho ^{\varepsilon ,b} =\frac{2(n-1)}{r} \big (\sqrt{\rho ^{\varepsilon ,b}}\big )_r\,\big (\sqrt{\rho ^{\varepsilon ,b}}u^{\varepsilon ,b}\big )\in L^2(0,T; L^1_\textrm{loc}) \end{aligned}$$

uniformly for b>0. Then, substituting (4.3) and (4.5)–(4.6) into $(2.10)_2$ and using the Sobolev embedding theorem, we see that

$$\begin{aligned} \partial _t(\rho ^{\varepsilon ,b} u^{\varepsilon ,b})\in L^{2}(0,T; W^{-1,q}_\textrm{loc}), \end{aligned}$$

uniformly for b>0 and $q \in [1,\frac{n}{n-1})$. Using the Aubin-Lions lemma together with (4.2), we obtain that $\rho ^{\varepsilon ,b} u^{\varepsilon ,b}$ is compact in $L^{2}(0,T;L^q_\textrm{loc})$ for all $q \in [1,\infty )$. □

### Lemma 4.4

Under the assumptions of Lemma 4.1, the limit function $m^\varepsilon (t,r)$ in Lemma 4.3 satisfies that $m^{\varepsilon }(t,r)=0$ a.e. on $\{(t,r):\,\rho ^{\varepsilon }(t,r)=0\}$. Furthermore, there exists a function $u^{\varepsilon }(t,r)$ such that $m^{\varepsilon }(t,r)=\rho ^{\varepsilon }(t,r) u^{\varepsilon }(t,r)$ a.e., and $\,u^\varepsilon (t,r)=0$ a.e. on $\{(t,r):\,\rho ^{\varepsilon }(t,r)=0\}$. Moreover, as $b\rightarrow \infty$ (up to a sub-sequence),

The proof of Lemma 4.4 is the same as [26, Lemma 4.4].

### Lemma 4.5

Under the assumptions of Lemma 4.1, let $0\le t_1<t_2\le T$, and let $\zeta (t,\boldsymbol{x})\in C^1_\textrm{c}([0,T]\times \mathbb {R}^n)$ be any smooth function with compact support. Then

4.7 $$\begin{aligned} \int _{\mathbb {R}^n} \rho ^{\varepsilon }(t_2,\boldsymbol{x}) \zeta (t_2,\boldsymbol{x})\, {\, \mathrm d}\boldsymbol{x} =\int _{\mathbb {R}^n} \rho ^{\varepsilon }(t_1,\boldsymbol{x}) \zeta (t_1,\boldsymbol{x})\, {\, \mathrm d}\boldsymbol{x} +\int _{t_1}^{t_2} \int _{\mathbb {R}^n} \big (\rho ^{\varepsilon } \zeta _t + {\mathcal {M}}^{\varepsilon }\cdot \nabla \zeta \big )\,{\, \mathrm d}\boldsymbol{x}{\, \mathrm d}t. \end{aligned}$$

Moreover, the total mass is conserved:

4.8 $$\begin{aligned} \int _{\mathbb {R}^n} \rho ^\varepsilon (t,\boldsymbol{x})\, {\, \mathrm d}\boldsymbol{x}=\int _{\mathbb {R}^n} \rho _0^\varepsilon (\boldsymbol{x})\, {\, \mathrm d}\boldsymbol{x}=M\qquad \, \text { for } t\ge 0. \end{aligned}$$

The proof of Lemma 4.5 is the same as [26, Lemma 4.9].

We can now prove an *a priori* estimate of the energy terms.

### Lemma 4.6

Under the assumptions of Lemma 4.1, the following estimates hold:

(i) When $$\alpha \in (0,n - 1)$$,
  $$\begin{aligned} \begin{aligned}&\int _0^\infty \Big (\frac{1}{2} \Big |\frac{m^{\varepsilon }}{\sqrt{\rho ^\varepsilon }} \Big |^2 + \rho ^{\varepsilon }e(\rho ^\varepsilon ) \Big )(t,r)\,r^{n-1}{\, \mathrm d}r + \varepsilon (n-1) \int ^t_0\int ^\infty _0 \Big |\frac{m^{\varepsilon }}{\sqrt{\rho ^\varepsilon }}\Big |^2 r^{n-3} {\, \mathrm d}r{\, \mathrm d}s \\&\quad \le C(M,E_0). \end{aligned} \end{aligned}$$
(ii) When $$\alpha \in (-1,0]$$,
  $$\begin{aligned} \begin{aligned}&\int _0^\infty \Big (\frac{1}{2} \Big |\frac{m^{\varepsilon }}{\sqrt{\rho ^\varepsilon }} \Big |^2 + \rho ^{\varepsilon }\big ( e(\rho ^\varepsilon ) + k_\alpha (1 + r^2)\big ) \Big )(t,r)\,r^{n-1}{\, \mathrm d}r\\&\qquad + \varepsilon (n-1) \int ^t_0\int ^\infty _0 \Big |\frac{m^{\varepsilon }}{\sqrt{\rho ^\varepsilon }} \Big |^2 r^{n-3} {\, \mathrm d}r{\, \mathrm d}s \\&\quad \le C(M,E_0,T). \end{aligned} \end{aligned}$$

### Proof

It follows directly from Lemma 3.4 that, for $\alpha \in (0,n - 1)$,

$$\begin{aligned} \begin{aligned}&\int _a^{b(t)} \Big (\frac{1}{2} \Big |\frac{m^{\varepsilon ,b}}{\sqrt{\rho ^{\varepsilon ,b}}} \Big |^2 + \rho ^{\varepsilon ,b}e(\rho ^{\varepsilon ,b}) \Big )(t,r)\,r^{n-1}{\, \mathrm d}r + \varepsilon (n-1) \int ^t_0\int _a^{b(s)} \Big |\frac{m^{\varepsilon ,b}}{\sqrt{\rho ^{\varepsilon ,b}}} \Big |^2 r^{n-3} {\, \mathrm d}r{\, \mathrm d}s \\&\quad \le C(n,M,E_0,T). \end{aligned} \end{aligned}$$

Now, applying the Fatou lemma, and Lemmas 3.8, 4.1, and 4.4, we obtain the result when $\alpha \in (0,n - 1)$. The approach is the same when $\alpha \in (-1,0]$. □

Notice that our estimates do not involve the potential term; this is because the convergence of the potential has not yet been proved. To do this, we require a few more steps. Since the mass is conserved in the limit, and the local convergence of the density has been verified, there is no blow-up of mass at the origin or a loss of mass at infinity; this allows us to prove some global convergence results.

### Lemma 4.7

Under the assumptions of Lemma 4.1, as $b\rightarrow \infty$ (up to a sub-sequence),That is, the convergence of $\rho ^{\varepsilon ,b}$ is global sub-sequentially in $L^q$.

### Proof

For all $\alpha \in (-1,n-1)$, it follows from Lemma 4.1 and (4.8) that

4.9 $$\begin{aligned} \lim _{k \rightarrow \infty } \inf _{t \in [0,T]} \int _\frac{1}{k}^k \rho ^\varepsilon (t,r) r^{n-1} {\, \mathrm d}r = \frac{M}{\omega _n}. \end{aligned}$$

Indeed, if not, then there exists ${\delta } > 0$ and $t_k \in [0,T]$ such that

4.10 $$\begin{aligned} \int _\frac{1}{k}^k \rho ^\varepsilon (t_k,r) r^{n-1} {\, \mathrm d}r < \frac{M}{\omega _n} - {\delta } \qquad \text {for all } k \in \mathbb {N}. \end{aligned}$$

Then there exists a sub-sequence of $(t_k)_k$ given by $(t_{k_l})_l$ and $t_* \in [0,T]$ such that $t_{k_l} \rightarrow t_*$ as $l \rightarrow \infty$. From (4.8), we see that, by the monotone convergence theorem, there exists $L \in \mathbb {N}$ such that

$$\begin{aligned} \int _\frac{1}{L}^L \rho ^\varepsilon (t_*,r) r^{n-1} {\, \mathrm d}r > \frac{M}{\omega _n} - {\delta }. \end{aligned}$$

Note, for $k_l \ge L$, using (4.10), we have

4.11 $$\begin{aligned} \int _\frac{1}{L}^L \rho ^\varepsilon (t_{k_l},r) r^{n-1} {\, \mathrm d}r \le \int _\frac{1}{k_l}^{k_l} \rho ^\varepsilon (t_{k_l},r) r^{n-1} {\, \mathrm d}r < \frac{M}{\omega _n} - {\delta }. \end{aligned}$$

By Lemma 4.1, $\rho ^\varepsilon \in C(0,T; L^1_\textrm{loc})$ so that

4.12 $$\begin{aligned} \int _\frac{1}{L}^L \rho ^\varepsilon (t_{k_l},r) r^{n-1} {\, \mathrm d}r \longrightarrow \int _\frac{1}{L}^L \rho ^\varepsilon (t_*,r) r^{n-1} {\, \mathrm d}r \qquad \text {as } l\rightarrow \infty . \end{aligned}$$

Thus, combining (4.11) with (4.12), we obtain

$$\begin{aligned} \int _\frac{1}{L}^L \rho ^\varepsilon (t_*,r) r^{n-1} {\, \mathrm d}r \le \frac{M}{\omega _n} - {\delta }, \end{aligned}$$

which is a contradiction. Thus, (4.9) is verified.

With this, there exists $k = k(\delta ) \in \mathbb {N}$ such that

4.13 $$\begin{aligned} \inf _{t \in [0,T]} \int _\frac{1}{k}^k \rho ^\varepsilon (t,r) r^{n-1} {\, \mathrm d}r > \frac{M}{\omega _n} - \frac{\delta }{4}, \end{aligned}$$

which, by (4.8) and (4.13), implies

4.14 $$\begin{aligned} \sup _{t \in [0,T]} \Big (\int _{k}^\infty + \int _0^\frac{1}{k} \Big ) \rho ^\varepsilon (t,r) r^{n-1} {\, \mathrm d}r < \frac{\delta }{4}. \end{aligned}$$

By (4.1), there exists B(δ)>0 such that, for $b \ge B(\delta )$ (up to a sub-sequence),

4.15 $$\begin{aligned} \begin{aligned} \sup _{t \in [0,T]} \int _\frac{1}{k}^k |\rho ^\varepsilon - \rho ^{\varepsilon ,b}|(t,r) r^{n-1} {\, \mathrm d}r < \frac{\delta }{4}. \end{aligned} \end{aligned}$$

Then it follows from (4.13)–(4.15) that, for $b \ge B(\delta )$,

4.16 $$\begin{aligned} \begin{aligned}&\inf _{t \in [0,T]} \int _\frac{1}{k}^k \rho ^{\varepsilon ,b}(t,r) r^{n-1} {\, \mathrm d}r > \frac{M}{\omega _n} - \frac{\delta }{2}, \\&\sup _{t \in [0,T]} \Big (\int _{k}^\infty + \int _0^\frac{1}{k} \Big ) \rho ^{\varepsilon ,b}(t,r) r^{n-1} {\, \mathrm d}r < \frac{\delta }{2}. \end{aligned} \end{aligned}$$

Thus, we obtain that, for b>B(δ),

$$\begin{aligned} \sup _{t \in [0,T]} \int _0^\infty |\rho ^\varepsilon - \rho ^{\varepsilon ,b}|(t,r) r^{n-1} {\, \mathrm d}r < \delta . \end{aligned}$$

Now, for $q \in [1,\gamma )$, using Lemmas 3.4 and 4.6, and performing interpolation, we have

$$\begin{aligned} \begin{aligned}&\Vert \rho ^\varepsilon - \rho ^{\varepsilon ,b} \Vert _{C(0,T;L^q(\mathbb {R}^n))} \\&\quad \le \Vert \rho ^\varepsilon - \rho ^{\varepsilon ,b} \Vert ^\frac{\gamma - q}{q(\gamma - 1)}_{C(0,T;L^1(\mathbb {R}^n))} \Vert \rho ^\varepsilon - \rho ^{\varepsilon ,b} \Vert ^\frac{\gamma (q - 1)}{q(\gamma - 1)}_{L^\infty (0,T;L^\gamma (\mathbb {R}^n))} \\&\quad \le C(n,M,E_0,T,q) \Vert \rho ^\varepsilon - \rho ^{\varepsilon ,b} \Vert ^\frac{\gamma - q}{q(\gamma - 1)}_{C(0,T;L^1(\mathbb {R}^n))} \longrightarrow 0, \end{aligned} \end{aligned}$$

as $b \rightarrow \infty$ (up to a sub-sequence). □

### Lemma 4.8

Under the assumptions of Lemma 4.1, the following statements hold as $b\rightarrow \infty$ (up to a sub-sequence):

4.17 $$\begin{aligned}&\Phi _\alpha *\rho ^{\varepsilon ,b} \mathrm - \!\!\! \rightharpoonup \Phi _\alpha *\rho ^\varepsilon \nonumber \\&\qquad \text{ weak- } * \text{ in } L^\infty (0,T; W^{1,\frac{2n}{\alpha +2}}_\textrm{loc}(\mathbb {R}^n)) \text{ and } \text{ weakly } \text{ in } L^2(0,T; W^{1,\frac{2n}{\alpha +2}}_\textrm{loc}(\mathbb {R}^n)) , \end{aligned}$$

4.18 $$\begin{aligned}&(\Phi _\alpha *\rho ^{\varepsilon ,b})_r(t,r)r^{n-1} \longrightarrow (\Phi _\alpha *\rho ^\varepsilon )_r(t,r)r^{n-1} \quad \text { in } C_\textrm{loc}([0,T]\times [0,\infty )), \end{aligned}$$

4.19 $$\begin{aligned}&(\Phi _\alpha *\rho ^{\varepsilon ,b})(t,r)r^{n-1} \longrightarrow (\Phi _\alpha *\rho ^\varepsilon )(t,r)r^{n-1} \qquad \text { in } C_\textrm{loc}([0,T]\times [0,\infty )), \end{aligned}$$

4.20 $$\begin{aligned}&\int _0^\infty \big |\rho ^{\varepsilon ,b} (\Phi _\alpha *\rho ^{\varepsilon ,b}) - \rho ^{\varepsilon } (\Phi _\alpha *\rho ^{\varepsilon })\big |(t,r)\, r^{n-1} {\, \mathrm d}r \longrightarrow 0. \end{aligned}$$

Moreover, when $$\alpha \in (0,n-1)$$,

4.21 $$\begin{aligned} \Vert (\Phi _\alpha *\rho ^\varepsilon )(t)\Vert _{L^{\frac{2n}{\alpha }}(\mathbb {R}^n)} +\Vert (\nabla \Phi _\alpha *\rho ^\varepsilon )(t)\Vert _{L^\frac{2n}{\alpha + 2}(\mathbb {R}^n)}\le C(M,E_0)\qquad \,\text {for } t\ge 0; \end{aligned}$$

when $\alpha \in (-1,0]$, for any $K \Subset \mathbb {R}^n$,

4.22 $$\begin{aligned} \Vert (\Phi _\alpha *\rho ^\varepsilon )(t)\Vert _{L^\infty (K)} +\Vert (\nabla \Phi _\alpha *\rho ^\varepsilon )(t)\Vert _{L^\frac{2n}{\alpha + 2}(K)} \le C(M,E_0,T,K)\qquad \,\text {for } t\ge 0. \end{aligned}$$

### Proof

We divide the proof into five steps.

1. We first see that (4.17) and (4.21)–(4.22) follow from Corollary 3.6 and the weak-*/weak compactness directly.

2. To show that the weak limit of $\Phi _\alpha * \rho ^{\varepsilon ,b}$, denoted by $\Phi _\alpha ^\varepsilon$, is indeed $\Phi _\alpha * \rho ^\varepsilon$, we test by a smooth compactly supported function $\varphi \in C^\infty _\textrm{c}(\mathbb {R}^n)$ so that it can be shown by shifting the convolution from $\rho ^{\varepsilon ,b}$ to φ that, as $b \rightarrow \infty$,

$$ \int _{\mathbb {R}^n} \varphi (\Phi _\alpha * \rho ^{\varepsilon ,b}) {\, \mathrm d}\boldsymbol{x} = \int _{\mathbb {R}^n} \rho ^{\varepsilon ,b} (\Phi _\alpha * \varphi ) {\, \mathrm d}\boldsymbol{x} \longrightarrow \int _{\mathbb {R}^n} \rho ^{\varepsilon } (\Phi _\alpha * \varphi ) {\, \mathrm d}\boldsymbol{x} = \int _{\mathbb {R}^n} \varphi (\Phi _\alpha * \rho ^{\varepsilon }) {\, \mathrm d}\boldsymbol{x}. $$

Using (4.17), we have

$$ \int _{\mathbb {R}^n} \varphi (\Phi _\alpha * \rho ^{\varepsilon ,b}) {\, \mathrm d}\boldsymbol{x} \longrightarrow \int _{\mathbb {R}^n} \varphi \,\Phi _\alpha ^\varepsilon {\, \mathrm d}\boldsymbol{x}. $$

Then, by the fundamental theorem of calculus of variations, $\Phi _\alpha ^\varepsilon = \Phi _\alpha * \rho ^{\varepsilon }$ *a.e.*. The result follows.

3. We now prove (4.18). When $\alpha \in (-1,n-1)$, for D>0, $(t,r) \in [0,T] \times [0,D]$, *b* sufficiently large, taking $0< \sigma< {\hat{\sigma }}<\infty$, we have

4.23 $$\begin{aligned}&\big |(\Phi _\alpha *\rho ^{\varepsilon ,b})_r r^{n-1} - (\Phi _\alpha *\rho ^\varepsilon )_r r^{n-1}\big |(t,r) \nonumber \\&\quad = r^{n-1} \Big | \int ^\infty _0 \omega (r,\eta ) (\rho ^{\varepsilon ,b} - \rho ^\varepsilon )(t,\eta ) \eta ^{n-1} {\, \mathrm d}\eta \Big | \nonumber \\&\quad \le r^{n-1} \Big | \int ^\sigma _0 \omega (r,\eta ) (\rho ^{\varepsilon ,b} - \rho ^\varepsilon )(t,\eta ) \eta ^{n-1} {\, \mathrm d}\eta \Big | \nonumber \\&\qquad + r^{n-1} \Big | \int ^{\hat{\sigma }}_\sigma \omega (r,\eta ) (\rho ^{\varepsilon ,b} - \rho ^\varepsilon )(t,\eta ) \eta ^{n-1} {\, \mathrm d}\eta \Big | \nonumber \\&\quad \quad + r^{n-1} \Big | \int ^\infty _{\hat{\sigma }} \omega (r,\eta ) (\rho ^{\varepsilon ,b} - \rho ^\varepsilon )(t,\eta ) \eta ^{n-1} {\, \mathrm d}\eta \Big |\nonumber \\&\quad =: I_1 + I_2 + I_3. \end{aligned}$$

For $I_1$, applying the Hölder inequality, we have

4.24 $$\begin{aligned}&I_1 \le r^{n-1} \Big (\int ^\sigma _0 \big ( (\rho ^{\varepsilon ,b})^\gamma + (\rho ^b)^\gamma \big )\eta ^{n-1} {\, \mathrm d}\eta \Big ) \Big | \int ^\sigma _0 \omega ^{\frac{\gamma }{\gamma - 1}}(r,\eta ) \eta ^{n-1} {\, \mathrm d}\eta \Big |^{1 - \frac{1}{\gamma }} \nonumber \\&\quad \le C(M,E_0) r^{n-1} \Big | \int ^\sigma _0 \omega ^{\frac{\gamma }{\gamma - 1}}(r,\eta ) \eta ^{n-1} {\, \mathrm d}\eta \Big |^{1 - \frac{1}{\gamma }}. \end{aligned}$$

When $\alpha \in (n-2,n-1)$, using (3.1), we have

4.25 $$\begin{aligned}&r^{n-1} \bigg | \int _0^\sigma \omega ^{\frac{\gamma }{\gamma - 1}}(r,\eta ) \eta ^{n-1} {\, \mathrm d}\eta \bigg |^{1 - \frac{1}{\gamma }} \nonumber \\&\quad \le C r^{n-1} \sigma ^{(n-1)(1 - \frac{1}{\gamma })} \bigg ( \int _{(0,\sigma )\cap (\frac{r}{2},\infty )}\omega ^{\frac{\gamma }{\gamma - 1}}(r,\eta ) {\, \mathrm d}\eta + \int _{(0,\sigma )\cap (0,\frac{r}{2})}\omega ^{\frac{\gamma }{\gamma - 1}}(r,\eta ) {\, \mathrm d}\eta \bigg )^{1 - \frac{1}{\gamma }} \nonumber \\&\quad \le C r^{n-1} \sigma ^{(n-1)(1 - \frac{1}{\gamma })} \bigg ( \int _{(0,\sigma )\cap (\frac{r}{2},\infty )} \Big ( \frac{|r-\eta |^{n-2 - \alpha }}{(r\eta )^{\frac{n-1}{2}}} \Big )^{\frac{\gamma }{\gamma - 1}} {\, \mathrm d}\eta + \int _{(0,\sigma )\cap (0,\frac{r}{2})} |r-\eta |^{-(\alpha +1)\frac{\gamma }{\gamma - 1}} {\, \mathrm d}\eta \bigg )^{1 - \frac{1}{\gamma }}\nonumber \\&\quad \le C r^{n-1} \sigma ^{(n-1)(1 - \frac{1}{\gamma })} \bigg ( r^{-(n-1)\frac{\gamma }{\gamma - 1}} \int _{(0,\sigma )\cap (\frac{r}{2},\infty )}|r-\eta |^{(n-2- \alpha )\frac{\gamma }{\gamma - 1}} {\, \mathrm d}\eta + r^{1 -(\alpha +1)\frac{\gamma }{\gamma - 1}} \bigg )^{1 - \frac{1}{\gamma }}\nonumber \\&\quad \le C \sigma ^{(n - 1)(1 - \frac{1}{\gamma })} + C r^{n - 1 - \alpha - \frac{1}{\gamma }} \sigma ^{(n-1)(1 - \frac{1}{\gamma })} \nonumber \\&\quad \le C(D) \sigma ^{(n-1)(1 - \frac{1}{\gamma })}, \end{aligned}$$

where the last inequality follows from the fact that $n - 1 - \alpha - \frac{1}{\gamma } > 0$, and the second to the last inequality follows from the fact that there exists C>0 such that, for small σ>0,

$$\begin{aligned} \int _{(0,\sigma )\cap (\frac{r}{2},\infty )}|r-\eta |^{(n-2 - \alpha )\frac{\gamma }{\gamma - 1}} {\, \mathrm d}\eta \le C\qquad \,\text {uniformly in } r, \end{aligned}$$

due to the fact that $(n-2 - \alpha )\frac{\gamma }{\gamma - 1} > -1$ as a consequence of the condition $\gamma > \frac{1}{n-1-\alpha }$.

When $\alpha \in (-1,n-2]$, applying (3.1) and taking the same approach to (4.25), we obtain that, for σ small enough,

4.26 $$\begin{aligned} \begin{aligned} r^{n-1} \left| \int _0^\sigma \omega ^{\frac{\gamma }{\gamma - 1}}(r,\eta ) \eta ^{n-1} {\, \mathrm d}\eta \right| ^{1 - \frac{1}{\gamma }} \le C(D) \sigma ^{n(1 - \frac{1}{\gamma })} \le C(D) \sigma ^{(n - 1)(1 - \frac{1}{\gamma })}. \end{aligned} \end{aligned}$$

Thus, combining (4.24)–(4.26) together, we have

4.27 $$\begin{aligned} \begin{aligned} \sup _{[0,T] \times [0,D]} I_1 \le C(D,M,E_0) \sigma ^{(n - 1)(1 - \frac{1}{\gamma })}\big (1 + \sigma ^{1 - \frac{1}{\gamma }}\big ) \rightarrow 0 \qquad \text { as}\quad \sigma \rightarrow 0. \end{aligned} \end{aligned}$$

Considering $I_2$, for $\alpha \in (-1,n-2]$, we use (3.1) to obtain

4.28 $$\begin{aligned}&I_2 \le C r^{n-1} \int ^{\hat{\sigma }}_\sigma (r\eta )^{-\frac{\alpha + 1}{2}} |\rho ^{\varepsilon ,b} - \rho ^\varepsilon |(t,\eta ) \eta ^{n-1} {\, \mathrm d}\eta \nonumber \\&\quad \le C({\hat{\sigma }}) r^{n - 2 - \alpha } \sigma ^{-\frac{\alpha + 1}{2}} \int ^{\hat{\sigma }}_\sigma |\rho ^{\varepsilon ,b} - \rho ^\varepsilon |(t,\eta ) {\, \mathrm d}\eta \nonumber \\&\quad \le C(D,{\hat{\sigma }},\sigma ) \Vert \rho ^{\varepsilon ,b} - \rho ^\varepsilon \Vert _{L^1(\sigma ,{\hat{\sigma }})}. \end{aligned}$$

For $\alpha \in (n-2,n-1)$, using the HLS inequality from Lemma A.2 and (3.4) yields

4.29 $$\begin{aligned}&I_2 \le C r^{n-1} \int ^{\hat{\sigma }}_\sigma \frac{|r - \eta |^{n-2-\alpha }}{(r\eta )^{\frac{n-1}{2}}}|\rho ^{\varepsilon ,b}-\rho ^\varepsilon |(t,\eta )\eta ^{n-1}{\, \mathrm d}\eta \nonumber \\&\quad \le C({\hat{\sigma }}) \Big ( \frac{r}{\sigma } \Big )^{\frac{n-1}{2}} \big \Vert |\cdot |^{n-2-\alpha } * ((\rho ^{\varepsilon ,b} - \rho ^\varepsilon )1\!\!1_{(\sigma ,{\hat{\sigma }})})\big \Vert _{C[0,D]} \nonumber \\&\quad \le C(D,{\hat{\sigma }},\sigma ) \Vert \rho ^{\varepsilon ,b} - \rho ^\varepsilon \Vert _{L^\gamma (\sigma ,{\hat{\sigma }})}. \end{aligned}$$

Combining (4.28) with (4.29), we obtain via Lemma 4.1 that

4.30 $$\begin{aligned} \sup _{[0,T] \times [0,D]} I_2 \le C(D,{\hat{\sigma }},\sigma )\sup _{[0,T]} \big (\Vert \rho ^{\varepsilon ,b} - \rho ^\varepsilon \Vert _{L^1(\sigma ,{\hat{\sigma }})} + \Vert \rho ^{\varepsilon ,b} - \rho ^\varepsilon \Vert _{L^\gamma (\sigma ,{\hat{\sigma }})}\big ) \rightarrow 0 \quad \text {as}\quad b \rightarrow \infty . \end{aligned}$$

Considering $I_3$, since $\omega (r,\eta ) \le C |r-\eta |^{-(\alpha + 1)}$, we have

4.31 $$\begin{aligned} \sup _{[0,T] \times [0,D]} I_3&\le C |D - {\hat{\sigma }}|^{-(\alpha + 1)} \sup _{[0,T]} \int ^\infty _{\hat{\sigma }} |\rho ^{\varepsilon ,b} - \rho ^\varepsilon |(t,\eta ) \eta ^{n-1} {\, \mathrm d}\eta \nonumber \\&\le C(M) |D - {\hat{\sigma }}|^{-(\alpha + 1)} \rightarrow 0 \qquad \text { as}\quad \hat{\sigma } \rightarrow \infty . \end{aligned}$$

Thus, combining (4.23) and (4.27) with (4.30)–(4.31), we conclude

$$\begin{aligned} \sup _{[0,T] \times [0,D]} |(\Phi _\alpha *\rho ^{\varepsilon ,b})_r r^{n-1} - (\Phi _\alpha *\rho ^\varepsilon )_r r^{n-1}|(t,r) \rightarrow 0 \qquad \text { as}\quad b \rightarrow \infty . \end{aligned}$$

Thus, (4.18) is proved.

4. Regarding (4.19), when $\alpha \in (0,n - 1)$, using the same method as for the calculations for ω in Lemma 3.1 with (3.1) and (3.3)–(3.4), we have

4.32 $$\begin{aligned} K(r,\eta ) \le C \min \big \{|r - \eta |^{-\alpha },\,(r \eta )^{- \frac{\alpha }{2}} \big \}. \end{aligned}$$

That is, K(r,η) has the same bound as ω from (3.1) but with α+1 replaced by α. Then the proof of (4.19) is verbatim the proof of (4.18) corresponding to the case that $\alpha \in (0,n-2)$.

When $\alpha \in (-1,0]$, taking ${\hat{\sigma }} >1$, we define $J_{\hat{\sigma }}(\boldsymbol{x}) {:=}\frac{1}{{\hat{\sigma }}^n} J(\frac{\boldsymbol{x}}{\hat{\sigma }})$, where *J* is a standard spherically symmetric mollifier kernel with $J \in C^\infty _\textrm{c}(\mathbb {R}^n)$, $\textrm{supp}(J) \subset B_1$, and $\int _{\mathbb {R}^n} J(\boldsymbol{x}) {\, \mathrm d}\boldsymbol{x} = 1$. Using (2.10) and integrating by parts, we have

$$\begin{aligned} \begin{aligned}&\frac{\textrm{d}}{\textrm{d}t} \int ^{b(t)}_a \rho ^{\varepsilon ,b} (1\!\!1_{B_{2{\hat{\sigma }}}^c} * J_{\hat{\sigma }}) k_\alpha (1 + r^2) r^{n-1} {\, \mathrm d}r \\&= \int ^{b(t)}_a \rho ^{\varepsilon ,b} u^{\varepsilon ,b} \left( (1\!\!1_{B_{2{\hat{\sigma }}}^c} * J_{\hat{\sigma }}) (k_\alpha (1 + r^2))_r + (1\!\!1_{B_{2{\hat{\sigma }}}^c} * J_{\hat{\sigma }})_r k_\alpha (1+ r^2) \right) r^{n-1} {\, \mathrm d}r. \end{aligned} \end{aligned}$$

Integrating over time, we obtain

4.33 $$\begin{aligned} \begin{aligned}&\int ^{b(t)}_a \rho ^{\varepsilon ,b} (1\!\!1_{B_{2{\hat{\sigma }}}^c} * J_{\hat{\sigma }}) k_\alpha (1 + r^2) r^{n-1} {\, \mathrm d}r\\&\quad = \int ^{b}_a \rho ^{\varepsilon ,b}_0 (1\!\!1_{B_{2{\hat{\sigma }}}^c} * J_{\hat{\sigma }}) k_\alpha (1 + r^2) r^{n-1} {\, \mathrm d}r\\&\qquad + \int ^t_0 \int ^{b(s)}_a \left( (1\!\!1_{B_{2{\hat{\sigma }}}^c} * J_{\hat{\sigma }}) (k_\alpha (1 + r^2))_r + (1\!\!1_{B_{2{\hat{\sigma }}}^c} * J_{\hat{\sigma }})_r k_\alpha (1+ r^2) \right) \rho ^{\varepsilon ,b}u^{\varepsilon ,b} r^{n-1} {\, \mathrm d}r {\, \mathrm d}s. \end{aligned} \end{aligned}$$

As in (3.37), for $\boldsymbol{x} \in K = \overline{B_D}$ and $q \in (1,\gamma )$, we use (3.36) and (3.38) to obtain

$$\begin{aligned} \begin{aligned}&\big |(\Phi _\alpha * \rho ^{\varepsilon ,b})(\boldsymbol{x}) - (\Phi _\alpha * \rho ^{\varepsilon })(\boldsymbol{x})\big |\\&\quad \le \int _{B_{3{\hat{\sigma }}}(\boldsymbol{x})} k_\alpha (|\boldsymbol{x} - \boldsymbol{y}|) |\rho ^{\varepsilon ,b} - \rho ^{\varepsilon }|(\boldsymbol{y}) {\, \mathrm d}\boldsymbol{y} + \int _{B_{3{\hat{\sigma }}}(\boldsymbol{x})^c} k_\alpha (|\boldsymbol{x} - \boldsymbol{y}|) |\rho ^{\varepsilon ,b} - \rho ^{\varepsilon }|(\boldsymbol{y}) {\, \mathrm d}\boldsymbol{y} \\&\quad \le \Vert k_\alpha (|\cdot |) \Vert _{L^\frac{q}{q - 1}(B_{3{\hat{\sigma }}})} \Vert \rho ^{\varepsilon ,b} - \rho ^{\varepsilon }\Vert _{L^q(B_{3{\hat{\sigma }}}(\boldsymbol{x}))} \\&\qquad \, + C \int _{B_{3{\hat{\sigma }}}(\boldsymbol{x})^c} \left( k_\alpha (1 + D^2) + k_\alpha (1 + |\boldsymbol{y}|^2) \right) |\rho ^{\varepsilon ,b} - \rho ^{\varepsilon }|(\boldsymbol{y}) {\, \mathrm d}\boldsymbol{y} \\&\quad \le C({\hat{\sigma }}) \Vert \rho ^{\varepsilon ,b} - \rho ^{\varepsilon }\Vert _{L^q(\mathbb {R}^n)} + C(D) \Vert \rho ^{\varepsilon ,b} - \rho ^{\varepsilon }\Vert _{L^1(\mathbb {R}^n)} \\&\qquad \, + C \int _{B_{3{\hat{\sigma }}}(\boldsymbol{x})^c} k_\alpha (1 + |\boldsymbol{y}|^2) \rho ^{\varepsilon }(\boldsymbol{y}) {\, \mathrm d}\boldsymbol{y} + C \int _{B_{3{\hat{\sigma }}}(\boldsymbol{x})^c} k_\alpha (1 + |\boldsymbol{y}|^2) \rho ^{\varepsilon ,b}(\boldsymbol{y}) {\, \mathrm d}\boldsymbol{y} \\&\quad =: I_1 + I_2 + I_3 + I_4. \end{aligned} \end{aligned}$$

By Lemma 4.7, for fixed ${\hat{\sigma }}$, we have

4.34 $$\begin{aligned} \sup _{[0,T] \times [0,D]} |I_1 + I_2| \longrightarrow 0 \qquad \text {as } b \rightarrow \infty . \end{aligned}$$

For $I_3$, by Lemma 4.6 and the Lebesgue dominated convergence theorem, we obtain

$$\begin{aligned} \sup _{[0,T] \times [0,D]} |I_3| \longrightarrow 0 \qquad \hbox {as } {\hat{\sigma }} \rightarrow \infty , \hbox { uniformly in } \, b. \end{aligned}$$

For $I_4$, by (4.33), we have

$$\begin{aligned} \begin{aligned} |I_4|&= C \int ^{b(t)}_{3{\hat{\sigma }}} k_\alpha (1 + r^2) \rho ^{\varepsilon ,b}(r) r^{n-1} {\, \mathrm d}r \\&\le C \int ^{b(t)}_0 \rho ^{\varepsilon ,b} (1\!\!1_{B_{2{\hat{\sigma }}}^c} * J_{\hat{\sigma }}) k_\alpha (1 + r^2) r^{n-1} {\, \mathrm d}r \\&= C \int ^{b(t)}_0 \rho ^{\varepsilon ,b}_0 (1\!\!1_{B_{2{\hat{\sigma }}}^c} * J_{\hat{\sigma }}) k_\alpha (1 + r^2) r^{n-1} {\, \mathrm d}r \\&\quad + C \int ^t_0 \int ^{b(s)}_0 \left( (1\!\!1_{B_{2{\hat{\sigma }}}^c} * J_{\hat{\sigma }}) (k_\alpha (1 + r^2))_r + (1\!\!1_{B_{2{\hat{\sigma }}}^c} * J_{\hat{\sigma }})_r k_\alpha (1 + r^2) \right) \rho ^{\varepsilon ,b} u^{\varepsilon ,b} r^{n-1} {\, \mathrm d}r {\, \mathrm d}s \\&\le C \int ^{\beta (t)}_{\hat{\sigma }} \rho ^{\varepsilon ,b}_0 k_ {\alpha }(1 + r^2) r^{n-1} {\, \mathrm d}r + \frac{C}{{\hat{\sigma }}^{\alpha + 1}} \int ^t_0 \int ^{b(s)}_ {\hat{\sigma }} \rho ^{\varepsilon ,b} u^{\varepsilon ,b} r^{n-1} {\, \mathrm d}r {\, \mathrm d}s \\&\quad + \frac{C k_\alpha (1 + {\hat{\sigma }}^2)}{{\hat{\sigma }}} \int ^t_0 \int ^{3{\hat{\sigma }}}_{\hat{\sigma }} \rho ^{\varepsilon ,b} u^{\varepsilon ,b} r^{n-1} {\, \mathrm d}r {\, \mathrm d}s \\&\le C \int ^{\infty }_{\hat{\sigma }} \rho ^{\varepsilon ,b}_0 k_\alpha (1 + r^2) r^{n-1} {\, \mathrm d}r + \frac{C k_\alpha (1 + {\hat{\sigma }}^2)}{{\hat{\sigma }}} \int ^t_0 \int ^{b(s)}_a \rho ^{\varepsilon ,b} u^{\varepsilon ,b} r^{n-1} {\, \mathrm d}r {\, \mathrm d}s \\&=: I_{4,1} + I_{4,2}. \end{aligned} \end{aligned}$$

By Lemma B.4 and the Lebesgue dominated convergence theorem, we obtain

4.35 $$\begin{aligned} \sup _{[0,T] \times [0,D]} |I_{4,1}| \longrightarrow 0 \qquad \hbox {as } {\hat{\sigma }} \rightarrow \infty ,\text { uniformly in } b. \end{aligned}$$

By Lemma 3.4 and the Cauchy-Schwarz inequality, we have

4.36 $$\begin{aligned} \sup _{[0,T] \times [0,D]} |I_{4,2}|&\le \frac{C k_\alpha (1 + {\hat{\sigma }}^2)}{{\hat{\sigma }}} \int ^t_0 \int ^{b(s)}_a \big (\rho ^{\varepsilon ,b} + \rho ^{\varepsilon ,b} (u^{\varepsilon ,b})^2\big ) r^{n-1} {\, \mathrm d}r {\, \mathrm d}s \nonumber \\&\le \frac{C(M,E_0,T) k_\alpha (1 + {\hat{\sigma }}^2)}{{\hat{\sigma }}} \longrightarrow 0 \qquad \text { as } {\hat{\sigma }} \rightarrow \infty , \text { uniformly in } b. \end{aligned}$$

Thus, combining (4.34)–(4.36) together yields that, for $\alpha \in (-1,0]$,5. For (4.20), it follows from Lemma 4.7 and (4.19) that, when $\alpha \in (-1,n - 1)$ and ${\hat{\sigma }} > 0$,

4.37 $$\begin{aligned} \int _0^{\hat{\sigma }} \big |\rho ^{\varepsilon ,b} (\Phi _\alpha *\rho ^{\varepsilon ,b}) - \rho ^{\varepsilon } (\Phi _\alpha *\rho ^{\varepsilon })\big |(t,r)\, r^{n-1} {\, \mathrm d}r \longrightarrow 0 \qquad \text {as } b\rightarrow \infty . \end{aligned}$$

When $\alpha \in (0,n-1)$, using (4.32), we have

4.38 $$\begin{aligned}&\int _{\hat{\sigma }}^\infty \big |\rho ^{\varepsilon ,b} (\Phi _\alpha *\rho ^{\varepsilon ,b})\big |(t,r)\, r^{n-1} {\, \mathrm d}r \nonumber \\&\quad \le C \int _{\hat{\sigma }}^\infty \rho ^{\varepsilon ,b}(t,r) \bigg ( \Big (\int _0^{r-1}+ \int _{r+1}^\infty \Big ) \rho ^{\varepsilon ,b}(t,\eta ) \eta ^{n-1} {\, \mathrm d}\eta \nonumber \\&\qquad \qquad \qquad \qquad \qquad \qquad + \int _{r-1}^{r+1} \frac{1}{(r \eta )^{\frac{\alpha }{2}}} \rho ^{\varepsilon ,b}(t,\eta ) \eta ^{n-1} {\, \mathrm d}\eta \bigg ) r^{n-1} {\, \mathrm d}r \nonumber \\&\quad \le C \int _{\hat{\sigma }}^\infty \rho ^{\varepsilon ,b}(t,r) \bigg ( \Big (\int _0^{r-1}+\int _{r+1}^\infty \Big ) \rho ^{\varepsilon ,b}(t,\eta ) \eta ^{n-1} {\, \mathrm d}\eta \nonumber \\&\qquad \qquad \qquad \qquad \qquad \qquad + \frac{1}{({\hat{\sigma }}-1)^{\alpha }} \int _{r-1}^{r+1} \rho ^{\varepsilon ,b}(t,\eta ) \eta ^{n-1} {\, \mathrm d}\eta \bigg ) r^{n-1} {\, \mathrm d}r\nonumber \\&\quad \le C(M) \int _{\hat{\sigma }}^\infty \rho ^{\varepsilon ,b}(t,r) r^{n-1} {\, \mathrm d}r. \end{aligned}$$

We can also replace $\rho ^{\varepsilon ,b}$ by $\rho ^{\varepsilon }$ in (4.38). Thus, for δ>0, with (4.14), (4.16), and (4.38), we obtain that, for *b* and ${\hat{\sigma }}$ sufficiently large,

4.39 $$\begin{aligned} \int _{\hat{\sigma }}^\infty \big |\rho ^{\varepsilon ,b} (\Phi _\alpha *\rho ^{\varepsilon ,b}) - \rho ^{\varepsilon } (\Phi _\alpha *\rho ^{\varepsilon })\big |(t,r)\, r^{n-1} {\, \mathrm d}r \le C(M) \delta . \end{aligned}$$

When $\alpha \in (-1,0]$, using (3.21), (3.23), and Lemma 4.6, we have

$$\begin{aligned} \begin{aligned}&\int _{\hat{\sigma }}^\infty |\rho ^{\varepsilon ,b} (\Phi _\alpha *\rho ^{\varepsilon ,b})|(t,r)\, r^{n-1} {\, \mathrm d}r \\&\quad = \frac{1}{\omega _n} \Big | \int _{B_{\hat{\sigma }}^c} \int _{\mathbb {R}^n} \rho ^{\varepsilon ,b}(\boldsymbol{x}) k_\alpha (|\boldsymbol{x} - \boldsymbol{y}|) \rho ^{\varepsilon ,b}(\boldsymbol{y}) {\, \mathrm d}\boldsymbol{x} {\, \mathrm d}\boldsymbol{y} \Big | \\&\quad \le \frac{1}{\omega _n} \int _{B_{\hat{\sigma }}^c} \rho ^{\varepsilon ,b}(\boldsymbol{y}) \Big ( \int _{B_1(\boldsymbol{y})} \rho ^{\varepsilon ,b}(\boldsymbol{x}) |k_\alpha (|\boldsymbol{x} - \boldsymbol{y}|)| {\, \mathrm d}\boldsymbol{x} + \int _{B_1(\boldsymbol{y})^c} \rho ^{\varepsilon ,b}(\boldsymbol{x}) k_\alpha (|\boldsymbol{x} - \boldsymbol{y}|) {\, \mathrm d}\boldsymbol{x} \Big ) {\, \mathrm d}\boldsymbol{y} \\&\quad \le \frac{1}{\omega _n} \int _{B_{\hat{\sigma }}^c} \rho ^{\varepsilon ,b}(\boldsymbol{y}) \Big ( \Vert \rho ^{\varepsilon ,b} \Vert _{L^\gamma (B_1(\boldsymbol{y}))} \Vert k_\alpha (|\cdot |) \Vert _{L^\frac{\gamma }{\gamma - 1}(B_1)} \\&\quad \qquad \qquad \qquad \qquad \qquad + C \int _{|\boldsymbol{x} - \boldsymbol{y}| \ge 1} \rho ^{\varepsilon ,b}(\boldsymbol{x}) \left( 1 + k_\alpha (1 + |\boldsymbol{x}|^2) + k_\alpha (1 + |\boldsymbol{y}|^2) \right) {\, \mathrm d}\boldsymbol{x} \Big ) {\, \mathrm d}\boldsymbol{y} \\&\quad \le C(M,E_0,T) \int _{B_{\hat{\sigma }}^c} \rho ^{\varepsilon ,b}(\boldsymbol{x}) \left( 1 + k_\alpha (1+|\boldsymbol{x}|^2) \right) {\, \mathrm d}\boldsymbol{x} \\&\quad \le C(M,E_0,T) \Big ( \Vert \rho ^{\varepsilon ,b} - \rho ^{\varepsilon }\Vert _{L^1(B_{\hat{\sigma }}^c)} + \int _{B_{\hat{\sigma }}^c} \rho ^{\varepsilon }(\boldsymbol{x}) {\, \mathrm d}\boldsymbol{x} + \int _{B_{\hat{\sigma }}^c} \rho ^{\varepsilon ,b}(\boldsymbol{x}) k_\alpha (1+|\boldsymbol{x}|^2) {\, \mathrm d}\boldsymbol{x} \Big ). \end{aligned} \end{aligned}$$

Notice that, by (4.35)–(4.36),

4.40 $$\begin{aligned} \sup _{[0,T]} \Big |\int _{B_{\hat{\sigma }}^c} \rho ^{\varepsilon ,b}(\boldsymbol{x}) k_\alpha (1+|\boldsymbol{x}|^2) {\, \mathrm d}\boldsymbol{x} \Big | \longrightarrow 0 \qquad \text {as } \hat{\sigma } \rightarrow \infty \,\, \text {uniformly in } b. \end{aligned}$$

Thus, for δ>0, by Lemmas 4.6–4.7, the Lebesgue dominated convergence theorem, and (4.40), we obtain that, for *b* and ${\hat{\sigma }}$ sufficiently large,

4.41 $$\begin{aligned} \int _{\hat{\sigma }}^\infty \big |\rho ^{\varepsilon ,b} (\Phi _\alpha *\rho ^{\varepsilon ,b}) - \rho ^{\varepsilon } (\Phi _\alpha *\rho ^{\varepsilon })\big |(t,r)\, r^{n-1} {\, \mathrm d}r \le C(M,E_0,T) \delta . \end{aligned}$$

Therefore, combining (4.37) and (4.39) with (4.41) leads to (4.20) for $\alpha \in (-1,n-1)$. □

### Lemma 4.9

Under the assumptions of Lemma 4.1, the following holds:

$$\begin{aligned}&\int _0^\infty \Big (\frac{1}{2} \Big |\frac{m^{\varepsilon }}{\sqrt{\rho ^\varepsilon }} \Big |^2 + \rho ^{\varepsilon }e(\rho ^\varepsilon ) + \frac{\kappa }{2} \rho ^\varepsilon (\Phi _\alpha * \rho ^\varepsilon )\Big )(t,r)\,r^{n-1}{\, \mathrm d}r \\&\quad \le \int _0^\infty \Big (\frac{1}{2} \Big |\frac{m^{\varepsilon }_0}{\sqrt{\rho ^\varepsilon _0}}\Big |^2 + \rho ^{\varepsilon }_0 e(\rho ^{\varepsilon }_0) + \frac{\kappa }{2} \rho ^\varepsilon _0 (\Phi _\alpha * \rho ^\varepsilon _0)\Big )(r)\,r^{n-1}{\, \mathrm d}r. \end{aligned}$$

### Proof

It follows from Lemma 3.4 that

$$\begin{aligned}&\int _0^{b(t)}\Big (\frac{1}{2} \Big |\frac{m^{\varepsilon ,b}}{\sqrt{\rho ^{\varepsilon ,b}}}\Big |^2 + \rho ^{\varepsilon ,b} e(\rho ^{\varepsilon ,b}) + \frac{\kappa }{2} \rho ^{\varepsilon ,b} \big (\Phi _\alpha * \rho ^{\varepsilon ,b}\big )\Big )(t,r)\,r^{n-1}{\, \mathrm d}r \\&\quad \le \int _0^{b(t)} \Big (\frac{1}{2} \Big |\frac{m^{\varepsilon ,b}_0}{\sqrt{\rho ^{\varepsilon ,b}_0}}\Big |^2 + \rho ^{\varepsilon ,b}_0 e(\rho ^{\varepsilon ,b}_0) + \frac{\kappa }{2} \rho ^{\varepsilon ,b}_0\big (\Phi _\alpha * \rho ^{\varepsilon ,b}_0\big ) \Big )(r)\,r^{n-1}{\, \mathrm d}r. \end{aligned}$$

Using the construction of the approximation of $(\rho ^\varepsilon _0,m^\varepsilon _0)$ and Lemma 3.8, we have

$$\begin{aligned}&\int _0^{b(t)} \Big (\frac{1}{2} \Big |\frac{m^{\varepsilon ,b}_0}{\sqrt{\rho ^{\varepsilon ,b}_0}}\Big |^2 + \rho ^{\varepsilon ,b}_0 e(\rho ^{\varepsilon ,b}_0) + \frac{\kappa }{2} \rho ^{\varepsilon ,b}_0 (\Phi _\alpha * \rho ^{\varepsilon ,b}_0)\Big )(r)\, r^{n-1}{\, \mathrm d}r \\&\longrightarrow \int _0^\infty \Big (\frac{1}{2} \Big |\frac{m^{\varepsilon }_0}{\sqrt{\rho ^\varepsilon _0}}\Big |^2 + \rho ^{\varepsilon }_0 e(\rho ^{\varepsilon }_0) + \frac{\kappa }{2} \rho ^{\varepsilon }_0 (\Phi _\alpha * \rho ^\varepsilon _0)\Big )(r)\,r^{n-1}{\, \mathrm d}r \qquad \text {as } b \rightarrow \infty . \end{aligned}$$

Now, by the Fatou lemma, Lemma 3.8, and (4.20), we obtain

$$\begin{aligned}&\int _0^\infty \Big (\frac{1}{2} \Big |\frac{m^{\varepsilon }}{\sqrt{\rho ^\varepsilon }}\Big |^2 + \rho ^{\varepsilon } e(\rho ^{\varepsilon }) + \frac{\kappa }{2} \rho ^{\varepsilon } (\Phi _\alpha * \rho ^\varepsilon )\Big )(t,r)\,r^{n-1}{\, \mathrm d}r \\&\quad \le \liminf _{b \rightarrow \infty } \int _0^{b(t)} \Big (\frac{1}{2} \Big |\frac{m^{\varepsilon ,b}}{\sqrt{\rho ^{\varepsilon ,b}}} \Big |^2 + \rho ^{\varepsilon ,b} e(\rho ^{\varepsilon ,b}) + \frac{\kappa }{2} \rho ^{\varepsilon ,b} \big (\Phi _\alpha * \rho ^{\varepsilon ,b}\big )\Big )(t,r)\,r^{n-1}{\, \mathrm d}r. \end{aligned}$$

This completes the proof. □

### Lemma 4.10

Under the assumptions of Lemma 4.1, let $\boldsymbol{\psi }(t,\boldsymbol{x})\in \left( C^2_0([0,T]\times \mathbb {R}^n)\right) ^n$ be any smooth function with compact support so that $\boldsymbol{\psi }(T,\boldsymbol{x})=0$. Then

$$\begin{aligned}&\int _{\mathbb {R}_+^{n+1}} \Big \{{\mathcal {M}}^{\varepsilon } \cdot \partial _t\boldsymbol{\psi }+\frac{{\mathcal {M}}^{\varepsilon }}{\sqrt{\rho ^{\varepsilon }}} \cdot \big (\frac{{\mathcal {M}}^{\varepsilon }}{\sqrt{\rho ^{\varepsilon }}}\cdot \nabla \big )\boldsymbol{\psi }+p(\rho ^{\varepsilon }) \nabla \cdot \boldsymbol{\psi }-\kappa \rho ^{\varepsilon } \nabla (\Phi _\alpha * \rho ^\varepsilon )\cdot \boldsymbol{\psi }\Big \}\,{\, \mathrm d}\boldsymbol{x} {\, \mathrm d}t\\&\qquad +\int _{\mathbb {R}^n} {\mathcal {M}}_0^\varepsilon \cdot \boldsymbol{\psi }(0,\boldsymbol{x})\,{\, \mathrm d}\boldsymbol{x} \\&\quad =-\varepsilon \int _{\mathbb {R}_+^{n+1}} \Big \{\frac{1}{2}{\mathcal {M}}^{\varepsilon }\cdot \big (\Delta \boldsymbol{\psi }+\nabla (\nabla \cdot \,\boldsymbol{\psi }) \big ) + \frac{{\mathcal {M}}^{\varepsilon }}{\sqrt{\rho ^{\varepsilon }}} \cdot \big (\nabla \sqrt{\rho ^{\varepsilon }}\cdot \nabla \big )\boldsymbol{\psi }+ \nabla \sqrt{\rho ^{\varepsilon }} \cdot \big (\frac{{\mathcal {M}}^{\varepsilon }}{\sqrt{\rho ^{\varepsilon }}}\cdot \nabla \big )\boldsymbol{\psi }\Big \}\,{\, \mathrm d}\boldsymbol{x}{\, \mathrm d}t \\&\quad =\sqrt{\varepsilon }\int _{\mathbb {R}_+^{n+1}} \sqrt{\rho ^{\varepsilon }} \Big \{V^{\varepsilon } \frac{\boldsymbol{x}\otimes \boldsymbol{x}}{r^2} +\frac{\sqrt{\varepsilon }}{r}\frac{m^\varepsilon }{\sqrt{\rho ^\varepsilon }}\big (I_{n\times n}-\frac{\boldsymbol{x}\otimes \boldsymbol{x}}{r^2}\big )\Big \}: \nabla \boldsymbol{\psi }\, {\, \mathrm d}\boldsymbol{x}{\, \mathrm d}t, \end{aligned}$$

where $V^{\varepsilon }(t,r)\in L^2(0,T; L^2(\mathbb {R}^n))$ is a function such that

$$ \displaystyle \int _0^T\int _{\mathbb {R}^n} |V^{\varepsilon }(t,\boldsymbol{x})|^2\, {\, \mathrm d}\boldsymbol{x}{\, \mathrm d}t\le C(E_0,M) \quad \text {for some } C(E_0,M)>0 \text { independent of } T>0. $$

The proof of this lemma is the same as the argument for that of [26].

### Lemma 4.11

($H_\textrm{loc}^{-1}$–Compactness). Under the assumptions of Lemma 4.1, let (η,q) be a weak entropy pair defined in (3.63) for any compactly supported smooth function ψ(s) in $\mathbb {R}$. Then

$$\begin{aligned} \partial _t\eta (\rho ^\varepsilon ,m^\varepsilon )+\partial _rq(\rho ^\varepsilon ,m^\varepsilon ) \qquad \text {is compact in } H^{-1}_\textrm{loc}(\mathbb {R}^2_+). \end{aligned}$$

### Proof

We multiply $(2.10)_1$ by $\eta _\rho ^{\varepsilon ,b} {:=}\eta _\rho (\rho ^{\varepsilon ,b},m^{\varepsilon ,b})$ and $(2.10)_2$ by $\eta _m^{\varepsilon ,b} {:=}\eta _m(\rho ^{\varepsilon ,b},m^{\varepsilon ,b})$. Then, by the construction of the entropy pair, we obtain

4.42 $$\begin{aligned} \partial _t \eta ^{\varepsilon ,b} + \partial _r q^{\varepsilon ,b} =&- \frac{n-1}{r}m^{\varepsilon ,b}(\eta _\rho ^{\varepsilon ,b} + u^{\varepsilon ,b} \eta _m^{\varepsilon ,b}) - \kappa \eta ^{\varepsilon ,b}_m \rho ^{\varepsilon ,b} (\Phi _\alpha * \rho ^{\varepsilon ,b})_r \nonumber \\&+ \varepsilon \eta _m^{\varepsilon ,b} \Big \{ \Big ( \rho ^{\varepsilon ,b}(u^{\varepsilon ,b}_r + \frac{n-1}{r} u^{\varepsilon ,b})\Big )_r - \frac{n-1}{r} \rho _r^{\varepsilon ,b} u^{\varepsilon ,b} \Big \}. \end{aligned}$$

Multiplying (4.42) by $\phi (t,r) \in C^\infty _0(\mathbb {R}^2_+)$ and integrating it over $\mathbb {R}^2_+$, and then integrating by parts, we obtain

$$\begin{aligned} \displaystyle&\int _{\mathbb {R}^2_+} \big (\partial _t \eta ^{\varepsilon ,b} + \partial _r q^{\varepsilon ,b}\big )\phi {\, \mathrm d}r{\, \mathrm d}t \\ \displaystyle&\quad = - \int _{\mathbb {R}^2_+} \frac{n-1}{r}m^{\varepsilon ,b} \big (\eta _\rho ^{\varepsilon ,b} + u^{\varepsilon ,b} \eta _m^{\varepsilon ,b}\big ) \phi {\, \mathrm d}r {\, \mathrm d}t - \varepsilon \int _{\mathbb {R}^2_+} \big (\eta _m^{\varepsilon ,b})_r \rho ^{\varepsilon ,b}(u^{\varepsilon ,b}_r + \frac{n-1}{r} u^{\varepsilon ,b}\big ) \phi {\, \mathrm d}r {\, \mathrm d}t \\ \displaystyle&\qquad - \varepsilon \int _{\mathbb {R}^2_+} \eta _m^{\varepsilon ,b} \rho ^{\varepsilon ,b}\big (u^{\varepsilon ,b}_r + \frac{n-1}{r} u^{\varepsilon ,b}\big ) \phi _r {\, \mathrm d}r {\, \mathrm d}t - \varepsilon \int _{\mathbb {R}^2_+} \eta _m^{\varepsilon ,b} \frac{n-1}{r} \rho _r^{\varepsilon ,b} u^{\varepsilon ,b}\phi {\, \mathrm d}r {\, \mathrm d}t \\ \displaystyle&\qquad - \kappa \int _{\mathbb {R}^2_+} \eta ^{\varepsilon ,b}_m \rho ^{\varepsilon ,b} \big (\Phi _\alpha * \rho ^{\varepsilon ,b}\big )_r \phi {\, \mathrm d}r {\, \mathrm d}t {:=}\sum ^5_{i=1} I^{\varepsilon ,b}_i. \end{aligned}$$

Notice that the terms, $I^{\varepsilon ,b}_i$ for i=1,⋯,4, are the same as in [26, Lemma 4.12], so that they can be handled in the same ways. However, $I^{\varepsilon ,b}_5$ is different and, as such, requires another approach.

Since ϕ is compactly supported in $\mathbb {R}^2_+$, there exist $(d,D) \Subset (0,\infty )$ and T>0 such that $\textrm{supp}(\phi ) \subset (0,T) \times (d,D)$ so that

4.43 $$\begin{aligned} |I^{\varepsilon ,b}_5|&\le C\int ^T_0 \int _d^D \Big | \eta ^{\varepsilon ,b}_m \rho ^{\varepsilon ,b} (\Phi _\alpha * \rho ^{\varepsilon ,b})_r \Big | {\, \mathrm d}r {\, \mathrm d}t\nonumber \\&\le C_\psi \int ^T_0 \int _d^D \rho ^{\varepsilon ,b}(t, r) \int ^{b(t)}_a \big |\omega (r,\eta )\big | \rho ^{\varepsilon ,b}(t, \eta ) \eta ^{n-1} {\, \mathrm d}\eta {\, \mathrm d}r {\, \mathrm d}t. \end{aligned}$$

We need to prove the following estimate:

4.44 $$\begin{aligned} \int ^T_0 \int _d^D \big |\eta ^{\varepsilon ,b}_m \rho ^{\varepsilon ,b} (\Phi _\alpha * \rho ^{\varepsilon ,b})_r \big |{\, \mathrm d}r {\, \mathrm d}t \le C_\psi (d,D,M,E_0,T). \end{aligned}$$

When $\alpha \in (n-2,n-1)$, combining (4.43) with (3.1) and using the Fubini lemma, we have

4.45 $$\begin{aligned}&\int ^T_0 \int _d^D \left| \eta ^{\varepsilon ,b}_m \rho ^{\varepsilon ,b} (\Phi _\alpha * \rho ^{\varepsilon ,b})_r \right| {\, \mathrm d}r {\, \mathrm d}t \nonumber \\&\le C_\psi \int ^T_0 \int _d^D \rho ^{\varepsilon ,b}(t,r) \int _{(a,b(t))\cap (r-\frac{d}{2},r + \frac{d}{2})}|\omega (r,\eta )| \rho ^{\varepsilon ,b}(t, \eta ) \eta ^{n-1} {\, \mathrm d}\eta {\, \mathrm d}r {\, \mathrm d}t \nonumber \\&\quad \, + C_\psi \int ^T_0 \int _d^D \rho ^{\varepsilon ,b}(t, r) \int _{(a,b(t))\cap (r-\frac{d}{2},r + \frac{d}{2})^c}|\omega (r,\eta )| \rho ^{\varepsilon ,b}(t, \eta ) \eta ^{n-1} {\, \mathrm d}\eta {\, \mathrm d}r {\, \mathrm d}t\nonumber \\&\le C_\psi \int ^T_0 \int _d^D \rho ^{\varepsilon ,b}(t, r) \int _{(a,b(t))\cap (r-\frac{d}{2},r + \frac{d}{2})} \frac{|r - \eta |^{n-2-\alpha }}{(r \eta )^{\frac{n-1}{2}}} \rho ^{\varepsilon ,b}(t, \eta ) \eta ^{n-1} {\, \mathrm d}\eta {\, \mathrm d}r {\, \mathrm d}t \nonumber \\&\quad \, + C_\psi (d) \int ^T_0 \int _d^D \rho ^{\varepsilon ,b}(t, r) \int _{(a,b(t))\cap (r-\frac{d}{2},r + \frac{d}{2})^c} \rho ^{\varepsilon ,b}(t,\eta ) \eta ^{n-1} {\, \mathrm d}\eta {\, \mathrm d}r {\, \mathrm d}t\nonumber \\&\le C_\psi (d,M,T)\nonumber \\&\quad + C_\psi \int ^T_0 \int _d^D \rho ^{\varepsilon ,b}(t,r) \int _{(a,b(t))\cap (r-\frac{d}{2},r + \frac{d}{2})} \frac{|r - \eta |^{n-2-\alpha }}{d^{n-1}} \rho ^{\varepsilon ,b}(t,\eta ) \eta ^{n-1} {\, \mathrm d}\eta {\, \mathrm d}r {\, \mathrm d}t \nonumber \\&\le C_\psi (d,M,T) + C_\psi (d) \int ^T_0 \int ^{b(t)}_a \rho ^{\varepsilon ,b}(t,\eta ) \eta ^{n-1} \int _d^D |r - \eta |^{n-2-\alpha } \rho ^{\varepsilon ,b}(t,r) {\, \mathrm d}r {\, \mathrm d}\eta {\, \mathrm d}t. \end{aligned}$$

Thus, for $\gamma > \frac{1}{(n-1) - \alpha }$, using Theorem A.1 and (4.45), we obtain

$$\begin{aligned}&\int ^T_0 \int _d^D \big | \eta ^{\varepsilon ,b}_m \rho ^{\varepsilon ,b} (\Phi _\alpha * \rho ^{\varepsilon ,b})_r \big |{\, \mathrm d}r {\, \mathrm d}t \\&\le C_\psi \int ^T_0 \int ^{b(t)}_a \rho ^{\varepsilon ,b}(t,\eta ) \eta ^{n-1} \sup _{z \in \mathbb {R}_+} \Big \{ \int _d^D |r - z|^{n-2-\alpha } \rho ^{\varepsilon ,b}(t, r) {\, \mathrm d}r \Big \} {\, \mathrm d}\eta {\, \mathrm d}t \\&= C_\psi \int ^T_0 \int ^{b(t)}_a \rho ^{\varepsilon ,b}(t,\eta ) \eta ^{n-1} \sup _{z \in [d,D]} \Big \{ \int _d^D |r - z|^{n-2-\alpha } \rho ^{\varepsilon ,b}(t, r) {\, \mathrm d}r \Big \} {\, \mathrm d}\eta {\, \mathrm d}t \\&\le C_\psi (K) \Vert \rho ^{\varepsilon ,b} \Vert _{L^\gamma (d,D)} \int ^T_0 \int ^{b(t)}_a \rho ^{\varepsilon ,b}(t, \eta ) \eta ^{n-1} {\, \mathrm d}\eta {\, \mathrm d}t \\&\le C_\psi (d,D,M,E_0,T). \end{aligned}$$

When $\alpha \in (-1,n-2]$, using the approach to (4.45) but instead with the alternate estimate for ω as in (3.1) for $\alpha \in (-1,n-2]$, we obtain (4.44). □

Thus, to sum up, we have the following corollary.

### Corollary 4.12

Under the assumptions of Lemma 4.1, for any T>0, there exists a global spherically symmetric solution $(\rho ^\varepsilon , m^\varepsilon )=(\rho ^\varepsilon , \rho ^\varepsilon u^\varepsilon )$ of CNSREs (1.6) in the sense of Definition 2.3 satisfying

$$\begin{aligned} \begin{aligned}&\rho ^{\varepsilon }(t,r)\ge 0 \,\,\,\, a.e.,\nonumber \\&u^\varepsilon (t,r)=0, \,\,\big (\frac{m^\varepsilon }{\sqrt{\rho ^\varepsilon }}\big )(t,r)=\sqrt{\rho ^\varepsilon }(t,r)u^\varepsilon (t,r)=0 \quad \, a.e.\,\, \text {on } \{(t,r)\,:\, \rho ^\varepsilon (t,r)=0\},\nonumber \\&\int ^\infty _0 \rho ^{\varepsilon }(t,r) r^{n-1} {\, \mathrm d}r = \frac{M}{\omega _n} \qquad \text {for all } t\in [0,T],\nonumber \\&\int _0^\infty \Big (\frac{1}{2}\rho ^{\varepsilon } |u^{\varepsilon }|^2+ \rho ^{\varepsilon }e(\rho ^\varepsilon ) - \frac{\kappa }{2}\rho ^\varepsilon (\Phi _\alpha * \rho ^\varepsilon )\Big )(t,r)\,r^{n-1}{\, \mathrm d}r\\&\qquad +\varepsilon \int _0^t\int _0^\infty (\rho ^{\varepsilon } |u^{\varepsilon }|^2)(s,r)\, r^{n-3} {\, \mathrm d}r {\, \mathrm d}s \nonumber \\&\quad \le C(M,E_0) \qquad \text {for all } t\in [0,T],\nonumber \\&\int _0^\infty \bigg (\frac{1}{2} \Big |\frac{m^{\varepsilon }}{\sqrt{\rho ^\varepsilon }} \Big |^2 + \rho ^{\varepsilon }e(\rho ^\varepsilon ) + \frac{\kappa }{2} \rho ^\varepsilon (\Phi _\alpha * \rho ^\varepsilon )\bigg )(t,r)\,r^{n-1}{\, \mathrm d}r \nonumber \\&\quad \le \int _0^\infty \bigg (\frac{1}{2} \Big |\frac{m^{\varepsilon }_0}{\sqrt{\rho ^\varepsilon _0}} \Big |^2 + \rho ^{\varepsilon }_0 e(\rho ^{\varepsilon }_0) + \frac{\kappa }{2} \rho ^\varepsilon _0 (\Phi _\alpha * \rho ^\varepsilon _0)\bigg )(r)\,r^{n-1}{\, \mathrm d}r \qquad \text {for all } t\in [0,T],\nonumber \\&\varepsilon ^2 \int _0^\infty \big |(\sqrt{\rho ^{\varepsilon }(t,r)})_r\big |^2\,r^{n-1}{\, \mathrm d}r +\varepsilon \int _0^T\int _{0}^\infty \big |\big ((\rho ^{\varepsilon }(s,r))^{\frac{\gamma }{2}}\big )_r\big |^2\,r^{n-1}{\, \mathrm d}r{\, \mathrm d}s\nonumber \\&\le C(M,E_0,T) \qquad \text{ for } \text{ all } t\in [0,T] ,\nonumber \\ {\begin{matrix} & \int _0^T\int _d^D \big (\rho ^{\varepsilon }|u^{\varepsilon }|^3+(\rho ^{\varepsilon })^{\gamma +\theta }+(\rho ^{\varepsilon })^{\gamma +1}\big )(t,r)\, r^{n-1}{\, \mathrm d}r {\, \mathrm d}t\\ & \quad \le C(d,D,M,E_0,T) \qquad \text{ for } \text{ all } t\in [0,T] , \end{matrix}} \nonumber \end{aligned} \end{aligned}$$

where $[d,D]\Subset (0,\infty )$. Let (η,q) be a weak entropy pair defined in (3.63) for any smooth compact supported function ψ(s) on $\mathbb {R}$. Then, for $\varepsilon \in (0, \varepsilon _0]$,

$$\begin{aligned} \partial _t\eta (\rho ^\varepsilon ,m^\varepsilon )+\partial _rq(\rho ^\varepsilon ,m^\varepsilon ) \qquad \text {is compact in } H^{-1}_\textrm{loc}(\mathbb {R}^2_+). \end{aligned}$$

As in [26], similar to the compensated compactness argument in [27], we can derive that there exists a vector function (ρ,m)(t,r) such that

$$\begin{aligned} (\rho ^\varepsilon ,m^{\varepsilon })\longrightarrow (\rho ,m) \qquad { a.e.} \,(t,r)\in \mathbb {R}^2_+\,\text { as } \varepsilon \rightarrow 0^+\text {(up to a sub-sequence)}. \end{aligned}$$

Notice that m(t,r)=0 *a.e.* on $\{(t,r):\, \rho (t,r)=0\}$. We can define the limit velocity *u*(*t*, *r*) by setting $u(t,r){:=}\frac{m(t,r)}{\rho (t,r)}$ *a.e.* on $\{(t,r):\,\rho (t,r)\ne 0\}$ and $u(t,r){:=}0$ *a.e.* on $\{(t,r): \rho (t,r)=0\,\,\, \text {or }$r=0}. Then

$$\begin{aligned} m(t,r)=\rho (t,r) u(t,r). \end{aligned}$$

We can also define $(\frac{m}{\sqrt{\rho }})(t,r){:=}\sqrt{\rho (t,r)} u(t,r)$, which is 0 *a.e.* on $\{(t,r) \,\ \rho (t,r)=0\}$. Moreover, we obtain that, as $\varepsilon \rightarrow 0^+$,

$$\begin{aligned} \frac{m^{\varepsilon }}{\sqrt{\rho ^{\varepsilon }}}\equiv \sqrt{\rho ^{\varepsilon }} u^{\varepsilon }\longrightarrow \frac{m}{\sqrt{\rho }}\equiv \sqrt{\rho } u \qquad \text {strongly in } L^2([0,T]\times [d,D], r^{n-1}{\, \mathrm d}r {\, \mathrm d}t) \end{aligned}$$

for any given *T* and $[d,D]\Subset (0,\infty )$, and

4.46 $$\begin{aligned} (\rho ^\varepsilon ,m^{\varepsilon })\longrightarrow (\rho ,m) \quad \text {in } L^{p_1}_\textrm{loc}(\mathbb {R}^2_+)\times L^{p_2}_\textrm{loc}(\mathbb {R}^2_+) \end{aligned}$$

for $$p_1\in [1,\gamma +1)$$ and $$p_2\in [1,\frac{3(\gamma +1)}{\gamma +3})$$.

Integrating (4.7) over [0, *T*], letting $\varepsilon \rightarrow 0^+$ (up to a sub-sequence), and using (4.46), we obtain

4.47 $$\begin{aligned} \int ^T_0 \int _{\mathbb {R}^n} \rho (t,\boldsymbol{x}) \zeta (t,\boldsymbol{x})\, {\, \mathrm d}\boldsymbol{x} {\, \mathrm d}t&= \int ^T_0 \int _{\mathbb {R}^n} \rho _0(\boldsymbol{x}) \zeta (0,\boldsymbol{x})\, {\, \mathrm d}\boldsymbol{x} {\, \mathrm d}t \nonumber \\&\quad + \int ^T_0 \int _{0}^{t} \int _{\mathbb {R}^n} \big (\rho \zeta _t + {\mathcal {M}}\cdot \nabla \zeta \big )\,{\, \mathrm d}\boldsymbol{x}{\, \mathrm d}s {\, \mathrm d}t \end{aligned}$$

for any $\zeta \in C^1_c([0,T] \times \mathbb {R}^n)$. Thus, in the same way as for Lemma 4.5, using (4.47), we can obtain

$$\begin{aligned} \int ^T_0 \int _{\mathbb {R}^n} \rho (t,\boldsymbol{x}) \, {\, \mathrm d}\boldsymbol{x} {\, \mathrm d}t = MT. \end{aligned}$$

Then, in the same way as in the proof of Lemma 4.8, we have

### Lemma 4.13

Under the assumptions of Theorem 2.5, the following statements hold as $\varepsilon \rightarrow 0$ (up to a sub-sequence):

$$\begin{aligned}&\Phi _\alpha *\rho ^{\varepsilon } \mathrm - \!\!\! \rightharpoonup \Phi _\alpha *\rho \\&\qquad \text {weak-} *\text { in } L^\infty (0,T; W^{1,\frac{2n}{\alpha +2}}_\textrm{loc}(\mathbb {R}^n)) \text { and weakly in } L^2(0,T; W^{1,\frac{2n}{\alpha +2}}_\textrm{loc}(\mathbb {R}^n)),\\&\int ^T_0 \int _0^\infty \big |\rho ^{\varepsilon } (\Phi _\alpha *\rho ^{\varepsilon })-\rho (\Phi _\alpha *\rho )\big |\, r^{n-1} {\, \mathrm d}r {\, \mathrm d}t \longrightarrow 0. \end{aligned}$$

In addition, for $\alpha \in (0,n-1)$,

$$\begin{aligned} \Vert (\Phi _\alpha *\rho )(t)\Vert _{L^{\frac{2n}{\alpha }}(\mathbb {R}^n)}+\Vert (\nabla \Phi _\alpha *\rho )(t)\Vert _{L^\frac{2n}{\alpha + 2}(\mathbb {R}^n)}\le C(M,E_0)\,\qquad \text { for } t\ge 0; \end{aligned}$$

and, for $\alpha \in (-1,0]$, for any $K \Subset \mathbb {R}^n$,

$$\begin{aligned} \Vert (\Phi _\alpha *\rho )(t)\Vert _{L^\infty (K)}+\Vert (\nabla \Phi _\alpha *\rho )(t)\Vert _{L^\frac{2n}{\alpha + 2}(K)}\le C(M,E_0,T,K)\qquad \,\text {for } t\ge 0. \end{aligned}$$

Thus, taking advantage of the construction of the initial data $(\rho _0^\varepsilon ,m^\varepsilon _0)$ and applying the results and approach of Lemma 4.9, we can obtain the classical energy inequality:

$$\begin{aligned}&\int _0^\infty \Big (\frac{1}{2} \Big |\frac{m}{\sqrt{\rho }} \Big |^2 + \rho e(\rho ) + \frac{\kappa }{2} \rho (\Phi _\alpha * \rho )\Big )(t,r)\,r^{n-1}{\, \mathrm d}r \\&\quad \le \int _0^\infty \Big (\frac{1}{2} \Big |\frac{m_0}{\sqrt{\rho _0}}\Big |^2 + \rho _0 e(\rho _0) + \frac{\kappa }{2} \rho _0 (\Phi _\alpha * \rho _0)\Big )(r)\,r^{n-1}{\, \mathrm d}r. \end{aligned}$$

Then, similar to [26], we can conclude that (ρ,m)(t,r) defines a global finite-energy solution of CEREs (1.1) in the sense of Definition 2.1, which leads to Theorem 2.5.

## Nonlinear Stability of Steady States for the Compressible Euler–Riesz Equations

In this section, we consider the attractive case (κ=1), $\alpha \in (0,n-1)$, and $\gamma > \frac{n + \alpha }{n}$, and assume that the initial data $(\rho _0,\mathcal {M}_0)(\boldsymbol{x})$ have finite initial mass and energy. For 0<M<∞, we recall the definition for a set of functions ϱ:

$$\begin{aligned} X_M {:=}\bigg \{ \varrho \in L^1_+(\mathbb {R}^n) \cap L^\frac{n + \alpha }{n}(\mathbb {R}^n) \,\, : \, \int _{\mathbb {R}^n} \varrho (\boldsymbol{x})\,{\, \mathrm d}\boldsymbol{x} = M \bigg \}. \end{aligned}$$

For $\varrho \in X_M,$ we recall the definition of the energy functional $\mathcal {G}$:

$$\begin{aligned} \mathcal {G}(\varrho ){:=}\int _{\mathbb {R}^n} \Big ((\varrho e(\varrho ))(\boldsymbol{x})+\frac{1}{2}\varrho (\boldsymbol{x})(\Phi _\alpha *\varrho )(\boldsymbol{x})\Big )\,{\, \mathrm d}\boldsymbol{x}. \end{aligned}$$

We first focus on proving the existence, uniqueness, regularity, and other properties of global minimizers of the free energy $\mathcal {G}$ on the set $X_M$. We include some details for the sake of completeness and clarity of exposition.

We notice that one can show that the global minimizers of the functional $\mathcal {G}$ in $X_M$, if they exist, are actually radially symmetric. This is a very classical result that goes back to [4] by using symmetric decreasing rearrangement via Lemma A.4. The following result on the existence of global minimizers is proven in [17, Theorem 5].

### Theorem 5.1

For $\alpha \in (0,n)$ with $\gamma > \frac{n + \alpha }{n}$, there exists a global minimizer $\tilde{\rho } \in X_M$ to $\mathcal {G}$. Moreover, any global minimizer is radially decreasing, compactly supported, and bounded, which is a stationary solution in the sense of Definition 2.4. Furthermore, any global minimizer satisfies the Euler-Lagrange conditions: There exists a constant (Lagrangian multiplier) λ such that

$$ {\left\{ \begin{array}{ll} \frac{{\, \mathrm d}}{{\, \mathrm d}\rho }(\rho e(\rho ))|_{\rho =\tilde{\rho }} +\Phi _\alpha *\tilde{\rho } =\lambda &  \text { for } \boldsymbol{x}\in \Gamma ,\\ (\Phi _\alpha *\tilde{\rho })(\boldsymbol{x})\ge \lambda &  \text { for } \boldsymbol{x}\in \mathbb {R}^n{\setminus }\Gamma , \end{array}\right. } $$

where $$\Gamma =\{\boldsymbol{x}\in \mathbb {R}^n:\, \tilde{\rho }(\boldsymbol{x})>0\}$$.

We need some parts of the core of the existence result of global minimizers in order to derive our stability estimates for CEREs. We sketch partially the proof making emphasis only on the relevant elements. The existence part of the proof follows the classical concentration compactness argument introduced by Lions [72] in the following result:

### Theorem 5.2

([72, Theorem II.1]). Suppose that5.1and

5.2 $$\begin{aligned} {\left\{ \begin{array}{ll} \Pi :[0,\infty ) \rightarrow [0,\infty ) \text { strictly convex,} \\ \lim _{t \rightarrow 0^+} \Pi (t)t^{-1} = 0, \\ \lim _{t \rightarrow \infty } \Pi (t)t^{-q} = \infty , \end{array}\right. } \end{aligned}$$

where $$q = \frac{p + 1}{p}$$. Define

$$\begin{aligned} \mathcal {A}_M {:=}\bigg \{ \varrho \in L^1_+(\mathbb {R}^n) \cap L^q(\mathbb {R}^n) \, : \int _{\mathbb {R}^n} \varrho (\boldsymbol{x})\, {\, \mathrm d}\boldsymbol{x} = M \bigg \}, \end{aligned}$$

and the energy functional as follows:

5.3 $$\begin{aligned} \mathcal {F}(\varrho )=\int _{\mathbb {R}^n}\Big (\Pi (\varrho (\boldsymbol{x})) - \frac{1}{2}\varrho (\boldsymbol{x})(\Psi *\varrho )(\boldsymbol{x})\Big )\,{\, \mathrm d}\boldsymbol{x}. \end{aligned}$$

Define the minimum of $\mathcal {F}$ over $\mathcal {A}_M$ by

$$\begin{aligned} F_M {:=}\inf _{\varrho \in \mathcal {A}_M} \mathcal {F}(\varrho ). \end{aligned}$$

Then, for any minimizing sequence $\{\rho ^j\} \subset \mathcal {A}_M$, there exist $\boldsymbol{y}_j \in \mathbb {R}^n$ and a minimizer $\tilde{\rho } \in \mathcal {A}_M$ to $\mathcal {F}$ (i.e., $\mathcal {F}(\tilde{\rho }) = F_M$) with $\{T^{\boldsymbol{y}_j} \rho ^j\}$ compact in $L^1(\mathbb {R}^n) \cap L^q(\mathbb {R}^n)$ such that $T^{\boldsymbol{y}_j} \rho ^j \rightarrow \tilde{\rho }$ in $L^1(\mathbb {R}^n) \cap L^q(\mathbb {R}^n)$ if and only if

5.4 $$\begin{aligned} F_M < F_m + F_{M - m} \qquad \text { for all}\quad m \in (0,M). \end{aligned}$$

We can now use this theorem in our problem since $-\Phi _\alpha \in L^\frac{n}{\alpha }_\textrm{w}(\mathbb {R}^n)$ and satisfies (5.1). Thus, we take $p = \frac{n}{\alpha }$, $q = \frac{p + 1}{p} = \frac{n + \alpha }{n}$, and the internal energy function $\Pi (\tau ) {:=}\tau e(\tau ) = \frac{a_0}{\gamma - 1} \tau ^\gamma$ that satisfies (5.2). So it suffices to prove (5.4) for applying Theorem 5.2. In order to verify condition (5.4), we also employ a corollary from [72].

### Corollary 5.3

([72, Corollary II.1]). Suppose that (5.1) and (5.2) are satisfied. If, for some $\beta \in (0, n)$,

5.5 $$\begin{aligned} \Psi (t \xi ) \ge t^{-\beta } \Psi (\xi ) \qquad \text { for all } t \ge 1 \text { and } \xi \in \mathbb {R}^n \text { a.e.}, \end{aligned}$$

then (5.4) is satisfied if and only if $F_M < 0$.

Denoting by

$$\begin{aligned} g_M = \inf _{\varrho \in X_M} \mathcal {G}(\varrho ), \end{aligned}$$

the result in [17, Theorem 5] shows that $g_M<0, \text { for all } M>0.$ This is obtained via a simple scaling argument on a suitably normalized characteristic of a ball by using the homogeneities of the entropy and the interaction parts of functional $\mathcal {G}$ to express this as a function of the radius of the ball with competing homogeneities. This shows (5.4) for the free energy functional $\mathcal {G}$.

Clearly, $\Phi _\alpha$ satisfies (5.5). In fact, with an equality for $\beta = \alpha$ and for all $\xi \in \mathbb {R}^n\setminus \{\textbf{0}\}$ that is for $\xi \in \mathbb {R}^n$ *a.e.*, since $g_M < 0$, Corollary 5.3 implies that there exists a minimizer $\tilde{\rho } \in X_M$ to $\mathcal {G}$ over $X_M$. The rest of the properties of the global minimizers of $\mathcal {G}$ over $X_M$ are referred to [17]. This finishes the proof of Theorem 5.1.

We now prove the existence of a radial minimizer of functional (5.3) over $\mathcal {A}_M$ under conditions (5.1) and (5.2).

### Lemma 5.4

Suppose that (5.1)–(5.2) are satisfied, with Ψ radially symmetric and radially decreasing. Then there exists a radial minimizer $\tilde{\rho } \in \mathcal {A}_M$ of $\mathcal {F}$ over $\mathcal {A}_M$. Moreover, any minimizer of $\mathcal {F}$ over $\mathcal {A}_M$ is the translation of a radially symmetric and decreasing minimizer.

### Proof

Suppose that $\tilde{\rho }$ is a minimizer of $\mathcal {F}$ over $\mathcal {A}_M$. Then

$$ \int _{\mathbb {R}^n} (\tilde{\rho }^* e(\tilde{\rho }^*))(\boldsymbol{x})\,{\, \mathrm d}\boldsymbol{x} = \int _{\mathbb {R}^n} (\tilde{\rho } e(\tilde{\rho }))(\boldsymbol{x})\,{\, \mathrm d}\boldsymbol{x}, $$

where $\tilde{\rho }^*$ is the symmetric decreasing rearrangement of $\tilde{\rho }$ defined in (A.2) in Appendix A.

If Ψ is spherically symmetric and decreasing in *r* so that $\Psi ^* = \Psi$, by Lemma A.4, we have

$$\begin{aligned} - \int _{\mathbb {R}^n} \tilde{\rho }^*(\boldsymbol{x}) (\Psi * \tilde{\rho }^*)(\boldsymbol{x}) {\, \mathrm d}\boldsymbol{x} \le - \int _{\mathbb {R}^n} \tilde{\rho }(\boldsymbol{x}) (\Psi * \tilde{\rho })(\boldsymbol{x}) {\, \mathrm d}\boldsymbol{x}, \end{aligned}$$

with the equality only if $\tilde{\rho } = \rho ^*(\cdot - y)$ for some $y \in \mathbb {R}^n$. Thus, $\mathcal {F}(\rho ^*) \le \mathcal {F}(\tilde{\rho })$. Since $\tilde{\rho }$ is a minimizer of $\mathcal {F}$ over $\mathcal {A}_M$, the equality in fact holds, $\tilde{\rho } {:=}\rho ^*$ is a radial minimizer, and $\tilde{\rho }$ is the translation of a radial minimizer. □

We finally discuss further properties of the global minimizers and their uniqueness. In Carrillo-Hoffmann-Mainini-Volzone [17], they proved that, for the Riesz potential ($\alpha \in [0,n)$) and in the case that n≥1 and $\frac{n + \alpha }{n}< \gamma < \gamma ^*$ with

5.6 $$\begin{aligned} \gamma ^* {:=}{\left\{ \begin{array}{ll} \frac{\alpha - (n - 2)}{\alpha - (n-1)} \quad &  \text {for } \alpha \in (n-1,n),\\ \infty &  \text {for } \alpha \in [0,n-1], \end{array}\right. } \end{aligned}$$

all the global minimizers of $\mathcal {G}$ in $X_M$ are steady states of (1.1). Moreover, if $\frac{n + \alpha }{n} < \gamma \le 2$, then any global minimizers $\varrho \in X_M$ are in the space $\varrho \in \mathcal {W}^{1,\infty }(\mathbb {R}^n)$. Furthermore, for n=1, $\alpha \in (0,1)$, and $\gamma > \frac{n + \alpha }{n}$, the global minimizers of $\mathcal {G}$ in $X_M$ and the steady states of (1.1) in $X_M$ are unique (up to translation). Similar results hold for α=0 as proven in [14], so this case is included in (5.6).

Calvez-Carrillo-Hoffmann [12] proved that, for n≥2, $\alpha \in [n-2,n)$, and $\gamma > \frac{n + \alpha }{n}$, the steady states of (1.1) in $X_M$ are unique (up to translation). Thus, for n≥2, $\alpha \in [n-2,n)$, and $\frac{n + \alpha }{n}< \gamma < \gamma ^*$, there is a unique minimizer of $\mathcal {G}$ over $X_M$ (up to translation) that coincides with the unique steady state of (1.1) in $X_M$ (up to translation). It was also proved that, for the critical case that $\alpha \in [n-2,n)$ and $\gamma = \frac{n + \alpha }{n}$, there exists a unique (up to translation) minimizer of $\mathcal {G}$ over $X_{M_\textrm{c}}$ and a steady state of (1.1) in $X_{M_\textrm{c}}$ for some critical mass $M_\textrm{c}$.

Chan-González-Huang-Mainini-Volzone [22] proved that, for n≥1, $\alpha \in (0,1)$ for n=1, and $\alpha \in [n-2,n)$ for n≥2, and $\frac{2n}{2n - \alpha }< \gamma < \frac{n + \alpha }{n}$, there exist a unique radial steady state $\tilde{\rho } \in X_M \cap L^\infty (\mathbb {R}^n)$ to (1.1) in $X_M$ in the sense that

5.7 $$\begin{aligned} e(\tilde{\rho })(\boldsymbol{x}) =\big (-\frac{1}{\gamma } (\Phi _\alpha * \tilde{\rho })(\boldsymbol{x}) - \mathcal {K}\big )_+ \end{aligned}$$

is satisfied for *a.e.* $\boldsymbol{x} \in \mathbb {R}^n$ for some $\mathcal {K} \ge 0$. Here, (5.7) is derived by dividing the steady form of (1.1), in the attractive case (κ=1), by the density to obtain

$$ \nabla e(\tilde{\rho })(\boldsymbol{x}) = - \frac{1}{\gamma } \nabla \mathcal {W}_\alpha (\boldsymbol{x}). $$

Then, in the case that the density is regular enough, there must exist a constant $\mathcal {K}$ such that

$$ e(\tilde{\rho })(\boldsymbol{x}) = - \frac{1}{\gamma } (\Phi _\alpha * \tilde{\rho })(\boldsymbol{x}) - \mathcal {K} = \big (- \frac{1}{\gamma } (\Phi _\alpha * \tilde{\rho })(\boldsymbol{x}) - \mathcal {K} \big )_+. $$

Delgadino-Yan-Yao [40] considered non-local potentials of the form W∗ρ, where $W: \mathbb {R}^n \setminus \{\textbf{0}\} \rightarrow \mathbb {R}$ under the following assumptions:

1. $$W \in C^\infty (\mathbb {R}^n \setminus \{\textbf{0}\})$$ is radially symmetric, *W* is an attractive potential, and $W^{\prime }\left(r\right)>0$ for r>0, where *r* is the radial variable.
2. *W* is no more singular than the locally integrable potential $\Phi _\alpha$ at the origin, for some α<n. That is, there exists some C>0 such that $W'(r) \le C r^{-(\alpha + 1)}$ for r∈(0,1).
3. There exists C>0 such that $W^{\prime }\left(r\right)\leq C$ for all r≥1.
4. Either *W*(*r*) is bounded for r≥1 or there exists some C>0 such that, for all a,b≥0,
  $$ W_+(a+b) \le C(1 + W(1+a) + W(1+b)), $$
  where $$W_+ {:=}\max \{W,0\}$$.

Under these conditions (W1)–(W4) and $\gamma \ge 2$, there is at most one steady state in $X_M \cap L^\infty (\mathbb {R}^n)$ up to translation. Note that the potential function $\Phi _\alpha$, for $\alpha \in [-1,n)$, satisfies conditions (W1)–(W4). Moreover, in [18], they proved that, under the same conditions, the steady states with $W(1+|\boldsymbol{x}|)\tilde{\rho } \in L^1(\mathbb {R}^n)$ are radially decreasing.

Thus, combining [17, Theorem 2], [12, Theorem 3], [22, Proposition 5.16], [40, Theorem 1.1], and [18, Theorem 2.2], we obtain the following theorem.

### Theorem 5.5

(Existence and Uniqueness of Steady States and Global Minimizers).

(i) Suppose that n≥1, M>0, $\gamma > \frac{n + \alpha }{n}$, and
  - $$\,\alpha \in (0,1)$$ for n=1,
  - $$\,\alpha \in [n-2,n)$$ for n≥2.
  Then the steady states of (1.1) in $X_M$, as defined in Definition 2.4, are unique (up to translation) and radially decreasing. Moreover, if $\frac{n + \alpha }{n}< \gamma < \gamma ^*$, as defined in (5.6), then the global minimizers of $\mathcal {G}$ in $X_M$ are steady states and radially decreasing, which implies the uniqueness of the global minimizers in this range.
(ii) Suppose that n≥2, $\alpha \in [n-2,n)$, and $\gamma = \frac{n + \alpha }{n}$. Then there exists a unique minimizer of $\mathcal {G}$ that is a steady state of (1.1) in $X_{M_\textrm{c}}$, which is radially decreasing.
(iii) Suppose that n≥1, M>0, $\frac{2n}{2n - \alpha }< \gamma < \frac{n + \alpha }{n}$, and
  - $$\,\alpha \in (0,1)$$ for n=1,
  - $$\,\alpha \in [n-2,n)$$ for n≥2.
  Then there exists a unique radially decreasing steady state $\tilde{\rho } \in X_M \cap L^\infty (\mathbb {R}^n)$ to (1.1) in $X_M$ of (5.7).
(iv) Suppose that n≥1, M>0, $\gamma \ge 2$, and $\alpha \in [-1,n-2)$, then there exists a unique radially decreasing steady state $\tilde{\rho } \in X_M \cap L^\infty (\mathbb {R}^n)$ to (1.1) in $X_M$ of (5.7).

In order to obtain the stability of the unique stationary solution of CEREs in the previous theorem, we need to introduce some new quantities measuring the distance between a general solution and a stationary solution. Let $\tilde{\rho }$ be a minimizer of $\mathcal {G}$ on $X_M$, and let λ be the constant in Theorem 5.1. For $\varrho \in X_M,$ we define the distance:

$$\begin{aligned} d(\varrho ,\tilde{\rho })&{:=}\int _{\mathbb {R}^n}\big ((\varrho e(\varrho )-\tilde{\rho }e(\tilde{\rho }))+(\Phi _\alpha *\tilde{\rho })(\varrho -\tilde{\rho })\big )\,{\, \mathrm d}\boldsymbol{x}\\&=\int _{\mathbb {R}^n}\big ((\varrho e(\varrho )-\tilde{\rho }e(\tilde{\rho })) +(\Phi _\alpha *\tilde{\rho }-\lambda )(\varrho -\tilde{\rho })\big )\,{\, \mathrm d}\boldsymbol{x}\\&\ge \int _{\mathbb {R}^n}\big ((\varrho e(\varrho )-\tilde{\rho }e(\tilde{\rho })) -(\tilde{\rho }e(\tilde{\rho }))_{\tilde{\rho }}(\varrho -\tilde{\rho })\big )\,{\, \mathrm d}\boldsymbol{x}, \end{aligned}$$

where we have used the fact that $\int _{\mathbb {R}^n} \varrho (\boldsymbol{x})\,{\, \mathrm d}\boldsymbol{x} =\int _{\mathbb {R}^n} \tilde{\rho }(\boldsymbol{x})\,{\, \mathrm d}\boldsymbol{x}=M$ for $\varrho ,\tilde{\rho }\in X_M$ and Theorem 5.1. Notice that, by convexity of $\varrho e(\varrho )$, then $d(\varrho ,\tilde{\rho })\ge 0$ for any $\varrho \in X_M$.

Let us now connect this to the global finite-energy weak solutions of CEREs obtained in the previous sections. Denote $(\rho , \mathcal {M})(t)=(\rho , \mathcal {M})(t,\cdot )$ as given in the statement of Theorem 2.7. The energy $\mathcal {E}$ of the solution to CEREs is given by

$$\begin{aligned} \mathcal {E}(\rho (t),\mathcal {M}(t)) = \int _{\mathbb {R}^n}\Big ((\rho e(\rho ))(t,\boldsymbol{x}) + \frac{1}{2} \Big |\frac{\mathcal {M}}{\sqrt{\rho }}\Big |^2(t,\boldsymbol{x}) + \frac{1}{2}\rho (t,\boldsymbol{x})(\Phi _\alpha *\rho )(t,\boldsymbol{x})\Big )\,{\, \mathrm d}\boldsymbol{x}. \end{aligned}$$

Then, for a given solution $(\rho (t),\mathcal {M}(t))$ of CEREs with mass *M*, we have

5.8 $$\begin{aligned} \mathcal {E}(\rho (t),\mathcal {M}(t)) - \mathcal {E}(\tilde{\rho },\textbf{0}) = \mathcal {G}(\rho (t)) - \mathcal {G}(\tilde{\rho }) + \frac{1}{2}\int _{\mathbb {R}^n} \Big |\frac{\mathcal {M}}{\sqrt{\rho }}\Big |^2(t,\boldsymbol{x})\,{\, \mathrm d}\boldsymbol{x}. \end{aligned}$$

For $\alpha \in (0,n)$, we have

5.9 $$\begin{aligned} \begin{aligned} \mathcal {G}(\rho (t)) - \mathcal {G}(\tilde{\rho })&= d(\rho (t),\tilde{\rho }) + \frac{1}{2} \int _{\mathbb {R}^n} (\rho (t) - \tilde{\rho })\, \Phi _\alpha * (\rho (t) - \tilde{\rho }) {\, \mathrm d}\boldsymbol{x}. \end{aligned} \end{aligned}$$

Combining (5.8) with (5.9), we obtain

5.10 $$\begin{aligned} \begin{aligned} \mathcal {E}(\rho (t),\mathcal {M}(t)) - \mathcal {E}(\tilde{\rho },\boldsymbol{0})&= \mathcal {E}(\rho (t),\mathcal {M}(t)) - \mathcal {G}(\tilde{\rho }) \\&= d(\rho (t),\tilde{\rho }) + \frac{1}{2} \int _{\mathbb {R}^n} (\rho (t) - \tilde{\rho })\, \Phi _\alpha * (\rho (t) - \tilde{\rho }) {\, \mathrm d}\boldsymbol{x} \\&\quad + \frac{1}{2}\int _{\mathbb {R}^n} \Big |\frac{\mathcal {M}}{\sqrt{\rho }}\Big |^2(t,\boldsymbol{x})\,{\, \mathrm d}\boldsymbol{x}. \end{aligned} \end{aligned}$$

Notice that we can estimate the second term in the last equality, which can be obtained by the Hölder inequality and the HLS inequality from Lemma A.2, as such

$$ \Big |\int _{\mathbb {R}^n} (\rho (t) - \tilde{\rho }) \,\Phi _\alpha * (\rho (t) - \tilde{\rho }) {\, \mathrm d}\boldsymbol{x}\Big | \le C_{n,\alpha } \Vert \rho (t) - \tilde{\rho } \Vert _{L^\frac{2n}{2n - \alpha }}^2. $$

### Remark 5.6

Carrillo-Castorina-Volzone [14] proved that, for n=2, α=0, and γ>1, there exists a unique global minimizer of $\mathcal {G}$ in $X_M$, which is the unique radially decreasing, compactly supported, and smooth in its support steady state of (1.1) in the sense of distributions; however, in [14], only the weak compactness of the minimizing sequence was obtained. In this paper, we require the strong compactness of the minimizing sequence, allowing us to control the relative potential energy. For the Riesz potential (*i.e.,* $\alpha \in (0,n)$), we are able to control the relative potential energy by the relative $L^\frac{2n}{2n - \alpha }$–norm of the densities. However, in the logarithmic potential case, it is no longer apparent to find the natural controlling relative entropy or how we might obtain such a bound, so that we cannot control the relative potential energy and use (5.13) and (5.10) to obtain (5.14), since

$$ \frac{1}{2} \int _{\mathbb {R}^n} (\rho (t) - \tilde{\rho })\, \Phi _0* (\rho (t) - \tilde{\rho }) {\, \mathrm d}\boldsymbol{x} $$

does not have a sign. Thus, we cannot use the following argument by contradiction in this case.

### Proof of Theorem 2.7

We follow a similar approach as in Rein [80]; however, we take advantage of having the uniqueness of global minimizers modulo translations. Suppose the statement in Theorem 2.7 is not true, then there exist ε>0 and a sequence of global weak solutions $(\rho ^j,\mathcal {M}^j)$ of CEREs associated to the initial data $\rho ^j_0$ and a sequence of times $t_j > 0$ such that

5.11 $$\begin{aligned} d(\rho ^j_0,\tilde{\rho })+ \Vert \rho ^j_0 - \tilde{\rho } \Vert _{L^\frac{2n}{2n - \alpha }}^2 +\frac{1}{2}\int _{\mathbb {R}^n}\Big |\frac{\mathcal {M}^j_0}{\sqrt{\rho ^j_0}}\Big |^2\,{\, \mathrm d}\boldsymbol{x}<\frac{1}{j}, \end{aligned}$$

and

5.12 $$\begin{aligned} d(\rho ^j(t_j),T^{\boldsymbol{y}}\tilde{\rho }) + \Vert \rho ^j(t_j) - T^{\boldsymbol{y}} \tilde{\rho } \Vert _{L^\frac{2n}{2n - \alpha }}^2 +\frac{1}{2}\int _{\mathbb {R}^n}\Big |\frac{\mathcal {M}^j}{\sqrt{\rho ^j}}\Big |^2(t_j) \,{\, \mathrm d}\boldsymbol{x} \ge \varepsilon \end{aligned}$$

for all $\boldsymbol{y} \in \mathbb {R}^n$. Notice that the sequence of times $t_j$ can be chosen outside the zero measure set, where the terms in (5.12) are well defined, according to Definition 2.1.

Then, by (5.11) and (5.9), we have

$$\begin{aligned} \lim _{j \rightarrow \infty } \mathcal {E}(\rho ^j_0,\mathcal {M}^j_0) = \mathcal {E}(\tilde{\rho },\boldsymbol{0}) = \mathcal {G}(\tilde{\rho }). \end{aligned}$$

Since the energy $\mathcal {E}$ for the weak solutions is non-increasing from the initial energy by definition, we have

$$\begin{aligned} \limsup _{j \rightarrow \infty } \mathcal {G}(\rho ^j(t_j)) \le \limsup _{j \rightarrow \infty } \mathcal {E}(\rho ^j(t_j),\mathcal {M}^j(t_j)) \le \lim _{j \rightarrow \infty } \mathcal {E}(\rho ^j_0,\mathcal {M}^j_0) = \mathcal {G}(\tilde{\rho }). \end{aligned}$$

Thus, $\rho ^j(t_j) \in X_M$ is a minimizing sequence of $\mathcal {G}$ in $X_M$ and, by Theorem 5.2, there exists $\boldsymbol{y}_j \in \mathbb {R}^n$ such that $T^{\boldsymbol{y}_j} \rho ^j(t_j) \rightarrow \tilde{\rho }$ in $L^1(\mathbb {R}^n) \cap L^\frac{n + \alpha }{n}(\mathbb {R}^n)$, due to the uniqueness of $\tilde{\rho }$ up to translation. Then

$$\begin{aligned} \Vert \rho ^j(t_j) - T^{-\boldsymbol{y}_j} \tilde{\rho } \Vert _{L^\frac{2n}{2n - \alpha }} = \Vert T^{\boldsymbol{y}_j} \rho ^j(t_j) - \tilde{\rho } \Vert _{L^\frac{2n}{2n - \alpha }} \longrightarrow 0 \qquad \text {as } j \rightarrow \infty , \end{aligned}$$

since $\frac{2n}{2n - \alpha } \in (1, \frac{n + \alpha }{n})$. We see that, for any *i*,

5.13 $$\begin{aligned} \lim _{j \rightarrow \infty } \mathcal {E}(\rho ^j(t_j),\mathcal {M}^j(t_j)) = \mathcal {G}(\tilde{\rho }) = \mathcal {G}(T^{-\boldsymbol{y}_i}\tilde{\rho }). \end{aligned}$$

Therefore, by (5.10),

5.14 $$\begin{aligned} d(\rho ^j(t_j),T^{-\boldsymbol{y}_j}\tilde{\rho }) + \frac{1}{2}\int _{\mathbb {R}^n} \Big |\frac{\mathcal {M}^j}{\sqrt{\rho ^j}}\Big |^2(t_j)\,{\, \mathrm d}\boldsymbol{x} \longrightarrow 0 \qquad \text {as } j \rightarrow \infty . \end{aligned}$$

This is a contradiction to (5.12), which completes the proof. □

### Remark 5.7

Suppose that $\tilde{\rho }$ is a unique minimizer of $\mathcal {G}$ in $X_M$. Without loss of generality, suppose that $0< \delta = \delta (\varepsilon ) < \varepsilon$ satisfies Theorem 2.7. DefineThen, for $\boldsymbol{y} \in A_\varepsilon$, there exists a weak solution $(\rho ,\mathcal {M})$ of CEREs such that (2.8)–(2.9) are satisfied for ε and $\delta (\varepsilon )$:

$$ \Vert \tilde{\rho } - T^{\boldsymbol{y}} \tilde{\rho } \Vert _{L^\frac{2n}{2n - \alpha }}^2 \le \Vert \tilde{\rho } - \rho _0 \Vert _{L^\frac{2n}{2n - \alpha }}^2 + \Vert \rho _0 - T^{\boldsymbol{y}}\tilde{\rho } \Vert _{L^\frac{2n}{2n - \alpha }}^2 \le \varepsilon + \delta (\varepsilon ). $$

Thus, as $\varepsilon \rightarrow 0$, we have

$$ \sup _{\boldsymbol{y} \in A_\varepsilon } \Vert \tilde{\rho } - T^{\boldsymbol{y}} \tilde{\rho } \Vert _{L^\frac{2n}{2n - \alpha }}^2 \longrightarrow 0. $$

Since $\tilde{\rho } \in L^\frac{2n}{2n - \alpha }(\mathbb {R}^n)$, we must have

$$ \sup _{\boldsymbol{y} \in A_\varepsilon } |\boldsymbol{y}| \longrightarrow 0\qquad \text {as } \varepsilon \rightarrow 0. $$

That is, the closer we start to $\tilde{\rho }$, the closer we remain to $\tilde{\rho }$, not just a translation of $\tilde{\rho }$.

### Remark 5.8

In case we no longer have the uniqueness of the minimizers, as in [80], we can define the set of minimizers of $\mathcal {G}$ in $X_M$ by

$$ \mathcal {M}_M {:=}\big \{ \tilde{\rho } \in X_M \,: \, \mathcal {G}(\tilde{\rho }) = g_M \big \}, $$

and prove the following corollary:

### Corollary 5.9

(Nonlinear Stability of CEREs). Suppose that $\alpha \in (0,n - 1)$ with $\gamma > \frac{n + \alpha }{n}$. Let $(\rho ,\mathcal {M})(t, \boldsymbol{x})$ be a global weak solution of the attractive CEREs (κ=1) in the sense of Definition 2.1 satisfying

$$ \int _{\mathbb {R}^n} \rho _0(\boldsymbol{x})\,{\, \mathrm d}\boldsymbol{x} =\int _{\mathbb {R}^n} \tilde{\rho }(\boldsymbol{x})\,{\, \mathrm d}\boldsymbol{x}=M. $$

Then, for any ε>0, there exists $\delta = \delta (\varepsilon )>0$ such that, if

$$\begin{aligned} \inf _{\tilde{\rho } \in \mathcal {M}_M} \bigg \{ d(\rho _0,\tilde{\rho })+ \Vert \rho _0 - \tilde{\rho } \Vert _{L^\frac{2n}{2n - \alpha }}^2 +\frac{1}{2}\int _{\mathbb {R}^n} \Big |\frac{\mathcal {M}_0}{\sqrt{\rho _0}}\Big |^2\,{\, \mathrm d}\boldsymbol{x} \bigg \} <\delta , \end{aligned}$$

then

$$\begin{aligned} \inf _{\tilde{\rho } \in \mathcal {M}_M} \bigg \{ d(\rho (t),\tilde{\rho })+ \Vert \rho (t) - \tilde{\rho } \Vert _{L^\frac{2n}{2n - \alpha }}^2 +\frac{1}{2}\int _{\mathbb {R}^n} \Big |\frac{\mathcal {M}}{\sqrt{\rho }}\Big |^2(t)\,{\, \mathrm d}\boldsymbol{x} \bigg \} <\varepsilon \qquad \text {for a.e. } t>0. \end{aligned}$$

Furthermore, let $\tilde{\rho } \in \mathcal {M}_M$ be an isolated minimizer (up to translation) of functional $\mathcal {G}$ in $X_M$, that is,

$$ \delta _0 {:=}\inf \Big \{ \Vert \rho - \tilde{\rho } \Vert _{L^\frac{2n}{2n - \alpha }}^2 \,:\, \rho \in \mathcal {M}_M {\setminus } \{ T^{\boldsymbol{y}}\tilde{\rho } \,: \, \boldsymbol{y} \in \mathbb {R}^n \} \Big \}>0, $$

and let

5.15 $$\begin{aligned} (t,\boldsymbol{y}) \longmapsto d(\rho (t),T^{\boldsymbol{y}}\tilde{\rho }) + \Vert \rho (t) - T^{\boldsymbol{y}}\tilde{\rho } \Vert _{L^\frac{2n}{2n - \alpha }}^2 +\frac{1}{2}\int _{\mathbb {R}^n} \Big |\frac{\mathcal {M}}{\sqrt{\rho }}\Big |^2(t)\,{\, \mathrm d}\boldsymbol{x} \end{aligned}$$

be continuous. Then, for any $0< \varepsilon < \frac{\delta _0}{4}$, there exists $0<\delta = \delta (\varepsilon )<\varepsilon$ such that, if

$$\begin{aligned} d(\rho _0,\tilde{\rho })+ \Vert \rho _0 - \tilde{\rho } \Vert _{L^\frac{2n}{2n - \alpha }}^2 +\frac{1}{2}\int _{\mathbb {R}^n} \Big |\frac{\mathcal {M}_0}{\sqrt{\rho _0}}\Big |^2\,{\, \mathrm d}\boldsymbol{x}<\delta , \end{aligned}$$

there exists a translation $\boldsymbol{y} \in \mathbb {R}^n$ such that

$$\begin{aligned} d(\rho (t),T^{\boldsymbol{y}}\tilde{\rho }) + \Vert \rho (t) - T^{\boldsymbol{y}}\tilde{\rho } \Vert _{L^\frac{2n}{2n - \alpha }}^2 +\frac{1}{2}\int _{\mathbb {R}^n} \Big |\frac{\mathcal {M}}{\sqrt{\rho }}\Big |^2(t)\,{\, \mathrm d}\boldsymbol{x}<\varepsilon \qquad \text {for all } t>0. \end{aligned}$$

Notice that this result needs the additional continuity assumption of (5.15); see [80] for details.

## Data Availability

Data sharing is not applicable to this article as no datasets were generated or analyzed during the current study.

## Convolution Inequalities

We now present several convolution inequalities that have been used in the main text of this paper.

### Theorem A.1

([43, Theorem II.11.2]). Suppose that $\Omega \subset \mathbb {R}^n$ is bounded, $\alpha \in (0,n)$, and $f \in L^q(\Omega )$ for $q \in (1,\infty )$. If $\alpha < n(1 - \frac{1}{q})$, then $|\cdot |^{-\alpha } * (1\!\!1_\Omega f) \in C^{0,\mu }(\overline{\Omega })$ for $\mu := \min \big \{1,n(1-\frac{1}{q})-\alpha \big \}$ and there exists some constant $C = C(\Omega ,n,q,\alpha )$ such that

$$\begin{aligned} \big \Vert |\cdot |^{-\alpha } * (1\!\!1_\Omega f)\big \Vert _{C^{0,\mu }(\overline{\Omega })} \le C \Vert f\Vert _{L^q(\Omega )}. \end{aligned}$$

### Lemma A.2

(Hardy-Littlewood-Sobolev (HLS) Inequality). Let $\alpha \in (0,n)$ and 1<p,r<∞ such that

$$ \frac{1}{p} + \frac{\alpha }{n} = 1 + \frac{1}{r}. $$

Then there exists a constant $C_{n,\alpha }>0$ such that, for all $f \in L^p(\mathbb {R}^n)$,

$$ \big \Vert |\cdot |^{-\alpha } * f \big \Vert _{L^r(\mathbb {R}^n)} \le C_{n,\alpha }\Vert f\Vert _{L^p(\mathbb {R}^n)}. $$

When $r = \frac{2n}{\alpha }$ and $p=\frac{2n}{2n-\alpha }$, the constant $C_{n,\alpha }$ is given by

$$ C_{n,\alpha }:= \pi ^\frac{\alpha }{2} \frac{\Gamma (\frac{n - \alpha }{2})}{\Gamma ( n - \frac{\alpha }{2})} \bigg ( \frac{\Gamma (\frac{n}{2})}{\Gamma (n)} \bigg )^{\frac{\alpha - n}{n}}. $$

### Lemma A.3

(Variation of HLS Inequality [11, Theorem 3.1]). Suppose that $\alpha \in (0,n)$ and $p \ge \frac{2n}{2n - \alpha }$. Then there exists $C_* = C_*(\alpha ,p,n) > 0$ such that, for any $f \in L^1(\mathbb {R}^n) \cap L^p(\mathbb {R}^n)$,

A.1 $$\begin{aligned} \bigg | \int _{\mathbb {R}^n} \int _{\mathbb {R}^n} f(\boldsymbol{x}) |\boldsymbol{x} - \boldsymbol{y}|^{-\alpha } f(\boldsymbol{y}) {\, \mathrm d}\boldsymbol{x} {\, \mathrm d}\boldsymbol{y} \bigg | \le C_* \Vert f \Vert _{L^1}^\frac{p (2n - \alpha ) - 2n}{n(p - 1)} \Vert f \Vert _{L^p}^\frac{\alpha p}{n(p - 1)}. \end{aligned}$$

In particular, when $p = \frac{n + \alpha }{n} > \frac{2n}{2n - \alpha }$, for any $f \in L^1(\mathbb {R}^n) \cap L^{\frac{n + \alpha }{n}}(\mathbb {R}^n)$,

$$ \bigg | \int _{\mathbb {R}^n} \int _{\mathbb {R}^n} f(\boldsymbol{x}) |\boldsymbol{x} - \boldsymbol{y}|^{-\alpha } f(\boldsymbol{y}) {\, \mathrm d}\boldsymbol{x} {\, \mathrm d}\boldsymbol{y} \bigg | \le C_* \Vert f \Vert _{L^1}^\frac{n - \alpha }{n} \Vert f \Vert _{L^{\frac{n + \alpha }{n}}}^\frac{n + \alpha }{n}. $$

Furthermore, $C_*$ is the sharp constant that satisfies (A.1) and is strictly bounded above by the standard HLS constant $C_{n,\alpha }$, i.e., $C_* < C_{n,\alpha }$.

### Lemma A.4

(Riesz’s Rearrangement Inequality [68]). Let $f, g, h: \mathbb {R}^n \rightarrow \mathbb {R}_+$ be measurable functions. Define

$$ I(f,g,h) {:=}\int _{\mathbb {R}^n} \int _{\mathbb {R}^n} f(\boldsymbol{x}) g(\boldsymbol{x} - \boldsymbol{y}) h(\boldsymbol{y}) {\, \mathrm d}\boldsymbol{x} {\, \mathrm d}\boldsymbol{y}. $$

For a given measurable function $\phi : \mathbb {R}^n \rightarrow \mathbb {R}_+$, define the symmetric decreasing rearrangement $\phi ^*$ of ϕ by

A.2 $$\begin{aligned} \phi ^* (\boldsymbol{x}) {:=}\int ^\infty _0 1\!\!1_{\{\boldsymbol{y} \, : \, \phi (\boldsymbol{y}) > t\}^*} (\boldsymbol{x}) {\, \mathrm d}t \end{aligned}$$

with

$$ A^* = \{ \boldsymbol{x} \,: \, \frac{\omega _n}{n} |\boldsymbol{x}|^n \le |A|\} $$

for any Lebesgue measurable set $A \subset \mathbb {R}^n$. Then

A.3 $$\begin{aligned} I(f,g,h) \le I(f^*,g^*,h^*). \end{aligned}$$

If *g* is a strictly symmetric-decreasing function, then the equality holds in (A.3) if and only if there exists some $\boldsymbol{z} \in \mathbb {R}^n$ such that $f(\boldsymbol{x}) = f^*(\boldsymbol{x} - \boldsymbol{z})$ and $h(\boldsymbol{x}) = h^*(\boldsymbol{x} - \boldsymbol{z})$.

### Theorem A.5

(The Riesz Composition Formula [79, Theorem 3.1]). Suppose that 0<α<n, 0<β<n, and $0< \alpha + \beta < n$. Then

$$\begin{aligned} \int _{\mathbb {R}^n} |\boldsymbol{x} - \boldsymbol{z}|^{\alpha - n} |\boldsymbol{z} - \boldsymbol{y}|^{\beta - n} {\, \mathrm d}\boldsymbol{z} = \kappa _{\alpha ,\beta }\,|\boldsymbol{x} - \boldsymbol{y}|^{\alpha + \beta - n}, \end{aligned}$$

where

A.4 $$\begin{aligned} \kappa _{\alpha ,\beta } = \frac{\pi ^{\frac{n}{2}} \Gamma (\frac{\alpha }{2}) \Gamma (\frac{\beta }{2}) \Gamma (\frac{n-\alpha -\beta }{2})}{\Gamma (\frac{n-\alpha }{2}) \Gamma (\frac{n - \beta }{2}) \Gamma (\frac{\alpha + \beta }{2})}. \end{aligned}$$

## Construction of the Approximate Initial Data

Similar to [26], we approximate the initial density by a density function away from the vacuum, which means that our approximate solutions can also be away from the vacuum, giving us simpler calculations, as we consider the equations in terms of the velocity and the density, rather than the momentum and the density.

Consider a standard radially symmetric mollifier $J(\boldsymbol{x})$ and the associated mollifier:

$$ J_\delta (\boldsymbol{x}) {:=}\frac{1}{\delta ^n}J(\frac{\boldsymbol{x}}{\delta }). $$

We now define the approximation:

$$\begin{aligned} \tilde{\rho }^\varepsilon _0(\boldsymbol{x}) {:=}\bigg ( \int _{\mathbb {R}^n} \sqrt{\rho _0(\boldsymbol{x}- \boldsymbol{y})} J_{\sqrt{\varepsilon }}(\boldsymbol{y}) {\, \mathrm d}\boldsymbol{y} + \varepsilon e^{-|\boldsymbol{x}|^2} \bigg )^2. \end{aligned}$$

We have defined the approximation as such in order to have a nice formula for $\sqrt{\tilde{\rho }^\varepsilon _0}(\boldsymbol{x})$ with the first part smoothing the square root of the density and the second part avoiding any vacuum states that might occur in the initial density. Then, as in [26], we normalize to ensure that the mass of the approximating initial density remains constant as ε varies. We define the initial density approximation:

B.1 $$\begin{aligned} \rho ^\varepsilon _0(\boldsymbol{x}) {:=}\frac{M}{\int _{\mathbb {R}^n} \tilde{\rho }^\varepsilon _0(\boldsymbol{x}) {\, \mathrm d}\boldsymbol{x}} \tilde{\rho }^\varepsilon _0(\boldsymbol{x}). \end{aligned}$$

Then, similar to [26], we have the following lemma:

### Lemma B.1

Let $q \in \{1,\gamma \}$ for κ=1 and $q \in \{1,\gamma ,\frac{2n}{2n - \alpha }\}$ for κ=-1. Then

B.2 $$\begin{aligned} \begin{aligned}&\int _{\mathbb {R}^n}\rho _0^\varepsilon (\boldsymbol{x})\,{\, \mathrm d}\boldsymbol{x}=M, \quad \Vert \rho _0^\varepsilon \Vert _{L^q}\le C\big (\Vert \rho _0\Vert _{L^q}+1\big ) \qquad \,\,\text {for all } \varepsilon \in (0,1],\\&\lim _{\varepsilon \rightarrow 0} \big (\Vert \rho _0^\varepsilon - \rho _0\Vert _{L^q}+\Vert \sqrt{\rho _0^\varepsilon } - \sqrt{\rho _0}\Vert _{L^{2q}}\big )=0,\\&\varepsilon ^2\int _{\mathbb {R}^n}\Big |\nabla _{\boldsymbol{x}}\sqrt{\rho ^\varepsilon _{0} (\boldsymbol{x})}\Big |^2 {\, \mathrm d}\boldsymbol{x} \le C\varepsilon \big (\Vert \rho _0\Vert _{L^1}+1\big ) \longrightarrow 0\qquad \text {as } \varepsilon \rightarrow 0. \end{aligned} \end{aligned}$$

In the case that $\alpha \in (-1,0]$, $\Phi _\alpha * \rho ^\varepsilon _0$ is well-defined:

B.3 $$\begin{aligned}&\int _{\mathbb {R}^n} \rho ^\varepsilon _0(\boldsymbol{y}) |\Phi _\alpha (\boldsymbol{x} - \boldsymbol{y})| {\, \mathrm d}\boldsymbol{y}\nonumber \\&\le \frac{2M}{\int _{\mathbb {R}^n} \tilde{\rho }^\varepsilon _0(\boldsymbol{y}) {\, \mathrm d}\boldsymbol{y}} \int _{\mathbb {R}^n} \tilde{\rho }^\varepsilon _0(\boldsymbol{y}) |\Phi _\alpha (\boldsymbol{x} - \boldsymbol{y})| {\, \mathrm d}\boldsymbol{y} \nonumber \\&\le \frac{4M}{\int _{\mathbb {R}^n} \tilde{\rho }^\varepsilon _0(\boldsymbol{y}) {\, \mathrm d}\boldsymbol{y}} \int _{\mathbb {R}^n} \Big ( |\sqrt{\rho _0} * J_{\sqrt{\varepsilon }}|^2(\boldsymbol{y}) + \varepsilon ^2 e^{-2|\boldsymbol{y}|^2} \Big ) |\Phi _\alpha (\boldsymbol{x}-\boldsymbol{y})| {\, \mathrm d}\boldsymbol{y}\nonumber \\&= \frac{4M}{\int _{\mathbb {R}^n} \tilde{\rho }^\varepsilon _0(\boldsymbol{y}) {\, \mathrm d}\boldsymbol{y}} \int _{\mathbb {R}^n} \bigg ( \Big |\int _{\mathbb {R}^n} \sqrt{\rho _0}(\boldsymbol{y} - \boldsymbol{z}) J_{\sqrt{\varepsilon }}(\boldsymbol{z}) {\, \mathrm d}\boldsymbol{z} \Big |^2 + \varepsilon ^2 e^{-2|\boldsymbol{y}|^2} \bigg ) |\Phi _\alpha (\boldsymbol{x} - \boldsymbol{y})| {\, \mathrm d}\boldsymbol{y}\nonumber \\&\le \frac{4M}{\int _{\mathbb {R}^n} \tilde{\rho }^\varepsilon _0(\boldsymbol{y}) {\, \mathrm d}\boldsymbol{y}} \bigg ( \Big ( \int _{\mathbb {R}^n} J_{\sqrt{\varepsilon }} (\boldsymbol{z}) \sqrt{(|\Phi _\alpha | * \rho _0)(\boldsymbol{x} - \boldsymbol{z})} {\, \mathrm d}\boldsymbol{z} \Big )^2 + \int _{\mathbb {R}^n} \varepsilon ^2 e^{-2|\boldsymbol{y}|^2}\, |\Phi _\alpha (\boldsymbol{x} - \boldsymbol{y})| {\, \mathrm d}\boldsymbol{y} \bigg )\nonumber \\&< \infty \end{aligned}$$

where we have used the Minkowski inequality to obtain the penultimate inequality. We can also prove the following convergence result for the kernel:

### Lemma B.2

$\Phi _\alpha *\rho ^{\varepsilon }_0$ with $\alpha \in (0,n-1)$ satisfies the following:

$$\begin{aligned} \begin{aligned}&\Vert \Phi _\alpha *\rho _0^\varepsilon - \Phi _\alpha * \rho _0\Vert _{L^{\frac{2n}{\alpha }}} \le C \Vert \rho ^\varepsilon _0 - \rho _0\Vert _{L^\frac{2n}{2n - \alpha }} \longrightarrow 0 \qquad \text { as } \varepsilon \rightarrow 0, \\&\Vert \nabla (\Phi _\alpha *\rho ^\varepsilon _0 - \Phi _\alpha * \rho _0)\Vert _{L^\frac{2n}{\alpha + 2}} \le C \Vert \rho ^\varepsilon _0 - \rho _0\Vert _{L^\frac{2n}{2n - \alpha }} \longrightarrow 0 \qquad \text { as } \varepsilon \rightarrow 0. \end{aligned} \end{aligned}$$

For $$\alpha \in (-1,0]$$ and $$K \Subset \mathbb {R}^n$$,

B.4 $$\begin{aligned} \Vert \Phi _\alpha *\rho _0^\varepsilon - \Phi _\alpha * \rho _0\Vert _{L^\infty (K)} + \Vert \nabla (\Phi _\alpha *\rho ^\varepsilon _0 - \Phi _\alpha * \rho _0)\Vert _{L^\frac{2n}{\alpha +2}(K)} \longrightarrow 0 \qquad \text { as } \varepsilon \rightarrow 0. \end{aligned}$$

Moreover, there exists a constant C>0 such that, for ε>0 sufficiently small,

B.5 $$\begin{aligned} \int _{\mathbb {R}^n} \rho _0^\varepsilon (\boldsymbol{x}) k_\alpha (1 + |\boldsymbol{x}|^2) {\, \mathrm d}\boldsymbol{x} \le C. \end{aligned}$$

### Proof

The first part is an application of the HLS inequality from Lemma A.2. For simplicity, we may assume that $K = B_D$ for some D>0. Then, for ${\hat{\sigma }} > 0$,

$$\begin{aligned} \begin{aligned}&\big \Vert \Phi _\alpha *\rho _0^\varepsilon - \Phi _\alpha * \rho _0 \big \Vert _{L^\infty (K)} \\&\quad \le \big \Vert (1\!\!1_{B_{\hat{\sigma }}}|\Phi _\alpha |)*(\rho _0^\varepsilon - \rho _0) \big \Vert _{L^\infty (K)} + \big \Vert (1\!\!1_{B_{\hat{\sigma }}^c}|\Phi _\alpha |)*(\rho _0^\varepsilon - \rho _0) \big \Vert _{L^\infty (K)} \\&\quad =: I_1 + I_2. \end{aligned} \end{aligned}$$

Since $1\!\!1_{B_{\hat{\sigma }}}\Phi _\alpha \in L^\frac{\gamma }{\gamma - 1}$, then, for $I_1$, by the Hölder inequality,

B.6 $$\begin{aligned} I_1 \le \Vert \rho _0^\varepsilon - \rho _0\Vert _{L^\gamma } \Vert 1\!\!1_{B_{\hat{\sigma }}}\Phi _\alpha \Vert _{L^\frac{\gamma }{\gamma - 1}} \longrightarrow 0 \qquad \text { as } \varepsilon \rightarrow 0. \end{aligned}$$

We now want to show that $\Vert (1\!\!1_{B_{\hat{\sigma }}^c} |\Phi _\alpha |)*\rho _0 \Vert _{L^\infty (B_{D + 1})} \rightarrow 0$ as ${\hat{\sigma }} \rightarrow \infty$. We take ${\hat{\sigma }}$ large enough so that, for all $\boldsymbol{x} \in B_{D + 1}$ and $\boldsymbol{y} \in B_{{\hat{\sigma }} - D - 1}^c$, $1 \le |\boldsymbol{x}-\boldsymbol{y}| \le 2|\boldsymbol{y}|$. Then, for any $\boldsymbol{x} \in B_{D + 1}$,

$$\begin{aligned}&\big |(1\!\!1_{B_{\hat{\sigma }}^c} |\Phi _\alpha |)*\rho _0\big |(\boldsymbol{x}) \\&\le 2 \int _{\mathbb {R}^n} \rho _0(\boldsymbol{y}) 1\!\!1_{B_{\hat{\sigma }}^c}(\boldsymbol{x} - \boldsymbol{y}) |k_\alpha (|\boldsymbol{x} - \boldsymbol{y}|^2)| {\, \mathrm d}\boldsymbol{y} \\&\le \int _{B_{{\hat{\sigma }} - D - 1}^c} \rho _0(\boldsymbol{y}) |k_\alpha (|\boldsymbol{x} - \boldsymbol{y}|^2)| {\, \mathrm d}\boldsymbol{y} \\&\le C \int _{B_{{\hat{\sigma }} - D - 1}^c} \rho _0(\boldsymbol{y}) \big (1 + |k_\alpha (1 + |\boldsymbol{y}|^2)| \big ) {\, \mathrm d}\boldsymbol{y}. \end{aligned}$$

This implies

By the Lebesgue dominated convergence theorem, we obtain

$$ \int _{\mathbb {R}^n} \tilde{\rho }^\varepsilon _0(\boldsymbol{x}) {\, \mathrm d}\boldsymbol{x} \rightarrow M \qquad \text { as}\quad \varepsilon \rightarrow 0. $$

Then, for ε small enough, $\int _{\mathbb {R}^n} \tilde{\rho }^\varepsilon _0(\boldsymbol{x}) {\, \mathrm d}\boldsymbol{x} \ge \frac{M}{2}$. Moreover, for $\varepsilon \in (0,1)$ small enough, if $\boldsymbol{x} \in K$, by a similar calculation to (B.3), we have

$$\begin{aligned} \begin{aligned}&\big |(1\!\!1_{B_{\hat{\sigma }}^c}|\Phi _\alpha |)*\rho _0^\varepsilon \big |(\boldsymbol{x}) \\&\quad \le \frac{2M}{\int _{\mathbb {R}^n} \tilde{\rho }^\varepsilon _0(\boldsymbol{y}) {\, \mathrm d}\boldsymbol{y}} \bigg ( \Big ( \int _{\mathbb {R}^n} J_{\sqrt{\varepsilon }} (\boldsymbol{z}) \sqrt{((1\!\!1_{B_{\hat{\sigma }}^c}|\Phi _\alpha |) * \rho _0)(\boldsymbol{x} - \boldsymbol{z})} {\, \mathrm d}\boldsymbol{z} \Big )^2 \\&\qquad \qquad \qquad \qquad \qquad + \int _{\mathbb {R}^n} \varepsilon ^2 e^{-2|\boldsymbol{y}|^2} (1\!\!1_{B_{\hat{\sigma }}^c}|\Phi _\alpha |)(\boldsymbol{x} - \boldsymbol{y}) {\, \mathrm d}\boldsymbol{y} \bigg ) \\&\quad \le 4 \Big (\big \Vert (1\!\!1_{B_{\hat{\sigma }}^c} |\Phi _\alpha |)*\rho _0\big \Vert _{L^\infty (B_{D + 1})} + \big \Vert (1\!\!1_{B_{\hat{\sigma }}^c} |\Phi _\alpha |)*e^{-2|\,\cdot \,|^2} \big \Vert _{L^\infty (B_{D + 1})} \Big ). \end{aligned} \end{aligned}$$

This yields

B.7 $$\begin{aligned} I_2 \le 5 \big \Vert (1\!\!1_{B_{\hat{\sigma }}^c} |\Phi _\alpha |)*\rho _0 \big \Vert _{L^\infty (B_{D + 1})} + 4 \big \Vert (1\!\!1_{B_{\hat{\sigma }}^c} |\Phi _\alpha |)*e^{-2|\cdot |^2} \big \Vert _{L^\infty (B_{D + 1})} \longrightarrow 0 \qquad \text { as}\quad {\hat{\sigma }} \rightarrow \infty . \end{aligned}$$

Thus, combining (B.6) with (B.7), we obtain

$$\begin{aligned} \big \Vert \Phi _\alpha *\rho _0^\varepsilon - \Phi _\alpha * \rho _0 \big \Vert _{L^\infty (K)} \longrightarrow 0 \qquad \text { as}\quad \varepsilon \rightarrow 0. \end{aligned}$$

For the final part, take $1< p < \min \{\frac{2n}{\alpha + 2},\gamma \}$, by applying the HLS inequality from Lemma A.2, the interpolation argument, and (3.7), we have

$$\begin{aligned} \begin{aligned}&\big \Vert \nabla (\Phi _\alpha *\rho ^\varepsilon _0 - \Phi _\alpha * \rho _0)\big \Vert _{L^\frac{2n}{\alpha + 2}(K)} \\&\quad \le \big \Vert (|\cdot |^{-(\alpha + 1)} 1\!\!1_{B_1}) * (\rho ^\varepsilon _0 - \rho _0) \big \Vert _{L^\frac{2n}{\alpha + 2}(K)} + \big \Vert (|\cdot |^{-(\alpha + 1)} 1\!\!1_{B_1^c}) * (\rho ^\varepsilon _0 - \rho _0) \big \Vert _{L^\frac{2n}{\alpha + 2}(K)} \\&\quad \le \big \Vert (|\cdot |^{-(1 + \alpha /2 + n(1-1/p))}) * (\rho ^\varepsilon _0 - \rho _0) \big \Vert _{L^\frac{2n}{\alpha + 2}(K)} + \big \Vert 1\!\!1_{B_1^c} * (\rho ^\varepsilon _0 - \rho _0)\big \Vert _{L^\frac{2n}{\alpha + 2}(K)} \\&\quad \le C_{n,1 + \alpha /2 + n(1-1/p)} \Vert \rho ^\varepsilon _0 - \rho _0\Vert _{L^p(\mathbb {R}^n)} + |K|^{\frac{\alpha + 2}{2n}} \Vert \rho ^\varepsilon _0 - \rho _0\Vert _{L^1(\mathbb {R}^n)} \\&\quad \le C_{n,1 + \alpha /2 + n(1-1/p)} \Vert \rho ^\varepsilon _0 - \rho _0\Vert _{L^1(\mathbb {R}^n)}^{\frac{\gamma - p}{p(\gamma - 1)}} \Vert \rho ^\varepsilon _0 - \rho _0\Vert _{L^\gamma (\mathbb {R}^n)}^{\frac{\gamma (p-1)}{p(\gamma - 1)}} + |K|^{\frac{\alpha + 2}{2n}} \Vert \rho ^\varepsilon _0 - \rho _0\Vert _{L^1(\mathbb {R}^n)}. \end{aligned} \end{aligned}$$

Thus, we conclude

$$\begin{aligned} \Vert \nabla (\Phi _\alpha *\rho ^\varepsilon _0 - \Phi _\alpha * \rho _0)\Vert _{L^\frac{2n}{\alpha + 2}(K)} \longrightarrow 0 \qquad \text { as}\, \varepsilon \rightarrow 0 . \end{aligned}$$

Now, by using the initial assumption (2.3), there exists C>0 such that

$$ \int _{\mathbb {R}^n} \rho _0(\boldsymbol{x}) k_\alpha (1 + |\boldsymbol{x}|^2) {\, \mathrm d}\boldsymbol{x} \le C. $$

Thus, applying the Hölder inequality, we have

$$\begin{aligned} \begin{aligned}&\int _{\mathbb {R}^n} \rho _0^\varepsilon (\boldsymbol{x}) k_\alpha (1 + |\boldsymbol{x}|^2) {\, \mathrm d}\boldsymbol{x} \\&\quad \le \int _{\mathbb {R}^n} \rho _0(\boldsymbol{x}) k_\alpha (1 + |\boldsymbol{x}|^2) {\, \mathrm d}\boldsymbol{x} + \Big |\int _{\mathbb {R}^n} \big (\rho _0^\varepsilon (\boldsymbol{x}) - \rho _0(\boldsymbol{x})\big ) k_\alpha (1 + |\boldsymbol{x}|^2) {\, \mathrm d}\boldsymbol{x} \Big | \\&\quad \le C + \Big |\int _{B_1} \big (\rho _0^\varepsilon (\boldsymbol{x}) - \rho _0(\boldsymbol{x})\big ) k_\alpha (1 + |\boldsymbol{x}|^2) {\, \mathrm d}\boldsymbol{x} \Big | \\&\qquad + \Big |\int _{B_1^c} \big (\rho _0^\varepsilon (\boldsymbol{x}) - \rho _0(\boldsymbol{x})\big ) k_\alpha (1 + |\boldsymbol{x}|^2) {\, \mathrm d}\boldsymbol{x} \Big |\\&\quad \le C + k_\alpha (2) \Vert \rho _0^\varepsilon - \rho _0\Vert _{L^1(B_1)} + \Big |\int _{B_1^c} \big (\rho _0^\varepsilon (\boldsymbol{x}) - \rho _0(\boldsymbol{x})\big ) k_\alpha (2|\boldsymbol{x}|^2) {\, \mathrm d}\boldsymbol{x} \Big |\\&\quad \le C + k_\alpha (2) \Vert \rho _0^\varepsilon - \rho _0\Vert _{L^1(B_1)} + k_\alpha (2) \Vert \rho _0^\varepsilon - \rho _0\Vert _{L^1(B_1^c)} \\&\qquad + C \Big |\int _{B_1^c} \big (\rho _0^\varepsilon (\boldsymbol{x}) - \rho _0(\boldsymbol{x})\big ) k_\alpha (|\boldsymbol{x}|^2) {\, \mathrm d}\boldsymbol{x}\Big |\\&\quad = C\big ( 1 + \Vert \rho _0^\varepsilon - \rho _0\Vert _{L^1(\mathbb {R}^n)} + |\Phi _\alpha * (\rho _0^\varepsilon - \rho _0)|(\boldsymbol{0})\big ). \end{aligned} \end{aligned}$$

Now, by (B.2) and (B.4), we have

$$ \Vert \rho _0^\varepsilon - \rho _0\Vert _{L^1(\mathbb {R}^n)} + |\Phi _\alpha * (\rho _0^\varepsilon - \rho _0)|(\boldsymbol{0}) \longrightarrow 0 \qquad \text {as } \varepsilon \rightarrow 0. $$

Then there exists some C>0 such that, for small enough ε, (B.5) holds. □

As in the previous literature, in order to prove the BD entropy and the convergence of $\Omega ^T$ to $[0,T] \times (0,\infty )$ in some sense, we require that

B.8 $$\begin{aligned} \rho _0^{\varepsilon ,b}(b) \cong b^{-(n-\beta )}, \end{aligned}$$

where $\beta = \min \{ \frac{1}{2}, (1-\frac{1}{\gamma })n\}$. To do this, we choose a cut-off function $S \in C^\infty (\mathbb {R})$ such that

$$\begin{aligned} {\left\{ \begin{array}{ll} S(z) = 0 \quad \text { if } z \in (-\infty ,0], \\ S(z) = 1 \quad \text { if } z \in [1,\infty ), \\ S(z) \text { is monotonic in } [0,1]. \\ \end{array}\right. } \end{aligned}$$

Now, as in [26], we define

$$\begin{aligned} \tilde{\rho }^{\varepsilon ,b}_0(\boldsymbol{x}) {:=}\Big \{ \sqrt{\rho _0^{\varepsilon }(\boldsymbol{x})}\big (1 - S(2(|\boldsymbol{x}| - (b-1)))\big ) + b^{-\frac{n-\beta }{2}}S(2(|\boldsymbol{x}| - (b-1))) \Big \}^2. \end{aligned}$$

Then $\tilde{\rho }^{\varepsilon ,b}_0$ is a radial function and satisfies $\tilde{\rho }^{\varepsilon ,b}_0(b) = b^{-(n-\beta )}$. Now, as before, we normalize $\tilde{\rho }^{\varepsilon ,b}_0$ (but on the corresponding domain from problem (2.10)–(2.13): the annulus $\{b^{-1} \le |\boldsymbol{x}| \le b\}$) to produce

B.9 $$\begin{aligned} \rho ^{\varepsilon ,b}_0(\boldsymbol{x}) {:=}\frac{M}{\int _{b^{-1} \le |\boldsymbol{x}| \le b} \tilde{\rho }^{\varepsilon ,b}_0(\boldsymbol{x}) {\, \mathrm d}\boldsymbol{x}} 1\!\!1_{\{b^{-1} \le |\boldsymbol{x}| \le b \}}(\boldsymbol{x}) \tilde{\rho }^{\varepsilon ,b}_0(\boldsymbol{x}). \end{aligned}$$

As before, it is direct to see that (B.8) is satisfied. Similar to [26], one can show the following lemma:

### Lemma B.3

The defined function $\rho ^{\varepsilon ,b}_0(\boldsymbol{x})$ in (B.9) is smooth. Moreover, for any $q \in \{1,\gamma \}$ when κ=1 and $q \in \{1,\gamma ,\frac{2n}{2n - \alpha }\}$ when κ=-1, the following estimates hold:

$$\begin{aligned} \begin{aligned}&\int _{b^{-1}\le |\boldsymbol{x}|\le b} \rho _{0}^{\varepsilon ,b}(\boldsymbol{x})\,{\, \mathrm d}\boldsymbol{x}= M \qquad \text {for all } \varepsilon \in (0,1] \text { and } b>1 ,\\&\int _{|\boldsymbol{x}|\le b} \Big (\big |\rho _0^{\varepsilon ,b}(\boldsymbol{x})-\rho _0^\varepsilon (\boldsymbol{x})\big |^q +\Big | \sqrt{\rho _{0}^{\varepsilon ,b}(\boldsymbol{x})} -\sqrt{\rho _{0}^{\varepsilon }(\boldsymbol{x})}\Big |^{2q}\Big )\,{\, \mathrm d}\boldsymbol{x}\rightarrow 0 \qquad \text {as } b\rightarrow \infty ,\\&\varepsilon \int _{|\boldsymbol{x}|\le b}\Big |\nabla _{\boldsymbol{x}}\sqrt{\rho _0^{\varepsilon ,b}(\boldsymbol{x})}\Big |^2 {\, \mathrm d}\boldsymbol{x} \le C\big (\Vert \rho _0\Vert _{L^1}+1\big ). \end{aligned} \end{aligned}$$

We also have the convergence of the initial potentials, represented by the following lemma:

### Lemma B.4

$$\Phi _\alpha *\rho ^{\varepsilon ,b}_0$$ with $$\alpha \in (0,n-1)$$ satisfy

$$\begin{aligned} \begin{aligned}&\Vert \Phi _\alpha *\rho _0^{\varepsilon ,b} - \Phi _\alpha * \rho _0^\varepsilon \Vert _{L^{\frac{2n}{\alpha }}} \le C \Vert \rho ^{\varepsilon ,b}_0 - \rho ^\varepsilon _0\Vert _{L^\frac{2n}{2n - \alpha }} \longrightarrow 0 \qquad \text { as}\, b \rightarrow \infty , \\&\Vert \nabla (\Phi _\alpha *\rho ^{\varepsilon ,b}_0 - \Phi _\alpha * \rho ^\varepsilon _0)\Vert _{L^\frac{2n}{\alpha + 2}} \le C \Vert \rho ^{\varepsilon ,b}_0 - \rho ^\varepsilon _0\Vert _{L^\frac{2n}{2n - \alpha }} \longrightarrow 0 \qquad \text { as}\, b \rightarrow \infty . \end{aligned} \end{aligned}$$

For $$\alpha \in (-1,0]$$ and $$K \Subset \mathbb {R}^n$$,

$$\begin{aligned} \Vert \Phi _\alpha *\rho _0^{\varepsilon ,b} - \Phi _\alpha * \rho ^\varepsilon _0\Vert _{L^\infty (K)} + \Vert \nabla (\Phi _\alpha *\rho ^{\varepsilon ,b}_0 - \Phi _\alpha * \rho ^\varepsilon _0)\Vert _{L^\frac{2n}{\alpha + 2}(K)} \longrightarrow 0 \qquad \text { as}\, b \rightarrow \infty , \end{aligned}$$

Moreover, there exists a constant C>0 such that, for ε>0 sufficiently small and *b* sufficiently large,

B.10 $$\begin{aligned} \int _{\mathbb {R}^n} \rho _0^{\varepsilon ,b}(\boldsymbol{x}) k_\alpha (1 + |\boldsymbol{x}|^2) {\, \mathrm d}\boldsymbol{x} \le C. \end{aligned}$$

### Proof

The first part is an application of the HLS inequality from Lemma A.2. The second part follows the same approach as for Lemma B.2. □

We now provide an approximation to the initial velocity. To avoid issues regarding the vacuum, we construct $u^\varepsilon _0$ as

B.11 $$\begin{aligned} u^\varepsilon _0(\boldsymbol{x}) {:=}\frac{1}{\sqrt{\rho _0^\varepsilon (\boldsymbol{x})}} \Big ( \frac{m_0}{\sqrt{\rho _0}} \Big )(\boldsymbol{x}). \end{aligned}$$

As in [26, Lemma A.8], we can prove the following:

### Lemma B.5

As defined above, $u^\varepsilon _0$ satisfies

$$\begin{aligned}&\int _{\mathbb {R}^n} \rho ^\varepsilon _0(\boldsymbol{x})|u_0^\varepsilon (\boldsymbol{x})|^2 {\, \mathrm d}\boldsymbol{x} = \int _{\mathbb {R}^n} \frac{|m_0(\boldsymbol{x})|^2}{\rho _0(\boldsymbol{x})} {\, \mathrm d}\boldsymbol{x}, \\&\,\,\,\big \Vert \rho _0^\varepsilon u^\varepsilon _0 - m_0\big \Vert _{L^1} \longrightarrow 0 \qquad \text { as}\quad \varepsilon \rightarrow 0. \end{aligned}$$

We can now define $\tilde{u}^{\varepsilon ,b}_0$:

$$\begin{aligned} \tilde{u}^{\varepsilon ,b}_0(\boldsymbol{x}) = \frac{1}{\sqrt{\rho ^{\varepsilon ,b}_0(\boldsymbol{x})}} \int _{\mathbb {R}^n} \Big ( \frac{m_0 1\!\!1_{[4b^{-1},b - 2]}}{\sqrt{\rho _0}} \Big )(\boldsymbol{x} - \boldsymbol{y}) J_{b^{-1}}(\boldsymbol{y}) {\, \mathrm d}\boldsymbol{y}, \end{aligned}$$

where we have used the indicator function $1\!\!1_{[4b^{-1},\,b - 2]}$ so that $\textrm{supp}(\tilde{u}^{\varepsilon ,b}_0) \subset [2b^{-1},\,b-1]$, which implies that the lower boundary condition is satisfied. We can now add the stress-free boundary condition to the upper boundary. In order to satisfy (2.12), we construct an initial velocity that satisfies

$$\begin{aligned} \Big ( p(\rho ^{\varepsilon ,b}_0) - \varepsilon \rho _0^{\varepsilon ,b} (u^{\varepsilon ,b}_{0,r} + \frac{n-1}{r}u^{\varepsilon ,b}_0 ) \Big )(r) = 0 \qquad \text {for } r \in (b - \frac{1}{4},b]. \end{aligned}$$

Given a solution to the above, then

$$\begin{aligned} (u^{\varepsilon ,b}_0r^{n-1})_r = \frac{1}{\varepsilon } \frac{p(\rho ^{\varepsilon ,b}_0(r))}{\rho ^{\varepsilon ,b}_0(r)} r^{n-1} \qquad \text {for } r \in (b - \frac{1}{4},b). \end{aligned}$$

Integrating over (*r*, *b*) and choosing $u^{\varepsilon ,b}_0(b) = 0$, we obtain

$$\begin{aligned} u^{\varepsilon ,b}_0(r) = -\frac{1}{\varepsilon }\frac{1}{r^{n-1}}\int ^b_r\frac{p(\rho ^{\varepsilon ,b}_0(z))}{\rho ^{\varepsilon ,b}_0(z)} z^{n-1} {\, \mathrm d}z. \end{aligned}$$

Hence, in order to add this boundary condition to $\tilde{u}^{\varepsilon ,b}_0$, we use the construction from [26]:

B.12 $$\begin{aligned} u^{\varepsilon ,b}_0(\boldsymbol{x}) {:=}\tilde{u}^{\varepsilon ,b}_0(\boldsymbol{x}) - \frac{1}{\varepsilon } S(4(|\boldsymbol{x}| - (b - \frac{1}{2}))) \frac{1}{|\boldsymbol{x}|^{n-1}} \int ^b_{|\boldsymbol{x}|} \frac{p(\rho ^{\varepsilon ,b}_0(z))}{\rho ^{\varepsilon ,b}_0(z)} z^{n-1} {\, \mathrm d}z. \end{aligned}$$

Then $u^{\varepsilon ,b}_0$ satisfies (2.12). As in [26, Lemma A.9], we obtain the following lemma:

### Lemma B.6

For given ε>0,

$$\begin{aligned}&\lim _{b\rightarrow \infty }\int _{|\boldsymbol{x}|\le b} \Big |\sqrt{\rho _{0}^{\varepsilon ,b}(\boldsymbol{x})}u_{0}^{\varepsilon ,b}(\boldsymbol{x})\Big |^2{\, \mathrm d}\boldsymbol{x} =\int _{\mathbb {R}^n} \Big |\sqrt{\rho _0^\varepsilon (\boldsymbol{x})} u_0^\varepsilon (\boldsymbol{x})\Big |^2{\, \mathrm d}\boldsymbol{x},\\&\sqrt{\rho _{0}^{\varepsilon ,b}(\boldsymbol{x})}u_{0}^{\varepsilon ,b}(\boldsymbol{x})\longrightarrow \sqrt{\rho _{0}^{\varepsilon }(\boldsymbol{x})}u_{0}^{\varepsilon }(\boldsymbol{x}) \qquad \text {in } L^2(\{|\boldsymbol{x}|\le b\})\, \text { as } b\rightarrow \infty . \end{aligned}$$

We define the approximate initial energy as follows:

### Definition B.7

Define the energy for the approximate initial data by

$$\begin{aligned} E_0^\varepsilon {:=}\int _{\mathbb {R}^n} \bigg ( \frac{1}{2} \rho _0^{\varepsilon }\big |u^{\varepsilon }_0\big |^2 + \rho _0^\varepsilon e(\rho _0^\varepsilon ) + \frac{\kappa }{2} \rho _0^\varepsilon (\Phi _\alpha * \rho _0^\varepsilon ) \bigg )(\boldsymbol{x}) {\, \mathrm d}\boldsymbol{x} < \infty , \end{aligned}$$

and

$$\begin{aligned} E_0^{\varepsilon ,b} {:=}\int _{\mathbb {R}^n} \bigg ( \frac{1}{2} \rho _0^{\varepsilon ,b} \big |u^{\varepsilon ,b}_0\big |^2 + \rho _0^{\varepsilon ,b} e(\rho _0^{\varepsilon ,b}) + \frac{\kappa }{2}\rho _0^{\varepsilon ,b}(\Phi _\alpha *\rho _0^{\varepsilon ,b})\bigg )(\boldsymbol{x}) {\, \mathrm d}\boldsymbol{x} < \infty . \end{aligned}$$

Combining all together, we obtain the following lemma:

### Lemma B.8

Take $\rho ^\varepsilon _0,\rho ^{\varepsilon ,b}_0,u^\varepsilon _0$, and $u^{\varepsilon ,b}_0$ to be defined as in (B.1), (B.9), (B.11), and (B.12) respectively. Then $(\rho ^{\varepsilon ,b}_0,u^{\varepsilon ,b}_0) \in C^\infty ([b^{-1},b])$ and satisfies (2.12). For $q \in \{1,\gamma ,\frac{2n}{2n - \alpha }\}$ for κ=-1 and α>0, and $q \in \{1,\gamma \}$ otherwise, then the following statements hold:

- For all $$\varepsilon \in (0,1]$$,
  $$\begin{aligned} \int _0^\infty \rho _0^\varepsilon (r)\,\omega _n r^{n-1} {\, \mathrm d}r=M,\qquad \varepsilon ^2\int _{0}^\infty |\partial _r\sqrt{\rho ^\varepsilon _{0}(r)}|^2\, r^{n-1}{\, \mathrm d}r \le C\varepsilon (M+1). \end{aligned}$$
  As $$\varepsilon \rightarrow 0$$,
  $$\begin{aligned} {\begin{matrix} & E^\varepsilon _0\longrightarrow E_0, \\ & (\rho _0^{\varepsilon }, m_0^{\varepsilon })(r)\longrightarrow (\rho _0, m_0)(r) \quad \text{ in } L^q([0,\infty ); r^{n-1}{\, \mathrm d}r)\times L^1([0,\infty ); r^{n-1}{\, \mathrm d}r) ,\\ & (\Phi _\alpha * \rho ^\epsilon _0)_r \rightarrow (\Phi _\alpha * \rho _0)_r \qquad \,\,\text { in } L^{\frac{2n}{\alpha +2}}([0,\infty );r^{n-1}{\, \mathrm d}r)\,\, \text { for } \,\alpha \in (0,n-1) . \end{matrix}} \end{aligned}$$
  For $\alpha \in (-1,0]$, the convergence of $\Phi _\alpha *\rho _0^{\varepsilon }$ and its gradient is understood in the local sense stated in Lemma B.2. Then there exists $\varepsilon _0 > 0$ such that
  $$\begin{aligned} M<M_\textrm{c}^{\varepsilon ,\alpha }(\gamma ) \qquad \text {for all } \varepsilon \in (0,\varepsilon _0] \text { and } \gamma \in (\frac{2n}{2n - \alpha }, \frac{n + \alpha }{n}]. \end{aligned}$$
- For all $$\varepsilon \in (0,1]$$,
  $$\begin{aligned} \int _{b^{-1}}^{b} \rho _{0}^{\varepsilon ,b}(r)\,\omega _n r^{n-1} {\, \mathrm d}r= M, \qquad \varepsilon ^2\int _{b^{-1}}^b \big |\partial _r\sqrt{\rho ^{\varepsilon ,b}_{0}(r)}\big |^2\, r^{n-1}{\, \mathrm d}r \le C\varepsilon (M+1). \end{aligned}$$
  Moreover, for any fixed ε>0, as $b\rightarrow \infty$,
  $$\begin{aligned} {\begin{matrix} &  E_0^{\varepsilon ,b} \longrightarrow E_0^\varepsilon , \\ & (\rho _0^{\varepsilon ,b}, m_0^{\varepsilon ,b})(r)\longrightarrow (\rho _0^\varepsilon , m^\varepsilon _0)(r) \qquad \text{ in } L^q([0, b]; r^{n-1}{\, \mathrm d}r)\times L^1([0, b]; r^{n-1}{\, \mathrm d}r) ,\\ & (\Phi _\alpha * \rho ^{\epsilon ,b}_0)_r \rightarrow (\Phi _\alpha * \rho ^\epsilon _0)_r \qquad \qquad \text{ in } L^{\frac{2n}{\alpha +2}}([0,\infty );r^{n-1}{\, \mathrm d}r)\,\, \text{ for } \,\alpha \in (0,n-1) . \end{matrix}} \end{aligned}$$
  For $\alpha \in (-1,0]$, the convergence of $\Phi _\alpha *\rho _0^{\varepsilon , b}$ and its gradient is understood in the local sense stated in Lemma B.4. Then there exists $\mathfrak {B}(\varepsilon ) > 0$ such that
  $$\begin{aligned} M<M_\textrm{c}^{\varepsilon ,b,\alpha }(\gamma ) \qquad \text {for all } \varepsilon \in (0,\varepsilon _0], b \ge \mathfrak {B}(\varepsilon ), \text { and } \gamma \in (\frac{2n}{2n - \alpha }, \frac{n + \alpha }{n}]. \end{aligned}$$

## References

[1] Alberti, G., Choksi, R., Otto, F.: Uniform energy distribution for an isoperimetric problem with long-range interactions. J. Amer. Math. Soc. **22**(2), 569–605 (2009)

[2] Alves, N.J., Grafakos, L., Tzavaras, A.E.: A bilinear fractional integral operator for Euler–Riesz systems, Preprint arXiv:2409.18309 (2024)

[3] Anderson, G.W., Guionnet, A., Zeitouni, O.: An Introduction to Random Matrices, Cambridge Studies in Advanced Mathematics, 118. Cambridge University Press, Cambridge (2010)

[4] Auchmuty, J.F.G., Beals, R.: Variational solutions of some nonlinear free boundary problems. Arch. Ration. Mech. Anal. **43**, 255–271 (1971)

[5] Balagué, D., Carrillo, J.A., Laurent, T., Raoul, G.: Nonlocal interactions by repulsive-attractive potentials: radial ins/stability. Phys. D. **260**, 5–25 (2013)

[6] Bianchini, S., Bressan, A.: Vanishing viscosity solutions of nonlinear hyperbolic systems. Ann. Math. **161**(1), 223–342 (2005)

[7] Blanchet, A., Carrillo, J.A., Laurençot, P.: Critical mass for a Patlak–Keller–Segel model with degenerate diffusion in higher dimensions. Calc. Var. Partial Differential Equations **35**, 133–168 (2009)

[8] Bobylev, A.V., Dukes, P., Illner, R., Victory, H.D.: On Vlasov–Manev equations, I. Foundations, properties, and nonglobal existence. J. Stat. Phys. **88**, 885–911 (1997)

[9] Bresch, D., Desjardins, B.: On viscous shallow-water equations (Saint-Venant model) and the quasi-geostrophic limit. C. R. Math. Acad. Sci. Paris **335**, 1079–1084 (2002)

[10] Bresch, D., Desjardins, B., Lin, C.K.: On some compressible fluid models: Korteweg, lubrication, and shallow water systems. Commun. Partial Differ. Equ. **28**, 843–868 (2003)

[11] Calvez, V., Carrillo, J.A., Hoffmann, F.: Equilibria of homogeneous functionals in the fair-competition regime. Nonlinear Anal. **159**, 85–128 (2017)

[12] Calvez, V., Carrillo, J.A., Hoffmann, F.: Uniqueness of stationary states for singular Keller–Segel type models. Nonlinear Anal. **205**, 112222 (2021)

[13] Campa, A., Dauxois, T., Fanelli, D., Ruffo, S.: Physics of Long-Range Interacting Systems. Oxford University Press, Oxford (2014)

[14] Carrillo, J.A., Castorina, D., Volzone, B.: Ground states for diffusion dominated free energies with logarithmic interaction. SIAM J. Math. Anal. **47**, 1–25 (2015)

[15] Carrillo, J.A., Choi, Y.-P.: Mean-Field Limits: From particle descriptions to macroscopic equations. Arch. Rational Mech. Anal. **241**(3), 1529–1573 (2021) 10.1007/s00205-021-01676-x PMC8550335 34720114

[16] Carrillo, J.A., Choi, Y.-P., Perez, S.P.: A review on attractive—repulsive hydrodynamics for consensus in collective behavior. In: Active Particles, Vol. 1: Advances in Theory, Models, and Applications, pp. 259–298. Birkhäuser/Springer, Cham (2017)

[17] Carrillo, J.A., Hoffmann, F., Mainini, E., Volzone, B.: Ground states in the diffusion-dominated regime. Calc. Var. Partial Differential Equations **57**, 127 (2018) 10.1007/s00526-018-1402-2 PMC6190998 30393443

[18] Carrillo, J.A., Hittmeir, S., Volzone, B., Yao, Y.: Nonlinear aggregation-diffusion equations: radial symmetry and long time asymptotics. Invent. Math. **218**, 889–977 (2019)

[19] Carrillo, J.A., Craig, K., Yao, Y.: Aggregation-diffusion equations: dynamics, asymptotics, and singular limits. In: Active Particles, Vol. 2: Advances in Theory, Models, and Applications, Model. Simul. Sci. Eng. Technol., pp. 65–108. Birkhäuser/Springer, Cham (2019)

[20] Carrillo, J.A., Shu, R.W.: From radial symmetry to fractal behavior of aggregation equilibria for repulsive–attractive potentials. Calc. Var. Partial Differential Equations **62**, 28 (2023)

[21] Cercignani, C., Illner, R., Pulvirenti, M.: The Mathematical Theory of Dilute Gases, Applied Mathematical Sciences, vol. 106. Springer-Verlag, New York (1994)

[22] Chan, H., González, M.D.M., Huang, Y.-H., Mainini, E., Volzone, B.: Uniqueness of entire ground states for the fractional plasma problem. Calc. Var. Partial Differential Equations **59**, 195 (2020)

[23] Chandrasekhar, S.: An Introduction to the Study of Stellar Structures. University of Chicago Press, Chicago (1938)

[24] Chen, G.-Q.: Convergence of the Lax-Friedrichs scheme for isentropic gas dynamics (III). Acta Math. Sci. **6B**(1986), 75–120 (in English); **8A**, 243–276 (1988) (in Chinese)

[25] Chen, G.-Q., Feldman, M.: The Mathematics of Shock Reflection-Diffraction and von Neumann’s Conjectures, Research Monograph, Annals of Mathematics Studies, vol. 197. Princeton University Press, Princeton (2018)

[26] Chen, G.-Q., He, L., Wang, Y., Yuan, D.F.: Global solutions of the compressible Euler–Poisson equations with large initial data of spherical symmetry. Comm. Pure Appl. Math. **77**(6), 2947–3025 (2024) 10.1007/s00220-026-05659-5 PMC13524983 42668740

[27] Chen, G.-Q., Perepelitsa, M.: Vanishing viscosity limit of the Navier–Stokes equations to the Euler equations for compressible fluid flow. Comm. Pure Appl. Math. **63**, 1469–1504 (2010)

[28] Chen, G.-Q., Perepelitsa, M.: Vanishing viscosity limit solutions of the compressible Euler equations with spherical symmetry and large initial data. Commun. Math. Phys. **338**, 771–800 (2015)

[29] Chen, G.-Q., Schrecker, M.: Vanishing viscosity approach to the compressible Euler equations for transonic nozzle and spherically symmetric flows. Arch. Ration. Mech. Anal. **229**, 1239–1279 (2018)

[30] Chen, G.-Q., Wang, Y.: Global solutions of the compressible Euler equations with large initial data of spherical symmetry and positive far-field density. Arch. Ration. Mech. Anal. **243**, 1699–1771 (2022)

[31] Choi, Y.-P., Jeong, I.-J.: On well-posedness and singularity formation for the Euler–Riesz system. J. Differ. Equ. **306**, 296–332 (2022)

[32] Choi, Y.-P., Jung, J.: The pressureless damped Euler–Riesz equations. Ann. Inst. H. Poincaré Anal Non Linéaire **40**, 593–630 (2023)

[33] Choi, Y.-P., Jung, J., Lee, Y.: The global Cauchy problem for the Euler–Riesz equations. Nonlinear Anal. **253**, 113724 (2025)

[34] Cotar, C., Friesecke, G., Klüppelberg, C.: Density functional theory and optimal transportation with Coulomb cost. Comm. Pure Appl. Math. **66**(4), 548–599 (2013)

[35] Cotar, C., Petrache, M.: Next-order asymptotic expansion for N-marginal optimal transport with Coulomb and Riesz costs. Adv. Math. **344**, 137–233 (2019)

[36] Dafermos, C.M.: Hyperbolic Conservation Laws in Continuum Physics. Springer-Verlag, Berlin (2016)

[37] Danchin, R., Ducomet, B.: On the global existence for the compressible Euler–Riesz system. J. Math. Fluid Mech. **24**, 48 (2022)

[38] Ding, X., Chen, G.-Q., Luo, P.: Convergence of the Lax-Friedrichs scheme for the isentropic gas dynamics (I)–(II). Acta Math. Sci. **5B**, 483–500, 501–540 (1985) (in English); **7A**, 467–480 (1987); **8A**, 61–94 (1989) (in Chinese)

[39] DiPerna, R.J.: Convergence of the viscosity method for isentropic gas dynamics. Commun. Math. Phys. **91**, 1–30 (1983)

[40] Delgadino, M., Yan, X., Yao, Y.: Uniqueness and nonuniqueness of steady states of aggregation-diffusion equations. Comm. Pure Appl. Math. **75**, 3–59 (2020)

[41] Deng, Y., Liu, T., Yang, T., Yao, Z.: Solutions of Euler-Poisson equations for gaseous stars. Arch. Ration. Mech. Anal. **164**, 261–285 (2002)

[42] Duan, Q., Li, H.-L.: Global existence of weak solution for the compressible Navier–Stokes–Poisson system for gaseous stars. J. Differ. Equ. **259**, 5302–5330 (2015)

[43] Galdi, G.P.: An Introduction to the Mathematical Theory of the Navier–Stokes Equations: Steady-State Problems. Springer Monographs in Mathematics, 2nd edn. Springer, New York (2011)

[44] Gelfand, I. M., Shilov, G.E.: Generalized Functions, Vol. 1: Properties and Operations, AMS Chelsea Publishing, Providence, RI, 2016. Translated from the 1958 Russian original [MR0097715] by Eugene Saletan, Reprint of the 1964 English translation [MR0166596]

[45] Gilbarg, D.: The existence and limit behavior of the one-dimensional shock layer. Amer. J. Math. **73**, 256–274 (1951)

[46] Glimm, J.: Solutions in the large for nonlinear hyperbolic systems of equations. Comm. Pure Appl. Math. **18**(4), 697–715 (1965)

[47] Goldreich, P., Weber, S.: Homologously collapsing stellar cores. Astrophys. J. **238**, 991–997 (1980)

[48] Guo, Y.: Smooth irrotational flows in the large to the Euler-Poisson system in . Commun. Math. Phys. **195**, 249–265 (1998)

[49] Guo, Y., Hadzic, M., Jang, J.: Larson–Penston self-similar gravitational collapse. Commun. Math. Phys. **386**, 1551–1601 (2021) 10.1007/s00220-021-04175-y PMC8550190 34720128

[50] Guo, Y., Hadzic, M., Jang, J.: Continued gravitational collapse for Newtonian stars. Arch. Ration. Mech. Anal. **239**, 431–552 (2021)

[51] Guo, Y., Hadzic, M., Jang, J., Schrecker, M.: Gravitational collapse for polytropic gaseous stars: self-similar solutions. Arch. Ration. Mech. Anal. **246**, 957–1066 (2022)

[52] Guo, Y., Ionescu, A.D., Pausader, B.: Global solutions of the Euler-Maxwell two-fluid system in 3D. Ann. Math. (2) **183**, 377–498 (2016)

[53] Guo, Y., Pausader, B.: Global smooth ion dynamics in the Euler–Poisson system. Commun. Math. Phys. **303**, 89–125 (2011)

[54] Hoff, D., Liu, T.-P.: The inviscid limit for the Navier–Stokes equations of compressible, isentropic flow with shock data. Indiana Univ. Math. J. **38**, 861–915 (1989)

[55] Hadzić, M., Jang, J.: Nonlinear stability of expanding star solutions of the radially symmetric mass-critical Euler–Poisson system. Comm. Pure Appl. Math. **71**, 827–891 (2018)

[56] Hadzic, M., Jang, J.: A class of global solutions to the Euler–Poisson system. Commun. Math. Phys. **370**, 475–505 (2019)

[57] Hardin, D.P., Leblé, T., Saff, E.B., Serfaty, S.: Large deviation principles for hypersingular Riesz gases. Constr. Approx. **48**(1), 61–100 (2018)

[58] Huang, F.M., Wang, Z.: Convergence of viscosity solutions for isothermal gas dynamics. SIAM J. Math. Anal. **34**, 595–610 (2002)

[59] Illner, R.: Stellar dynamics, and plasma physics with corrected potentials: Vlasov, Manev, Boltzmann, Smoluchowski, hydrodynamic limits and related topics. Fields Inst. Comm. Amer. Math. Soc. **27**, 95–108 (2000)

[60] Ionescu, A.D., Pausader, B.: The Euler-Poisson system in 2D: global stability of the constant equilibrium solution. Int. Math. Res. Not. **4**, 761–826 (2013)

[61] Jang, J.: Nonlinear instability in gravitational Euler-Poisson system for . Arch. Ration. Mech. Anal. **188**, 265–307 (2008)

[62] Jang, J.: Nonlinear instability theory of Lane-Emden stars. Comm. Pure Appl. Math. **67**, 1418–1465 (2014)

[63] Lax, P.D.: Shock wave and entropy. In: Zarantonello, E.A. (ed.) Contributions to Functional Analysis, pp. 603–634. Academic Press, New York (1971)

[64] LeFloch, P., Westdickenberg, M.: Finite energy solutions to the isentropic Euler equations with geometric effects. J. Math. Pures Appl. **88**, 386–429 (2007)

[65] Lewin, M., Lieb, E.H., Seiringer, R.: Statistical mechanics of the uniform electron gas. Éc. Polytech. Math. **5**, 79–116 (2018)

[66] Lewin, M.: Coulomb and Riesz gases: the known and the unknown. J. Math. Phys. **63**(6), 061101 (2022)

[67] Lieb, E.H., Lebowitz, J.L.: Existence of thermodynamics for real matter with coulomb forces. Phys. Rev. Lett. **22**, 631–634 (1969)

[68] Lieb, E.H., Loss, M.: Analysis, 2nd edn. AMS, Providence (2001)

[69] Lieb, E.H., Seiringer, R.: The Stability of Matter in Quantum Mechanics. Cambridge University Press, Cambridge (2010)

[70] Lin, Z., Zeng, C.: Separable Hamiltonian PDEs and turning point principle for stability of gaseous stars. Comm. Pure Appl. Math. **75**, 2511–2572 (2022)

[71] Lin, Z., Wang, Y., Zhu, H.: Nonlinear stability of non-rotating gaseous stars. Math. Ann. (2024). 10.1007/s00208-024-02940-7

[72] Lions, P.-L.: The concentration-compactness principle in the calculus of variations: I. The locally compact case. Ann. Inst. H. Poincar Anal. Non Linaire, **2**, 109–145 (1984)

[73] Lions, P.-L., Perthame, B., Souganidis, P.: Existence and stability of entropy solutions for the hyperbolic systems of isentropic gas dynamics in Eulerian and Lagrangian coordinates. Comm. Pure Appl. Math. **49**, 599–638 (1996)

[74] Lions, P.-L., Perthame, B., Tadmor, E.: Kinetic formulation of the isentropic gas dynamics and p-systems. Commun. Math. Phys. **163**, 415–431 (1994)

[75] Luo, T., Smoller, J.: Nonlinear dynamical stability of Newtonian rotating and non-rotating white dwarfs and rotating supermassive stars. Commun. Math. Phys. **284**, 425–457 (2008)

[76] Luo, T., Smoller, J.: Existence and non-linear stability of rotating star solutions of the compressible Euler-Poisson equations. Arch. Ration. Mech. Anal. **191**, 447–496 (2009)

[77] Murat, F.: Compacité par compensation. Ann. Scuola Norm. Sup. Pisa Sci. Fis. Mat. ** 5**, 489–507 (1978)

[78] Nguyen, Q.H., Rosenzweig, M., Serfaty, S.: Mean-field limits of Riesz-type singular flows. Ars Inveniendi Analytica, vol. 4 (2022)

[79] Nicolaas Du Plessis, An Introduction to Potential Theory, University Mathematical Monographs, vol. 7, Hafner Publishing Co., Darien, CT, Oliver and Boyd, Edinburgh (1970)

[80] Rein, G.: Non-linear stability of gaseous stars. Arch. Ration. Mech. Anal. **168**(2), 115–130 (2003)

[81] Schrecker, M.: Spherically symmetric solutions of the multidimensional compressible, isentropic Euler equations. Trans. Amer. Math. Soc. **373**, 727–746 (2020)

[82] Serfaty, S.: Mean field limit for Coulomb-type flows (Appendix with Mitia Duerinckx). Duke Math. J. **169**(15), 2887–2935 (2020)

[83] Serfaty, S.: Lectures on Coulomb and Riesz gases, Preprint arXiv:2407.21194 (2024)

[84] Tartar, L.: Compensated compactness and applications to partial differential equations. In: Knops, R.J. (ed.) Research Notes in Mathematics, Nonlinear Analysis and Mechanics, Herriot-Watt Symposium, vol. 4. Pitman Press, Bath (1979)

